# Novel 1,4-Diazepane derivatives as amyloid-beta (Aβ) aggregation inhibitors

**DOI:** 10.3389/fchem.2026.1919936

**Published:** 2026-09-01

**Authors:** Rahul C. Karuturi, Ahmed A. Hefny, Arash Shakeri, Praveen P. N. Rao

**Affiliations:** School of Pharmacy, Health Sciences Campus, University of Waterloo, Waterloo, ON, Canada

**Keywords:** 1,4-diazepane, alzheimer’s disease, amyloid-beta aggregation, anti-amyloid agents, antioxidant activity, blood-brain barrier permeability, molecular docking, neuroprotection

## Abstract

**Introduction:**

Alzheimer’s disease (AD) is characterized by the aggregation of amyloid-beta (Aβ) peptides, particularly Aβ42 and Aβ40, which contribute to neurotoxicity and disease progression. The development of novel small molecule inhibitors capable of preventing Aβ aggregation represents a promising therapeutic strategy. We report the design, synthesis and evaluation of a novel class of 1,4-diazepanes as anti-amyloid agents.

**Methods:**

A novel series of (1,4-diazepan-1-yl) (phenyl)methanone derivatives (**4a–n**) incorporating a flexible seven-membered 1,4-diazepane scaffold was synthesized and screened for inhibition of Aβ42 and Aβ40 aggregation using a fluorescence-based thioflavin-T (ThT) kinetic assay. The effects of lead compounds on Aβ fibril morphology were subsequently examined by transmission electron microscopy (TEM). Neuroprotective potential was assessed by determining the ability of selected compounds to attenuate Aβ42-induced cytotoxicity in mouse hippocampal HT22 cells. Antioxidant activity was investigated using both the 2,2’-diphenyl-1-picryl-hydrazyl (DPPH) radical scavenging assay and hydrogen peroxide-induced oxidative stress models in HT22 cells. Blood–brain barrier permeability was evaluated using the parallel artificial membrane permeability assay (PAMPA-BBB). In addition, molecular docking studies were performed using Discovery Studio Structure-Based Design software using Aβ aggregates to characterize ligand–Aβ interactions and provide mechanistic insights into the observed anti-aggregation activity.

**Results:**

Among the synthesized derivatives, compound **4m** emerged as the lead Aβ42 aggregation inhibitor, demonstrating 42.0% inhibition at 25 µM. Notably, compounds **4k** and **4l** demonstrated dual inhibitory activity toward both Aβ42 and Aβ40 aggregation, with inhibition values of 40.4% and 57.3% for **4k**, and 36.1% and 59.2% for **4l**, respectively, at 25 µM. Compounds **4k–m** significantly reduced Aβ42-induced cytotoxicity in mouse hippocampal HT22 cells. In addition, compound **4l** exhibited antioxidant activity and attenuated hydrogen peroxide-induced cytotoxicity. Computational analyses suggested that these 1,4-diazepane derivatives interact with both the N- and C-termini regions of Aβ42 and Aβ40, stabilizing peptide assemblies and hindering further aggregation.

**Conclusion:**

These findings identify the 1,4-diazepane ring as a novel structural scaffold for the development of anti-amyloid agents. The dual inhibition of Aβ42 and Aβ40 aggregation, combined with neuroprotective and antioxidant effects, highlights compounds **4k–m** as attractive lead candidates for further optimization toward therapeutic intervention in Alzheimer’s disease.

## Graphical Abstract

**Figure:**
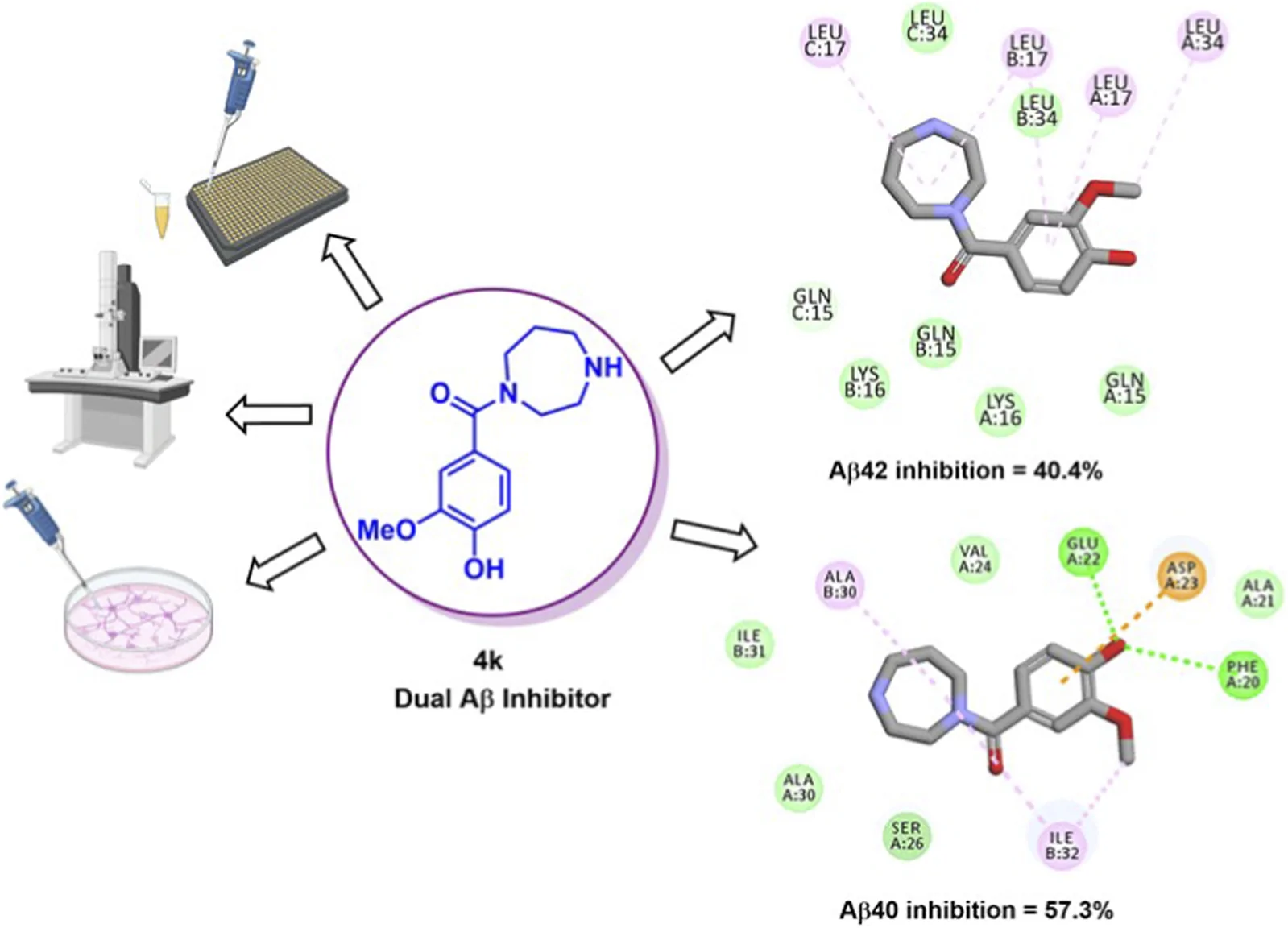

## 1 Introduction

Alzheimer’s disease (AD) is a multifactorial neurodegenerative disease that orchestrates the progressive disintegration of cognitive and behavioural functions (Courade et al., 2025; Knopman et al., 2021). One of the central hallmarks of AD pathology is the failure of the brain’s protein homeostasis systems, leading to the accumulation of neurotoxic amyloid beta (Aβ) aggregates (Karran and De Strooper, 2022; Selkoe and Hardy, 2016). Among the key contributors to AD progression are the misfolding and aggregation of Aβ peptides, including Aβ40 and the more aggregation-prone Aβ42, which originates from the abnormal processing of amyloid precursor protein (APP) by secretase enzymes (Karran and De Strooper, 2022; Selkoe and Hardy, 2016). Although Aβ42 primarily constitutes the core of the parenchymal plaques, it is the smaller, soluble oligomeric species of Aβ42 that are considered more toxic and are critical in initiating downstream neurodegenerative cascades leading to neurodegeneration and cognitive decline (Gu and Guo, 2013; Sengupta et al., 2016). In contrast, Aβ40, which is more abundant in the brain, predominantly deposits in the cerebral vasculature and is a key contributor to the development of cerebral amyloid angiopathy (CAA) in AD (Irizarry et al., 2021). Current therapeutic approaches for AD are largely limited to symptomatic relief and do not treat the underlying disease pathology (Zhang et al., 2024). As a result, there is growing emphasis on disease-modifying therapies (DMTs), particularly those targeting the early stages of AD (Kamatham et al., 2024; Sharma et al., 2025). Among these, Aβ-targeting monoclonal antibodies (mAbs) such as lecanemab (Leqembi®, 2023) and donanemab (Kisunla®, 2024) have emerged as promising candidates to treat AD. These agents have revived interest in developing anti-amyloid strategies to treat AD (Cummings, 2023; Kim et al., 2025; Rabinovici et al., 2025). However, their modest therapeutic benefits, limited blood-brain barrier (BBB) penetration, high cost, serious adverse effects and the need for intravenous administration have raised concerns regarding their long-term utility and accessibility (Shi et al., 2022; van Dyck, 2018).

**FIGURE 1:**
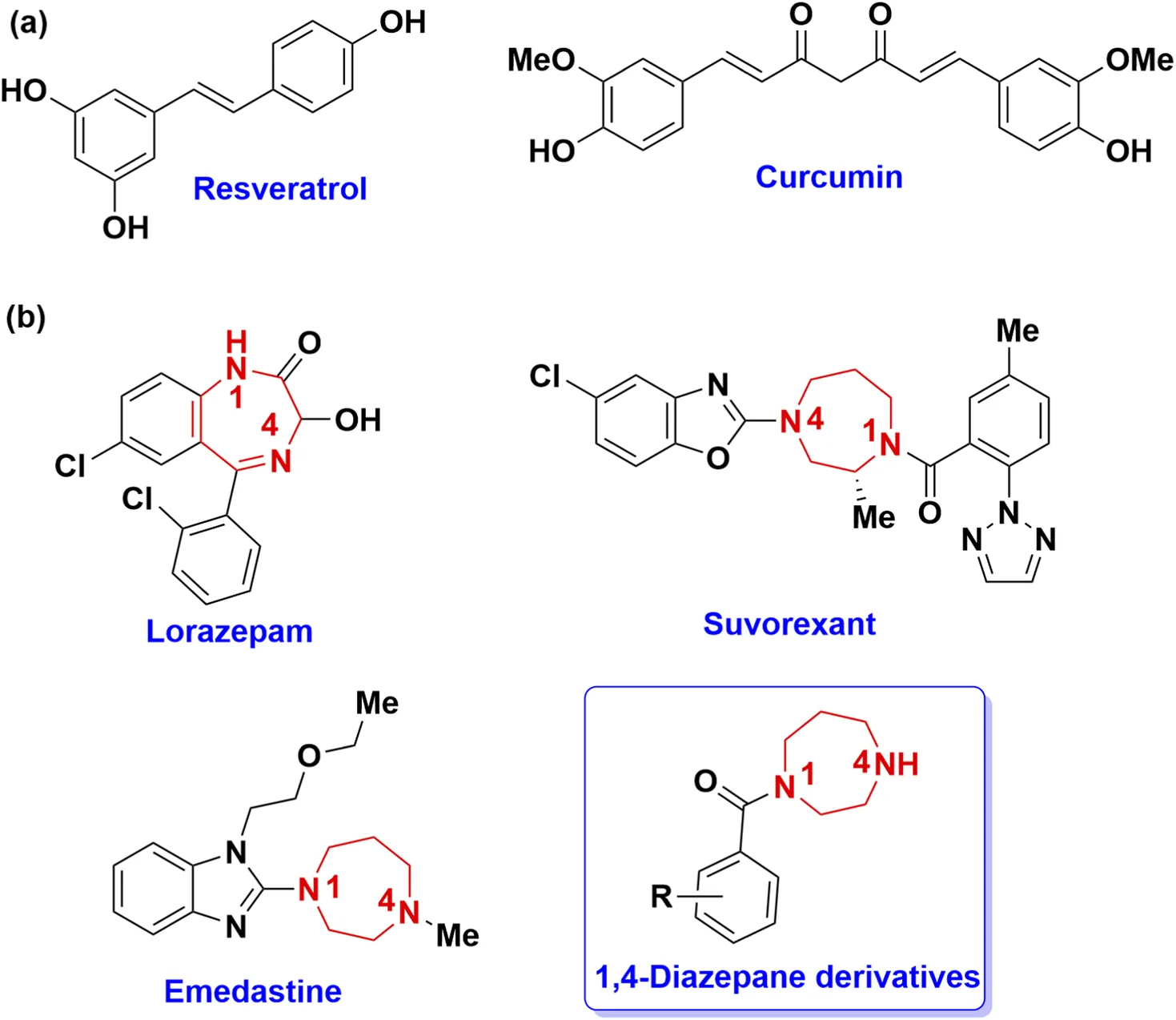
Structures of anti-amyloid natural products and some clinically approved diazepane-containing drugs, and the proposed 1,4-diazepane derivatives. **(a)** Chemical structures of small molecules with anti-Aß activity, **(b)** Chemical structures of approved drugs and the molecular template proposed in this study, incorporating a 1,4-diazepane ring scaffold.

These limitations have prompted a growing interest in developing small molecule drugs capable of targeting the pathogenic events in AD as a safer, better and economical option to treat AD (Zhang et al., 2024). Natural polyphenols such as curcumin and resveratrol (RVT) remain attractive models for designing novel small molecules due to their ability to modulate Aβ aggregation and oxidative stress in AD (Figure 1a) (Ji and Zhang, 2008; Mazzanti and Di Giacomo, 2016). These compounds often contain aromatic rings and hydrogen-bonding ability that facilitate interaction with β-sheet-rich Aβ assemblies. However, their clinical translation is limited by poor solubility, metabolic instability, and structural liabilities (Nelson et al., 2017; Siripruekpong et al., 2024). These limitations underscore the need to develop novel ring scaffolds that retain bioactivity while exhibiting enhanced stability, brain penetration, and pharmacokinetic properties suitable for treating AD. Within this context, seven-membered nitrogen-containing heterocyclic scaffolds (Smith et al., 2014; Yet, 2018), particularly 1,4-diazepanes, represent attractive molecular frameworks due to their conformational flexibility, which enables them to adapt to and interact with the diverse binding surfaces present within Aβ42 and Aβ40 aggregates. Furthermore, the 1,4-diazepane ring can be readily functionalized with aromatic and hydrogen bond donating substituents, allowing the rational optimization of both polar and nonpolar interactions with amyloidogenic regions of Aβ peptides. These saturated 1,4-diazepanes offer enhanced conformational adaptability attributed to the presence of sp^3^-hybridized atoms, enabling the adoption of diverse molecular geometries favourable for drug design (Figure 1b). Our preliminary molecular modeling studies support this design strategy, demonstrating favorable interactions between functionalized 1,4-diazepane derivatives and key binding regions within Aβ42 and Aβ40 aggregates suggesting 1,4-diazepanes as promising scaffolds for the development of novel anti-amyloid agents. Notably, the 1,4-diazepane framework is found in clinically approved agents such as lorazepam (Ativan®, Figure 1b), suvorexant (Belsomra®, Figure 1b), a dual orexin receptor antagonist indicated for insomnia, as well as in emedastine (Emadine®, Figure 1b), an antihistamine drug (Gumieniczek et al., 2021; Smith et al., 2014; Uslaner et al., 2020; Yet, 2018) demonstrating the application of these ring systems in therapy.

Our previous work demonstrated that 1,4-diazepane derivatives functionalized at both the N1 and N4 positions of the diazepane ring with aromatic moieties connected through amide bonds can inhibit Aβ aggregation and mitigate cytotoxicity in HT22 hippocampal neuronal cells (Karuturi et al., 2026). As an extension of this study, we now report the design and synthesis of a new library of (1,4-diazepan-1-yl) (phenyl)methanone derivatives (**4a–n**). In this series, the compounds were selectively functionalized at the N1 position of the diazepane ring with diverse aromatic substituents linked via an amide bond, while retaining a free secondary amine at N4 (Figure 1b). The inhibitory activity of these compounds on Aβ aggregation was evaluated using the thioflavin T (ThT) fluorescence kinetic assays and transmission electron microscopy (TEM). Additionally, antioxidant activity, cell viability assays, and BBB permeability studies were performed to assess their permeability and reduce Aβ42 and hydrogen peroxide-induced cytotoxicity in mouse hippocampal HT22 cells. Computational modelling was conducted to determine the binding interactions of these 1,4-diazepane derivatives with Aβ42 and Aβ40 aggregates. Collectively, our results demonstrate that the (1,4-diazepan-1-yl) (phenyl)methanone derivatives possess anti-Aβ activity, along with neuroprotective and antioxidant properties, demonstrating the application of the novel seven-membered 1,4-diazepane scaffold to design dual inhibitors of both Aβ42 and Aβ40 aggregation.

## 2 Results and discussion

### 2.1 Chemistry

**SCHEME 1:**
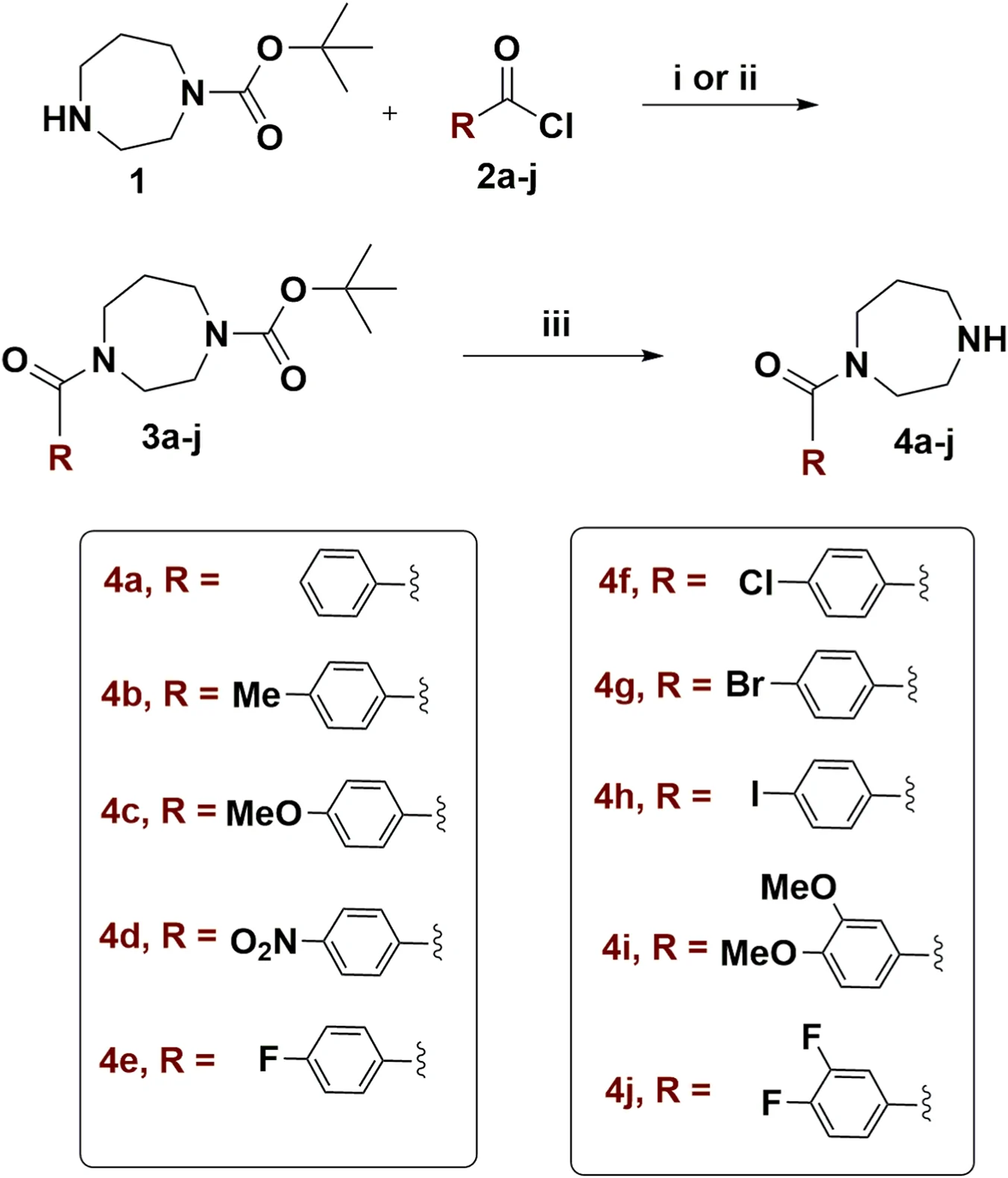
Synthetic route to prepare 1,4-diazepane derivatives (**4a–j**). *Reagents and conditions*. (i) K_2_CO_3_, ACN, rt, 12 h (ii) NaH, DMF, rt, 2 h (iii) TFA, DCM, rt, 2 h.

**SCHEME 2:**
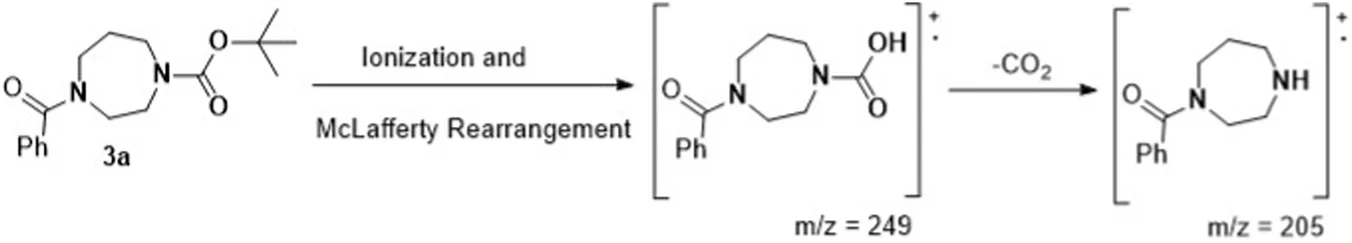
Mass spectral fragmentation of **3a** after ionization and McLafferty rearrangement.

The (1,4-diazepan-1-yl) (phenyl)methanone derivatives (**4a–j**) were synthesized by acylation of *tert*-butoxycarbonyl (*t*-Boc)-protected 1,4-diazepane (**1**) using various substituted benzoyl chlorides (**2a–j**) (Fones, 1949; Zampieri et al., 2020). The reaction was carried out in acetonitrile (ACN) with potassium carbonate or sodium hydride (NaH) as a base in DMF at r. t., affording *t*-Boc-protected intermediates (**3a–j**), with yields ranging from 10% to 68% as outlined in Scheme 1. It should be noted that the *t*-Boc-protected intermediates (**3a–j**) were unstable under the mass spectrometry conditions as the *tert*-butyl carbamoyl group undergoes McLafferty rearrangement after ionization to form the corresponding carbamoyl acid (Wolf et al., 2005) which further undergoes decarboxylation to form the corresponding amine as shown in Scheme 2 (mass spectral details are provided in the Supporting Information). These intermediates were then deprotected using trifluoroacetic acid (TFA) to yield the final products **4a–j** with yields ranging from 12% to 63% (Scheme 1; Takaishi et al., 2018).

**SCHEME 3:**
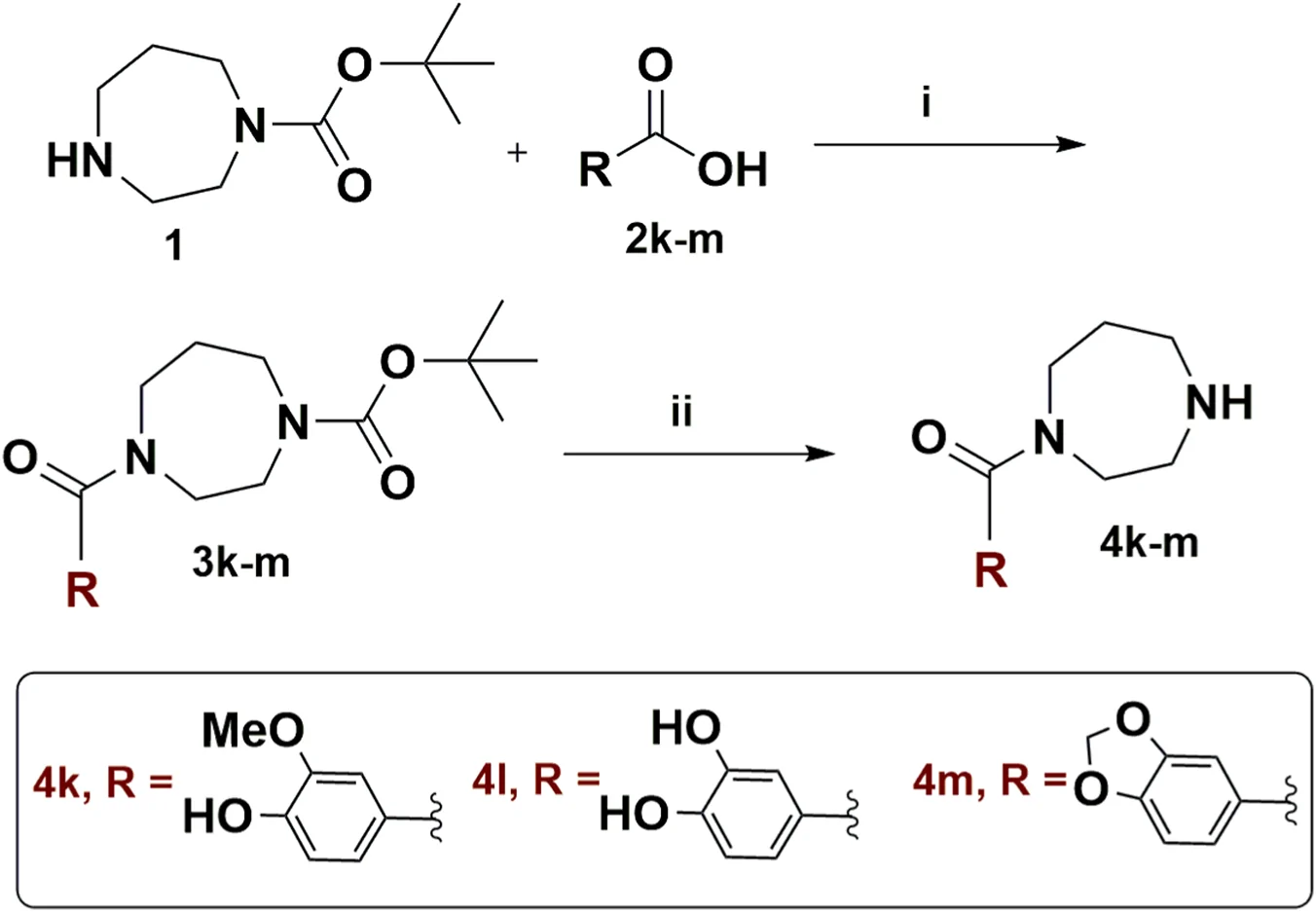
Synthetic route to prepare 1,4-diazepane derivatives (**4k–m**). *Reagents and conditions*. (i) EDC•HCl, HOBt, TEA, THF, rt, or reflux, 12 h (ii) TFA, DCM, rt, 2 h.

The (1,4-diazepan-1-yl) (phenyl)methanone derivatives (**4k–m**) were synthesized by coupling Boc-protected 1,4-diazepane (**1**) with corresponding carboxylic acids (**2k–m**) using EDC•HCl and HOBt in the presence of triethylamine (TEA) in tetrahydrofuran (THF) to obtain compounds **3k–m**, as depicted in Scheme 3 (Chan and Cox, 2007). The desired intermediates were obtained in yields ranging from 27% to 74%. Subsequent TFA-mediated deprotection afforded the target derivatives **4k–m** with yields ranging from 27% to 47% (Scheme 3).

**SCHEME 4:**
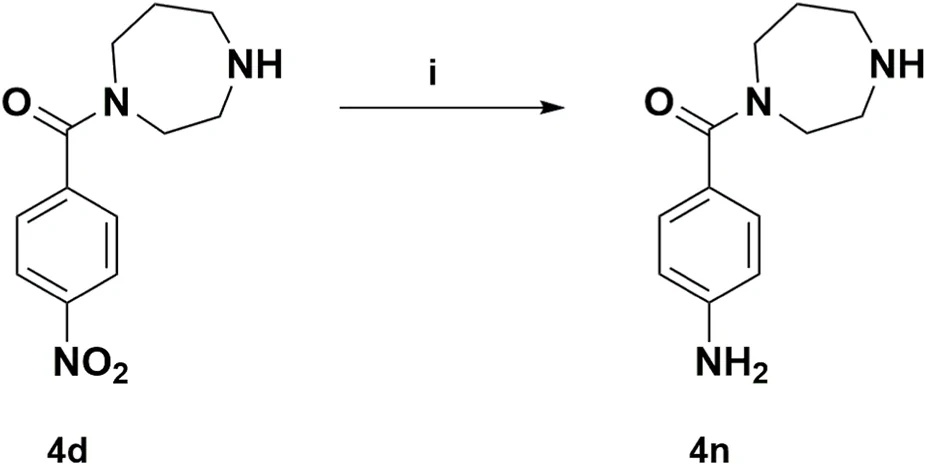
Preparation of (1,4-diazepan-1-yl) (phenyl)methanone derivative (**4n**). *Reagents and conditions*. (i) Pd/C, hydrazine hydrate, EtOH, reflux, 2 h.

The amine-substituted compound **4n** was synthesized via catalytic reduction of the nitro-substituted derivative (**4d**), using hydrazine hydrate and palladium on carbon (Pd/C), affording the corresponding amine in 89% yield (Scheme 4; Qiu et al., 2019). The analytical characterization data for all the final compounds (**4a–n**) are provided in the Supporting Information, SI.

### 2.2 Biological evaluation

#### 2.2.1 Inhibition of amyloid-β aggregation

**FIGURE 2:**
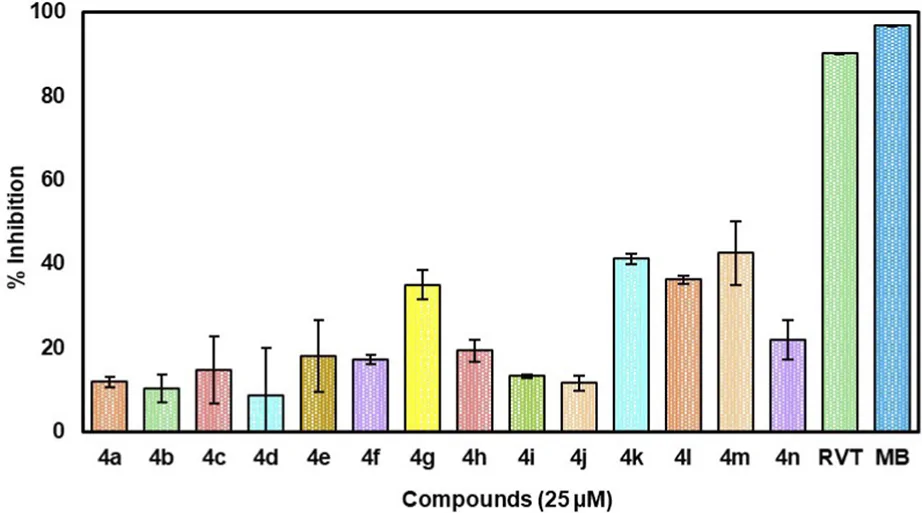
ThT fluorescence-based assessment of Aβ42 aggregation inhibition by (1,4-diazepan-1-yl) (phenyl)methanone derivatives (**4a–n**), RVT, and MB (25 µM each). Assay was conducted with Aβ42 (10 µM) at pH 7.4, 37 °C for 24 h. Results represent the average of duplicate readings from two independent experiments.

The anti-aggregation potential of a series of fourteen (1,4-diazepan-1-yl) (phenyl)methanone derivatives (**4a–n**) toward both Aβ42 and Aβ40 was evaluated using a thioflavin T (ThT)-based fluorescence aggregation kinetics assay (Karuturi et al., 2026; Mohamed et al., 2016). Initially, the 1,4-diazepane derivatives were evaluated at 25 µM to determine their anti-aggregation activity toward Aβ42 (10 µM) after incubating for 24 h at 37 °C. Known Aβ aggregation inhibitors, resveratrol (RVT) and methylene blue (MB), were used as reference agents. The structure-activity relationship (SAR) analysis revealed inhibitory activity ranging from 9% to 43% (Figure 2). The unsubstituted phenyl derivative **4a** exhibited weak inhibition (12%), while derivatives bearing electron-donating substituents on the aromatic ring, including 4-Me (**4b**), 4-OMe (**4c**), 3,4-diOMe (**4i**), and 4-NH_2_ (**4n**), showed an increase in activity ranging from 10.2% to 21.8% (Figure 2). Notably, modification of compound **4i** to compound **4k** (4-hydroxy-3-methoxyphenyl) and **4l** (3,4-dihydroxyphenyl) led to a significant increase in the inhibition of Aβ42 aggregation (41% and 36%, respectively). In contrast, derivatives bearing electron-withdrawing groups, such as 4-NO_2_ (**4d**), 4-F (**4e**), 4-Cl (**4f**), 4-I (**4h**), and 3,4-diF (**4j**), demonstrated weak inhibition (8.8%–19.4%). Interestingly, the 4-bromo derivative, **4g**, deviated from this trend, displaying superior inhibition of Aβ42 (35%) compared to other 1,4-diazepane derivatives possessing electron-withdrawing groups (Figure 2). Additionally, replacing the 3,4-dihydroxyphenyl ring in compound **4l** with a masked catechol moiety (R = benzo[*d*][1,3]dioxole) in compound **4m** led to superior inhibition (~43%).

**FIGURE 3:**
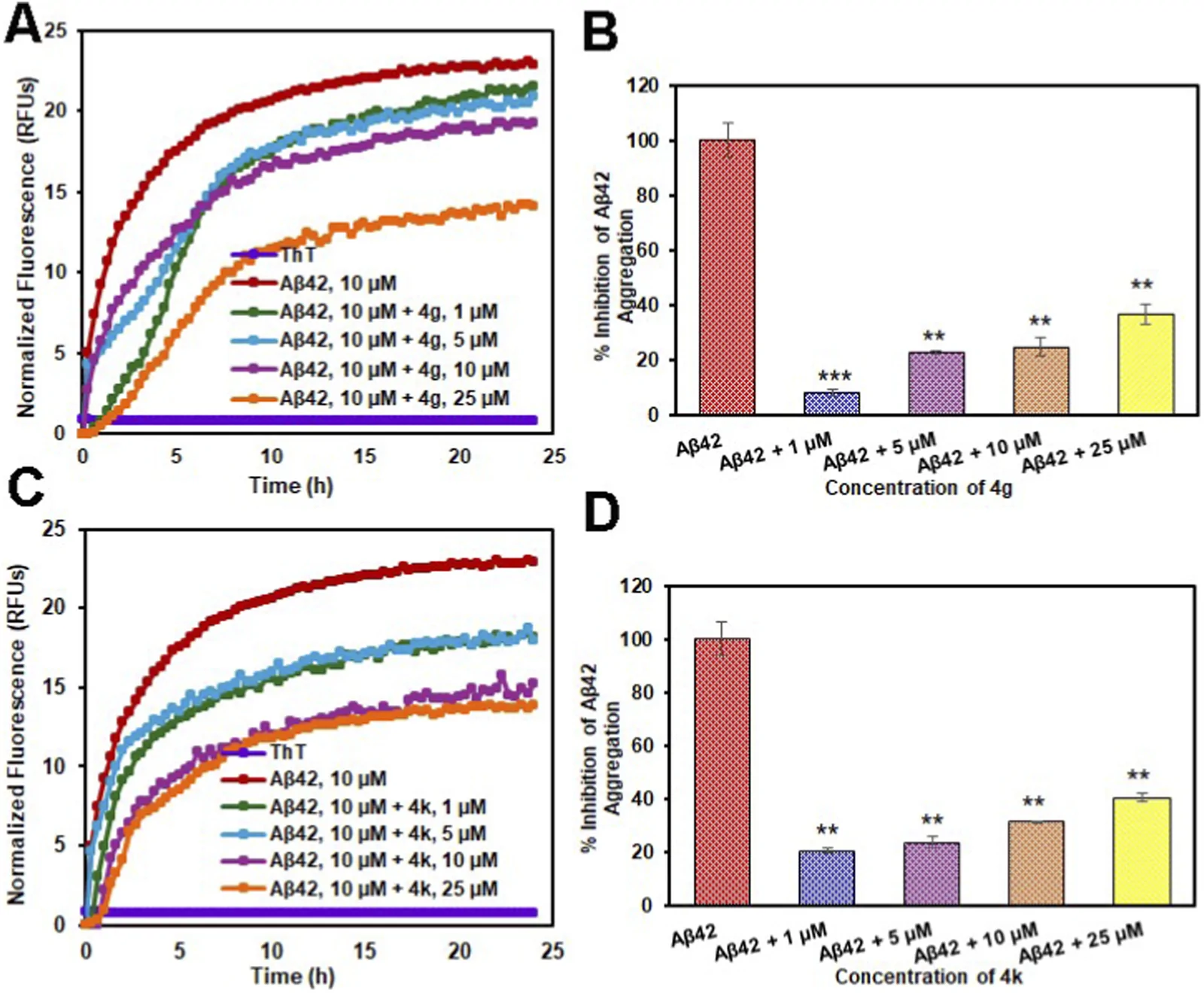
ThT fluorescence-based monitoring of Aβ42 aggregation kinetics in the presence of compound **4g (A,B)** and **4k (C,D)** at 1, 5, 10, and 25 μM, respectively. The aggregation assays were performed using Aβ42 (10 µM) at pH 7.4, 37 °C for 24 h. ThT fluorescence was recorded (excitation at 440 nm and emission at 490 nm), and the results are presented as average ± SD from three independent experiments (n = 3). **p < 0.01 and ***p < 0.001 for compound treated groups compared to Aβ40 alone treated group. One-way ANOVA followed by Bonferroni *post hoc* test.

**FIGURE 4:**
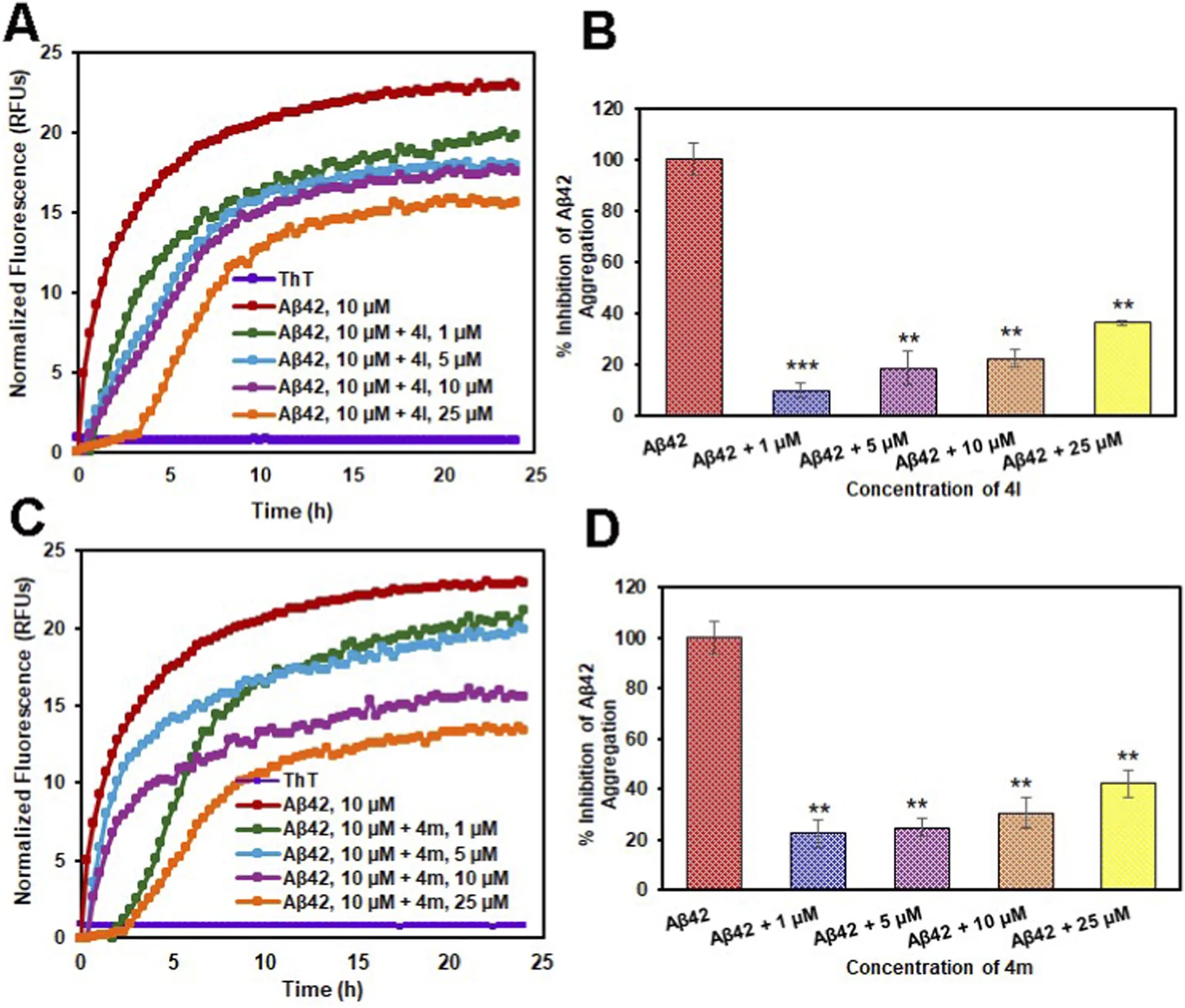
ThT fluorescence-based monitoring of Aβ42 aggregation kinetics in the presence of compound **4l (A,B)** and **4m (C,D)** at 1, 5, 10, and 25 μM, respectively. The aggregation assays were performed using Aβ42 (10 µM) at pH 7.4, 37 °C for 24 h. ThT fluorescence was recorded (excitation at 440 nm and emission at 490 nm), and the results are presented as average ± SD from three independent experiments (n = 3). **p < 0.01 and ***p < 0.001 for compound treated groups compared to Aβ42 alone treated group. One-way ANOVA followed by Bonferroni *post hoc* test.

Based on the initial screening studies, compounds **4g** (R = 4-bromophenyl), **4k** (R = 4-hydroxy-3-methoxyphenyl), **4l** (R = 3,4-dihydroxyphenyl), and compound **4m** (R = benzo[*d*][1,3]dioxole) that exhibited anti-Aβ42 activity ranging from 35% to 43% were subsequently subjected to a concentration-dependent study at 1, 5, 10, and 25 µM to investigate their effects on Aβ42 aggregation kinetics during the 24 h study. The Aβ42 aggregation kinetics data revealed that compounds **4g**, **4k**, **4l**, and **4m** exhibited a concentration-dependent inhibition of Aβ42 aggregation, with percentage inhibition ranging from 8% to 42% over a 24 h period (Table 1; Figures 3, 4). In the absence of test compounds, Aβ42 aggregation followed the characteristic kinetic profile, as evidenced by an increase in thioflavin T (ThT) fluorescence intensity, with a rapid growth phase followed by a plateau (Figure 3). In contrast, co-incubation with compounds **4g**, **4k**, **4l**, and **4m** resulted in a marked reduction in ThT fluorescence intensity, indicating reductions in Aβ42 fibril formation (Figures 3A–D; Figures 4A–D). Among these, compounds **4k** (R = 4-hydroxy-3-methoxyphenyl) and **4m** (R = benzo[*d*][1,3]dioxole) displayed the most pronounced activity, with inhibition of 40% and 42%, respectively, at 25 µM (Figures 3C,D, 4C,D respectively; Table 1). Compounds **4g** (R = 4-bromophenyl) and **4l** (R = 3,4-dihydroxyphenyl) demonstrated similar concentration-dependent trends, exhibiting inhibition ranging from 8% to 36% across the 1–25 µM range (Figures 3A,B, 4A,B respectively; Table 1). Notably, structural modification of compound **4l**, through masking the catechol moiety with a fused dioxole ring, in compound **4m** (R = benzo[*d*][1,3]dioxole), enhanced the inhibitory activity to 42% at 25 µM (Figure 4D). These studies demonstrate that the novel (1,4-diazepan-1-yl) (phenyl)methanone derivatives possessing a flexible 1,4-diazepane ring scaffold can be modified by SAR to obtain Aβ42 aggregation inhibitors. It should be noted that these compounds were less potent inhibitors compared to reference agents RVT and MB (Table 1). These findings demonstrate that the novel 1,4-diazepane scaffold is a promising template for the design of Aβ42 aggregation inhibitors. Furthermore, the incorporation of phenolic rings (eg: compounds **4k** and **4l**) and bicyclic benzo[*d*][1,3]dioxole rings (**4m**) appears to enhance anti-aggregation potency, offering a potential SAR avenue for further optimization.

**TABLE 1:** The anti-Aβ42 aggregation activity of (1,4-diazepan-1-yl) (phenyl)methanone derivatives **4g** and **4k–m**.

Compd	R	% Inhibition for Aβ42^a^				ClogP^b^
		1 µM	5 µM	10 µM	25 µM	
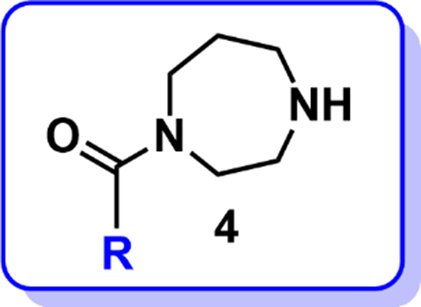						
**4g**	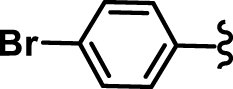	8.0 ± 1.0	22.5 ± 0.5	24.6 ± 3.4	36.4 ± 3.6	1.57
**4k**	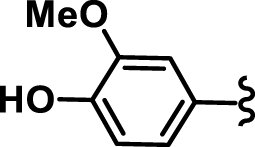	20.3 ± 1.1	23.4 ± 2.3	31.1 ± 0.4	40.4 ± 1.3	0.32
**4l**	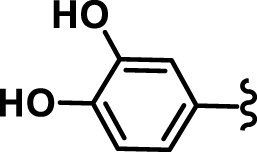	9.8 ± 2.7	18.5 ± 6.7	22.3 ± 3.3	36.1 ± 0.7	0.07
**4m**	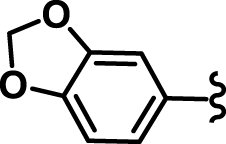	22.4 ± 5.4	24.7 ± 3.6	30.3 ± 6.0	42.1 ± 5.4	0.83
**RVT**	​	44.5 ± 11.2	68.6 ± 4.5	82.0 ± 2.2	89.7 ± 2.3	2.83
**MB**	​	80.3 ± 2.2	94.9 ± 1.0	96.6 ± 1.2	97.9 ± 0.4	0.94

^a^ Inhibition of Aβ42 (10 µM) aggregation by 1,4-diazepane derivatives assessed via ThT-based fluorescence kinetics assay (excitation at 440 nm and emission at 490 nm) over 24 h, at 37 °C and pH 7.4. The results are expressed as average ± SD, from three independent experiments (n = 3).

^b^ ClogP values were calculated using ChemDraw 23.1.2.

To further assess the anti-aggregation potential of the (1,4-diazepan-1-yl) (phenyl)methanone series, we examined compounds **4a–n** for their ability to inhibit Aβ40 fibrillization using the ThT-based fluorescence assay (Karuturi et al., 2026; Mohamed et al., 2016). In the ThT assay screening at 25 μM, the 1,4-diazepane derivatives demonstrated anti-aggregation activities ranging from 13% to 64.7% (Supplementary Figure S1). In general, several compounds from this series exhibited good inhibition of Aβ40 aggregation. For example, the parent compound **4a** (R = phenyl) showed substantial inhibition of Aβ40 (55%) at 25 µM. Incorporation of the 4-hydroxy-3-methoxyphenyl ring in compound **4k** provided 56% inhibition, similar to compound **4a**. Interestingly, replacing the 3-methoxy group in **4k** with an additional hydroxyl group in **4l** (R = 3,4-dihydroxyphenyl/catechol ring) led to significant inhibition of Aβ40 aggregation, with **4l** achieving the highest activity in the series with 64.7% inhibition (Supplementary Figure S1). In contrast, other electron-donating groups, including 4-methyl (**4b**), 4-methoxy (**4c**), 3,4-dimethoxy (**4i**), and 4-NH_2_ (**4n**) phenyl substituents, resulted in low-to-moderate inhibition, with activities ranging from 13% to 35.3%. Masking the catechol moiety in **4l** with a fused dioxole ring in compound **4m** (R = benzo[*d*][1,3]dioxole) led to a notable decrease in activity (34% inhibition, Supplementary Figure S1). Compounds bearing electron-withdrawing groups (4-nitro, 4-F, 4-Cl, 4-Br, 4-I, and 3,4-diF phenyl) in compounds **4d**, **4e**, **4f**, **4g**, **4h**, and **4j** also exhibited low-to-moderate activity from 14.8% to 41.3%, comparable to that observed for some electron-donating derivatives (Supplementary Figure S1).

**FIGURE 5:**
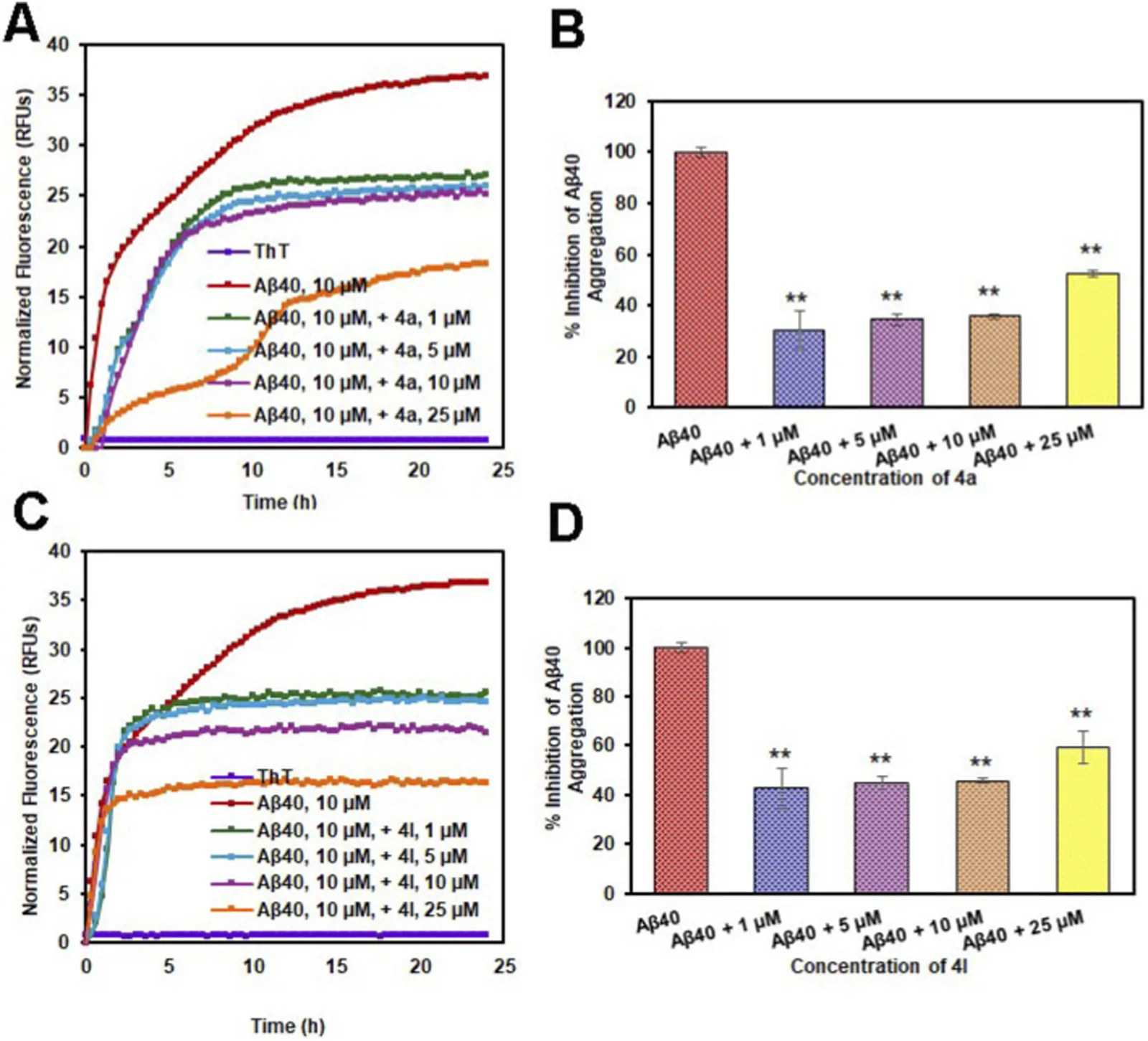
ThT fluorescence-based monitoring of Aβ40 aggregation kinetics in the presence of compound **4a (A, B)** and compound **4l (C, D)** at 1, 5, 10, and 25 μM, respectively. The aggregation assays were performed using Aβ40 (10 µM) at pH 7.4, 37 °C for 24 h. ThT fluorescence was recorded (excitation at 440 nm and emission at 490 nm), and the results are presented as average ± SD from three independent experiments (n = 3). **p < 0.01 for compound treated groups compared to Aβ40 alone treated group. One-way ANOVA followed by Bonferroni *post hoc* test.

The initial studies identified compounds **4a**, **4k**, and **4l** as promising candidates and further evaluated their concentration-dependent effects on Aβ40 aggregation kinetics at 1, 5, 10, and 25 µM. Compound **4a** (R = phenyl) demonstrated consistent activity across 1, 5 and 10 µM (30%–36% inhibition, Table 2), with enhanced activity observed at 25 µM (∼53% inhibition, Table 2; Figures 5A,B). In contrast, compounds **4l** (R = 3,4-dihydroxyphenyl) and **4k** (R = 4-hydroxy-3-methoxyphenyl), possessing antioxidant moieties, showed superior inhibition at the higher concentration tested, reaching 59% and 57%, respectively (Figures 5C,D; Supplementary Figure S2). Nonetheless, these compounds were not as potent as the reference agents, RVT and MB (∼87% and ∼98% inhibition, respectively, Table 2). These results identified compound **4a** as a promising inhibitor of Aβ40 aggregation, with **4k** and **4l** emerging as dual inhibitors of both Aβ42 and Aβ40 aggregation.

**TABLE 2:** The anti-Aβ40 aggregation activity of (1,4-diazepan-1-yl) (phenyl)methanone derivatives **4a**, **4k**, and **4l**.

Compd	R	% Inhibition for Aβ40^a^				ClogP^b^
		1 µM	5 µM	10 µM	25 µM	
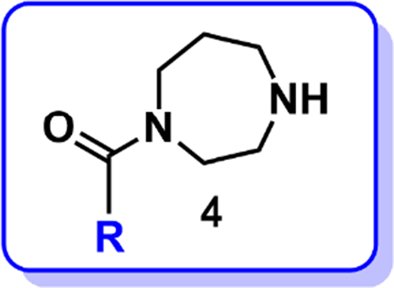						
**4a**	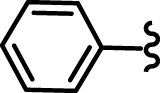	30.4 ± 7.4	34.5 ± 2.2	35.9 ± 0.9	52.7 ± 1.2	0.63
**4k**	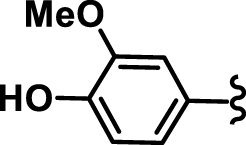	30.2 ± 4.3	34.6 ± 5.6	44.7 ± 5.2	57.3 ± 5.8	0.32
**4l**	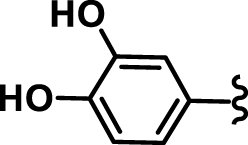	42.8 ± 8.2	44.8 ± 2.4	45.7 ± 1.0	59.2 ± 6.6	0.07
**RVT**	​	47.5 ± 2.7	65.2 ± 6.3	74.2 ± 6.3	86.5 ± 5.6	2.83
**MB**	​	87.6 ± 8.3	91.1 ± 7.6	96.9 ± 0.8	97.9 ± 0.7	0.94

^a^ Inhibition of Aβ40 (10 µM) aggregation by 1,4-diazepane derivatives assessed via ThT-based fluorescence kinetics assay (excitation at 440 nm and emission at 490 nm) over 24 h at 37 °C and pH 7.4. The results are expressed as average ± SD, from three independent experiments (n = 3).

^b^ ClogP values were calculated using ChemDraw 23.1.2.

It should be noted that although Aβ42 is widely considered the principal pathogenic species driving amyloid plaque formation in AD due to its higher hydrophobicity, aggregation propensity, and neurotoxicity, Aβ40 also contributes significantly to disease pathology and is the predominant amyloid-β species in the cerebrospinal fluid and brain. It also plays an important role in cerebral amyloid angiopathy (CAA), a condition present in more than 80% of AD patients, characterized by the deposition of amyloid aggregates within cerebral blood vessel walls (Greenberg et al., 2020; Irizarry et al., 2021). Therefore, both Aβ42 and Aβ40 are implicated in AD pathology, albeit through distinct but complementary mechanisms. In this regard our studies demonstrate that 1,4-diazepanes have the potential to exhibit dual inhibition of both Aβ42 and Aβ40 aggregation.

#### 2.2.2 Transmission electron microscopy studies

**FIGURE 6:**
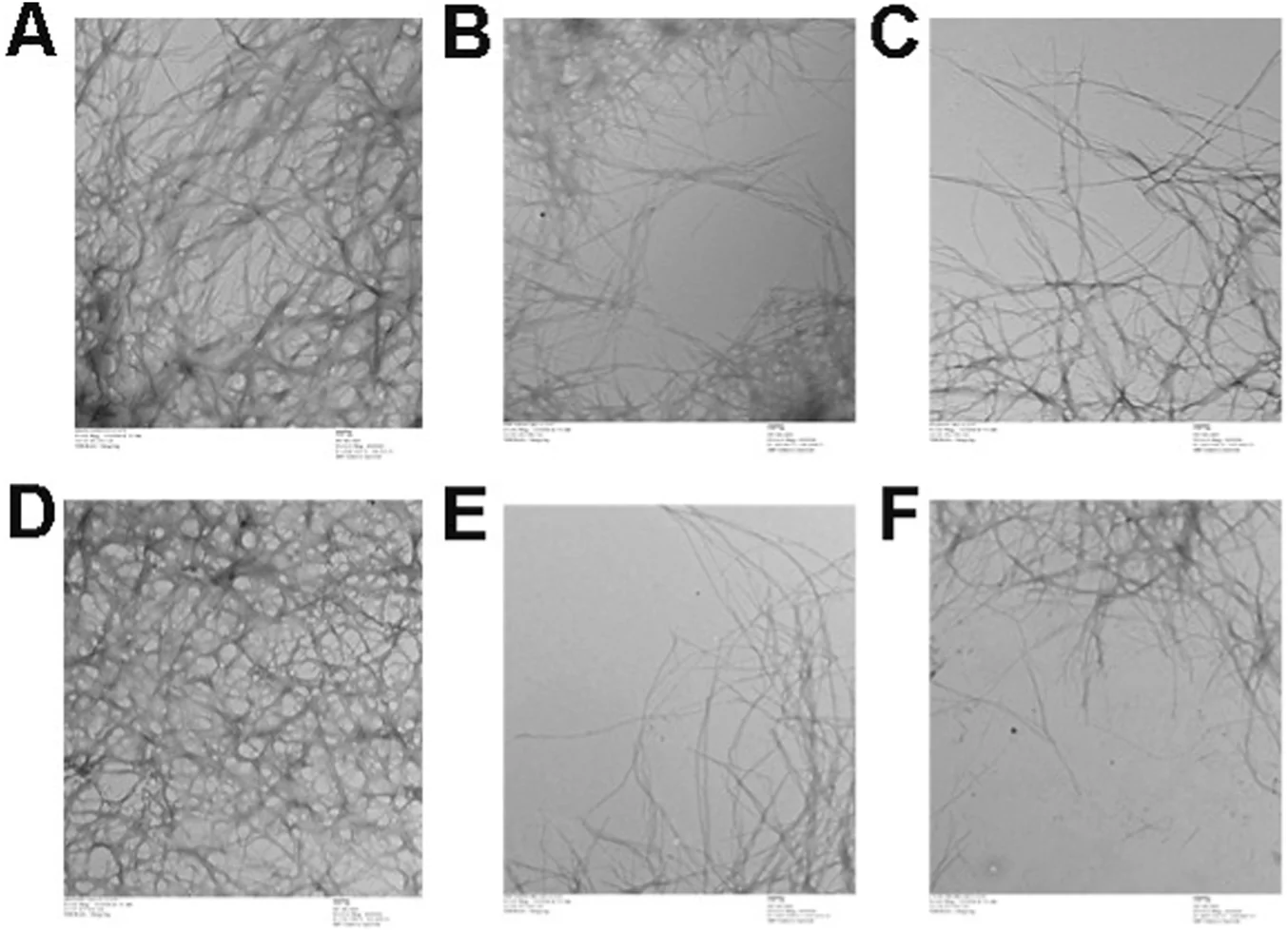
TEM images of Aβ42 (10 µM) in the presence and absence of 1,4-diazepane derivatives (25 µM). **(A)** Aβ42 control. **(B)** Aβ42 in the presence **4k**. **(C)** Aβ42 in the presence **4m**. **(D)** Aβ40 control. **(E)** Aβ40 in the presence **4k**. **(F)** Aβ40 in the presence **4l**. Scale: 100 nm.

The anti-aggregation potential of the representative (1,4-diazepan-1-yl) (phenyl)methanone derivatives, initially assessed by the ThT fluorescence assay, was further corroborated by transmission electron microscopy (TEM) to evaluate the morphological characteristics of Aβ42 and Aβ40 aggregates in the presence of selected test compounds as shown in Figure 6 (Mohamed et al., 2016). TEM analysis revealed that treatment with compounds **4k** and **4m** at 25 µM resulted in a marked reduction in Aβ42 fibril formation (Figures 6B,C) compared to untreated Aβ42, which exhibited dense, elongated, and mature fibrillar structures (Figure 6A). In parallel, treatment of Aβ40 with compounds **4k** and **4l** at 25 µM similarly led to a significant reduction in fibrillar aggregates (Figures 6E,F) relative to the untreated control (Figure 6D). These findings provide further evidence supporting the anti-aggregation activity of (1,4-diazepan-1-yl) (phenyl)methanone derivatives **4k**, **4l**, and **4m** toward Aβ aggregation.

#### 2.2.3 Effects of compounds 4g and 4k–m on Aβ42-Induced cytotoxicity

**FIGURE 7:**
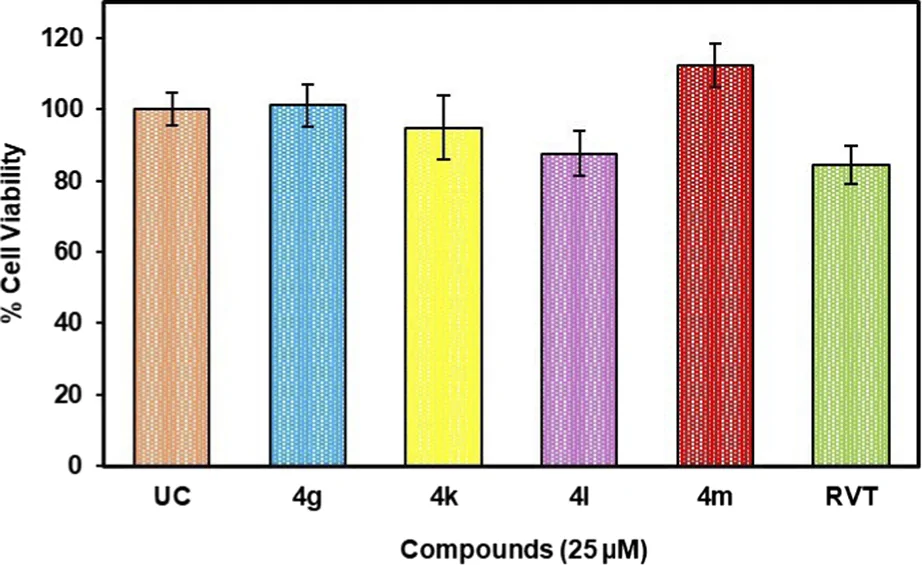
Cell viability of compounds **4g**, **4k**, **4l**, and **4m** (25 µM) in HT22 mouse hippocampal neuronal cells following 48 h incubation in the absence of Aβ42. The results are presented as the mean ± SD from quadruplicate readings (n = 4) based on two independent experiments.

In order to determine the neuroprotective activity of (1,4-diazepan-1-yl) (phenyl)methanone derivatives **4g**, **4k**, **4l**, and **4m**, we investigated their ability to prevent Aβ42-induced cytotoxicity in mouse hippocampal HT22 neuronal cells. The cytotoxicity of each compound alone was tested at 25 µM in HT22 cells using the Cell Counting Kit-8 (CCK-8) assay (Karuturi et al., 2026; Yao et al., 2023). These compounds were not toxic to mouse hippocampal HT22 cells and maintained cell viability ranging from ∼88% to 112% (Figure 7).

**FIGURE 8:**
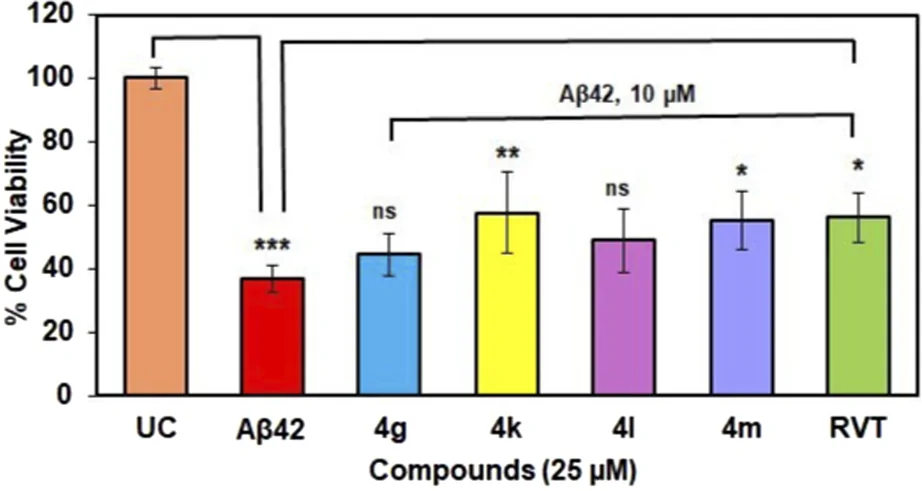
Effects of compounds **4g**, **4k**, **4l**, and **4m** (25 µM) toward Aβ42-induced cytotoxicity in mouse hippocampal HT22 cells after 48 h of co-incubation with Aβ42 (10 µM). The results are presented as the average ± SD of quadruplicate readings from three independent experiments (n = 3). ***p < 0.001 for the Aβ42-alone-treated group compared to the untreated control (UC). *p < 0.05 and **p < 0.01 for compound treated groups compared to Aβ42 alone treated group, ns–not significant. One-way ANOVA followed by Bonferroni *post hoc* test.

Incubation of mouse hippocampal HT22 cells with Aβ42 (10 µM) for 48 h led to a significant decline in cell viability (∼37%, ***p < 0.001, Figure 8). In contrast, co-treatment with 25 µM of either **4g**, **4k**, **4l**, or **4m** resulted in improved cell survival, indicating a protective effect against Aβ42-induced cytotoxicity. Notably, compounds **4k** and **4m** enhanced cell viability to 57% (**p < 0.01) and 55% (*p < 0.05), respectively, which was comparable to the neuroprotection offered by the reference compound RVT (56.1% cell viability, *p < 0.05, Figure 8). Compounds **4g** and **4l** were also able to reduce Aβ42-induced cytotoxicity, increasing cell viability to 44.4% and ∼49%, respectively (Figure 8).

#### 2.2.4 Antioxidant activity

**FIGURE 9:**
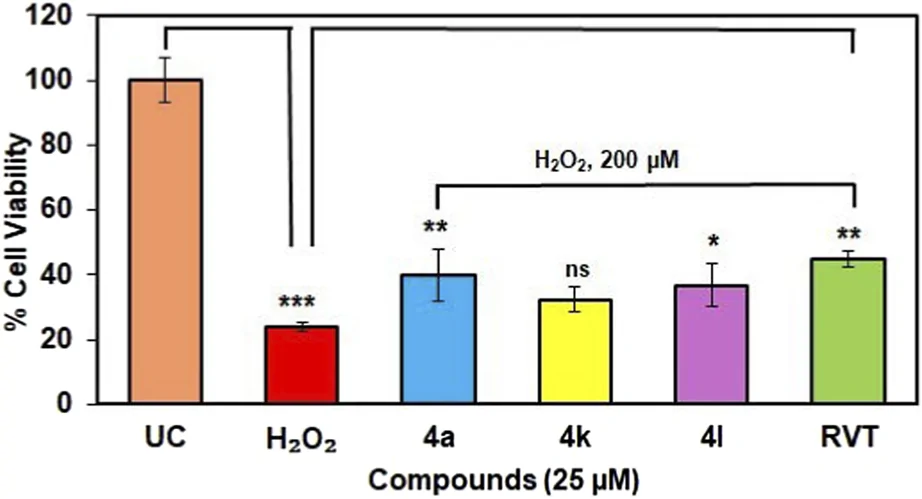
Effects of compounds **4a**, **4k**, **4l**, and RVT (25 µM) on H_2_O_2_-induced cytotoxicity in mouse hippocampal HT22 cells after 24 h of co-incubation with H_2_O_2_ (200 µM). The results are presented as the average ± SD of quadruplicate readings from two independent experiments (n = 2). ***p < 0.001 for the H_2_O_2_-alone-treated group compared to the untreated control (UC). *p < 0.05 and **p < 0.01 for compound treated groups compared to the H_2_O_2_ alone treated group. n. s.- Not significant. One-way ANOVA followed by Bonferroni *post hoc* test.

The antioxidant property of representative 1,4-diazepane derivatives (**4a**, **4k**, and **4l**) was assessed using the DPPH radical (2,2-diphenyl-1-picrylhydrazyl) colorimetric assay. Each compound was evaluated at a concentration of 25 µM following incubation with DPPH for 60 min at 37 °C. Both RVT and trolox were used as reference agents (Kleinrichert and Alappat, 2019). Compounds **4a** (R = phenyl) and **4k** (R = 4-hydroxy-3-methoxyphenyl) demonstrated limited radical scavenging (∼13% inhibition, Supplementary Figure S3). In contrast, compound **4l** (R = 3,4-dihydroxyphenyl), containing a catechol group, displayed significantly enhanced antioxidant capacity (63.2% inhibition), which was comparable to the activity observed for trolox (66.5%, Supplementary Figure S3). Additionally, the antioxidant potential of compounds **4a**, **4k**, and **4l** was evaluated in a cell-based oxidative stress model employing mouse hippocampal HT22 neuronal cells (Hefny et al., 2025). Oxidative stress was induced by treatment with hydrogen peroxide (H_2_O_2_, 200 µM), resulting in substantial cytotoxicity (∼24% cell viability, ***p < 0.001, Figure 9). Co-incubation with test compounds **4a** and **4l** (25 µM) led to significant mitigation of H_2_O_2_-induced toxicity, improving cell viability to 33%–40% (Figure 9). The reference agent, RVT, provided comparatively greater protection, restoring viability to 45% (**p < 0.01) under the same conditions (Figure 9).

#### 2.2.5 Blood-brain barrier (BBB) permeability assay

Furthermore, the blood-brain barrier (BBB) permeability of the lead compounds (**4a**, **4g**, **4k**, **4l**, and **4m**) was evaluated using the parallel artificial membrane permeability assay (PAMPA) with UV spectroscopy (Gujral et al., 2022). Compounds **4a**, **4g**, **4k**, and **4m** demonstrated the ability to permeate the BBB (Supplementary Figure S4; Supplementary Table S1), whereas compound **4l** was not able to exhibit brain permeability. These results also correlated with the ClogP data, with compound **4g** (R = 4-bromophenyl) exhibiting greater BBB permeability compared to other compounds in the series (Supplementary Figure S4; Supplementary Table S1).

#### 2.2.6 Molecular docking

The binding interactions of the (1,4-diazepan-1-yl) (phenyl)methanone derivative **4k** (R = 4-hydroxy-3-methoxyphenyl), which exhibited dual inhibition toward both Aβ42 (40% inhibition) and Aβ40 (57% inhibition), were investigated by conducting computational modelling studies. Molecular docking was performed using the Aβ42 pentamer model (PDB ID: 5KK3) using the LibDock algorithm within the Discovery Studio Structure-Based Design (SBD) software (Colvin et al., 2016). The binding affinity of the most favourable binding mode of compound **4k** in the Aβ42 model was calculated using the equation (Rao et al., 2018):

$$E_{\text{binding}}=\text{Energy of complex }\left(E_{\text{ligand}-\text{recepto}r}\right)-\text{Energy of ligand }\left(E_{\text{ligand}}\right)-\text{Energy of receptor }\left(E_{\text{receptor}}\right)$$

**FIGURE 10:**
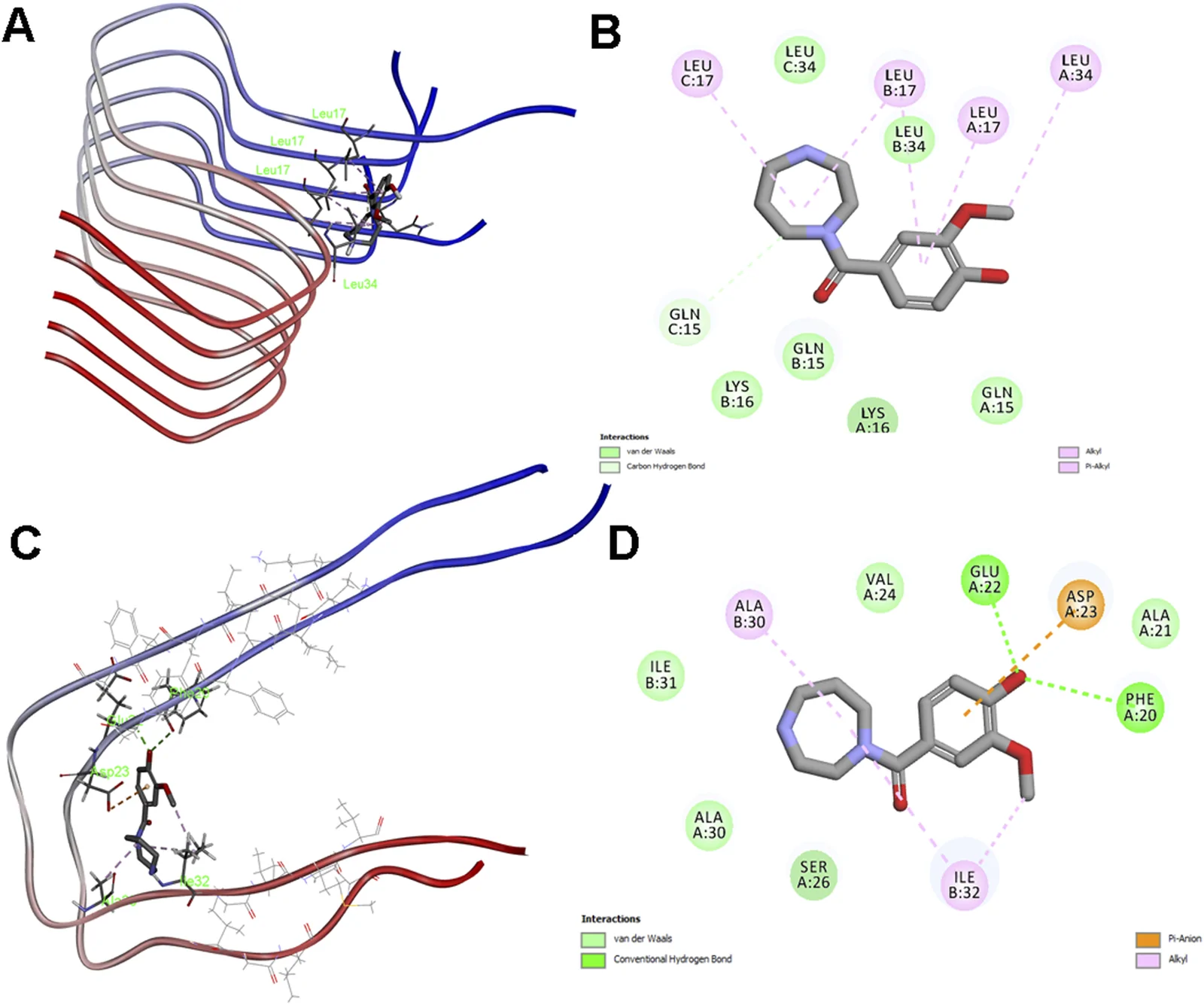
Panel A and B: Molecular docking studies of **4k** (ball and stick) in the Aβ42 pentamer model (PDB ID: 5KK3). **(A)** Top docked pose of **4k** (LibDock score = 70.82). **(B)** Schematic depiction of the 2D binding interactions between **4k** and the Aβ42 pentamer (ribbon diagram); Panel C and D: Molecular docking studies of **4k** (ball and stick) within the Aβ40 dimer model (PDB ID: 2LMN). **(C)** Top docked pose of **4k** (LibDock score = 60.72). **(D)** Schematic depiction of the 2D binding interactions between **4k** and the Aβ40 dimer (ribbon). The N-terminus and C-terminus of the dimer are depicted in blue and red, respectively. For clarity, hydrogen atoms are not shown.

Compound **4k** forms a stable complex, and the binding energy of **4k**–Aβ42 complex was −16.2 kcal/mol. The docking analysis revealed that compound **4k** preferentially oriented within the narrow groove at the interface of the N- and C-termini regions in the Aβ42 pentamer (Figure 10A). The 4-hydroxy-3-methoxyphenyl substituent of **4k** was primarily involved in hydrophobic interactions. Notably, the phenyl ring interacted with Leu17 residues from chains A and B (distance < 5 Å, Figure 10B). The methoxy substituent further contributes through hydrophobic contacts with Leu34 of chain A (< 5 Å). Additionally, the 1,4-diazepane core underwent hydrophobic contacts with Leu17 of chains B and C (< 5 Å). Furthermore, **4k** formed several van der Waals interactions with Gln15 and Lys16 residues across chains A and B, as well as Leu34 in chains B and C (Figure 10B).

Similarly, the molecular docking study of compound **4k** (R = 4-hydroxy-3-methoxyphenyl, 57% inhibition of Aβ40 aggregation) was conducted in the Aβ40 dimer model (PDB ID: 2LMN) (Paravastu et al., 2008). Compound **4k** forms a stable complex with Aβ40, and the binding energy for the **4k**–Aβ40 complex was −6.29 kcal/mol. The results indicate that **4k** interacts with both N- and C-terminal regions of Aβ40, suggesting a role in stabilizing the dimer interface and interfering with further aggregation (Figure 10C). The ligand–protein interaction was driven by a combination of hydrogen bonding, electrostatic, and hydrophobic interactions. Specifically, the free amino group of **4k** formed a hydrogen bond with A:Ala30, while additional hydrogen bonds were observed between the phenolic OH group and residues A:Phe20 and A:Glu22, all within hydrogen-bonding distance (< 3.0 Å, Figure 10D). A π–anion interaction between the aromatic ring and A:Asp23 (distance < 5 Å) was also noted. The central 1,4-diazepane ring engaged in hydrophobic contact with A:Ala30 (3.7 Å), and both the diazepane core (distance < 5 Å), and methoxy substituent (distance < 4 Å) were involved in hydrophobic interactions with B:Ile32. Additional van der Waals contacts were observed with A:Ala21, A:Ile31, A:Ile32, A:Val24, A:Ser26, and B:Ile31, contributing to the overall binding stability (Figure 10D).

## 3 Conclusion

In summary, a focused library of fourteen (1,4-diazepan-1-yl) (phenyl)methanone derivatives (**4a–n**) possessing a flexible 7-membered 1,4-diazepane ring scaffold was synthesized and investigated as a new class of small molecules targeting Aβ aggregation. Among the synthesized compounds, **4k** ((1,4-diazepan-1-yl) (4-hydroxy-3-methoxyphenyl)methanone) and **4l** ((1,4-diazepan-1-yl) (3,4-dihydroxyphenyl)methanone) emerged as promising dual inhibitors, reducing Aβ42 aggregation by 40% and 36%, and excellent inhibition of Aβ40 aggregation (57% and 59%, respectively), while compound **4m** ((benzo[*d*][1,3]dioxol-5-yl (1,4-diazepan-1-yl)methanone)) displayed good inhibition of Aβ42 aggregation (42%). Furthermore, compounds **4k**, **4l**, and **4m** attenuated Aβ42-induced cytotoxicity in mouse hippocampal HT22 neuronal cells. Notably, **4a**, **4k**, and **4m** demonstrated BBB permeability, whereas **4a**, **4k**, and **4l** also exhibited antioxidant activity. Physicochemical assessment via SwissADME (Daina et al., 2017) indicated that **4k** and **4m** comply with Lipinski’s rule of five, supporting their drug-like properties (Supplementary Table S2). Overall, these findings underscore the potential of the flexible 1,4-diazepane ring as a valuable template for developing novel small molecule agents targeting the amyloidogenic pathways in AD.

## 4 Materials and methods

### 4.1 Chemistry

All reagents and solvents were obtained commercially from suppliers, including AA blocks, Sigma-Aldrich, TCI America, Acros Organics, Alfa Aesar, and MP Biomedicals. Compounds with a minimum purity of 95% were used as received, without additional purification. Melting points (mp) were determined using a Fisher-Johns apparatus. Reaction monitoring was carried out via thin-layer chromatography (TLC) on precoated silica gel plates (Merck 60F254) with UV fluorescence. Purification of synthesized compounds was performed by column chromatography using Merck silica gel 60 (230–400 mesh) and appropriate solvent systems. Nuclear magnetic resonance (NMR) spectra, proton (^1^H NMR) and carbon (^13^C NMR), were recorded on Bruker Avance 300 MHz or 600 MHz in either CDCl_3_ or DMSO-*d*_*6*_ at the NMR facility, Department of Chemistry, University of Waterloo. Chemical shifts (δ) were reported in ppm, and coupling constants (*J* values) were given in Hertz (Hz). Splitting patterns are indicated as follows: s (singlet), d (doublet), t (triplet), q (quartet), m (multiplet), and br (broad). Purity and molecular weight confirmation were carried out using liquid chromatography-mass spectrometry (LC-MS) with an Agilent 1,260 Infinity system coupled to a 6,130 single quadrupole mass spectrometer, utilizing a Zorbax Eclipse AAA column (4.6 × 75 mm, 3.5 micron) at a flow rate of 1.0 mL/min. The mobile phase consisted of a 50:50 mixture of 0.1% formic acid (FA) in water and 0.1% FA in acetonitrile (ACN, v/v). Few samples were analyzed using a Waters Acquity UPLC system using an HSS C18 column (2.1 × 50 mm, 1.8 µm), employing a mobile phase consisting of a 10:90 (v/v) mixture of 0.1% trifluoroacetic acid (TFA) in water and 0.1% TFA in ACN. High-resolution mass spectrometry (HRMS) data were acquired on a Q-Exactive Orbitrap mass spectrometer (Thermo Scientific) operated in positive electrospray ionization (ESI) mode, Department of Chemistry, University of Waterloo. All final compounds were confirmed to have a purity of ≥ 95% through LCMS and UPLC analysis.

#### 4.1.1 General procedure for the synthesis of Boc-protected 1,4-diazepane intermediates (3a–j)

*Method i*: To a solution of 1-Boc-homopiperazine **1** (1.0 g, 5.0 mmol, 1.0 mL) in 10 mL of ACN in a 100 mL round-bottom flask (RBF), potassium carbonate (K_2_CO_3_, 1.0 g, 7.2 mmol, 1.5 eq.) was added and stirred at room temperature (r.t.) for 10 min. The corresponding acid chloride derivatives (**2a–j**, 1.0 eq.) were then added gradually, and the reaction mixture was stirred for 12 h at r. t. Then the solvent was removed under reduced pressure (Wang et al., 1999). The residue was diluted with 10 mL brine and extracted with EtOAc (3 × 10 mL). The combined organic extracts were dried over anhydrous MgSO_4_, filtered, and concentrated *in vacuo*. Purification by silica gel column chromatography using suitable solvent systems (5:1 EtOAc:MeOH, 7:1 EtOAc:acetone, or 9:1 DCM:MeOH) afforded the target compounds **3a–j** in 10%–68% yield.

*Method ii*: To a solution of 1-Boc-homopiperazine **1** (0.5 g, 5.0 mmol) in DMF (10 mL), sodium hydride (NaH, 2.5 eq.) was gradually added with stirring in an ice bath for 10 min. The acid chloride **2d** (2.1 eq.) was then added, and the reaction was allowed to proceed at room temperature for 2 h (Fones, 1949). Upon completion, 10 mL of brine was added, and the mixture was extracted three times with EtOAc (3 × 10 mL). The combined organic layers were dried over anhydrous MgSO_4_, filtered, and concentrated under reduced pressure. The crude product was purified by silica gel column chromatography with a 2:1 EtOAc/acetone as the eluent, yielding compound **3d** in 50% yield. ^1^H NMR data for these intermediates are provided below:

##### 4.1.1.1 *tert*-Butyl 4-benzoyl-1,4-diazepane-1-carboxylate (3a)

Yellow oil; Yield: 68% (25); ^1^H NMR (DMSO-*d*_*6*_, 300 MHz): δ 1.40–1.19 (m, 9H), δ 1.53–1.44 (m, 1H), δ 1.79–1.64 (m, 1H), δ 3.28–3.22 (m, 1H), δ 3.41–3.32 (m, 4H), δ 3.50–3.43 (m, 1H), δ 3.60–3.52 (m, 1H), δ 3.70–3.62 (m, 1H), δ 7.34–7.28 (m, 2H), and δ 7.45–7.36 (m, 3H). LRMS (ESI) m/z calcd for C_17_H_25_N_2_O_3_ [M + H]^+^ 305.3, found 305.0.

##### 4.1.1.2 *tert*-Butyl 4-(4-methylbenzoyl)-1,4-diazepane-1-carboxylate (3b)

Yellow oil; Yield: 68%; ^1^H NMR (DMSO-*d*_*6*_, 300 MHz): δ 1.42–1.19 (m, 9H), δ 1.55–1.43 (m, 1H), δ 1.79–1.64 (m, 1H), δ 2.30 (s, 3H), δ 3.29–3.22 (m, 1H), δ 3.41–3.31 (m, 4H), δ 3.49–3.42 (m, 1H), δ 3.58–3.50 (m, 1H), δ 3.69–3.60 (m, 1H), and δ 7.31–7.11 (m, 4H). LRMS (ESI) m/z calcd for C_18_H_27_N_2_O_3_ [M + H]^+^ 319.4, found 319.0.

##### 4.1.1.3 *tert*-Butyl 4-(4-methoxybenzoyl)-1,4-diazepane-1-carboxylate (3c)

Yellow oil; Yield: 26%; ^1^H NMR (DMSO-*d*_*6*_, 300 MHz): δ 1.40–1.19 (m, 9H), δ 1.61–1.44 (m, 1H), δ 1.80–1.62 (m, 1H), δ 3.38–3.31 (m, 4H), δ 3.48–3.38 (m, 2H), δ 3.57–3.49 (m, 1H), δ 3.68–3.58 (m, 1H), δ 3.75 (s, 3H), δ 6.95 (d, *J* = 8.3 Hz, 2H), and δ 7.29 (d, *J* = 7.9 Hz, 2H). LRMS (ESI) m/z calcd for C_18_H_27_N_2_O_4_ [M + H]^+^ 335.4, found 335.1.

##### 4.1.1.4 *tert*-Butyl 4-(4-nitrobenzoyl)-1,4-diazepane-1-carboxylate (3d)

Brown oil; Yield: 50%; ^1^H NMR (DMSO-*d*_*6*_, 300 MHz): δ 1.41–1.23 (m, 9H), δ 1.55–1.45 (m, 1H), δ 1.80–1.69 (m, 1H), δ 3.25–3.20 (m, 1H), δ 3.41–3.32 (m, 4H), δ 3.55–3.44 (m, 1H), δ 3.60 (t, *J* = 5.2 Hz, 1H), δ 3.73–3.64 (m, 1H), δ 7.70–7.52 (m, 2H), and δ 8.34–8.19 (m, 2H). LRMS (ESI) m/z calcd for C_17_H_24_N_3_O_5_ [M + H]^+^ 350.3, found 294.1.

##### 4.1.1.5 *tert*-Butyl 4-(4-fluorobenzoyl)-1,4-diazepane-1-carboxylate (3e)

Yellow oil; Yield: 22%; ^1^H NMR (CDCl_3_, 300 MHz): δ 1.49–1.34 (m, 9H), δ 1.73–1.66 (m, 1H), δ 2.07–1.89 (m, 1H), δ 3.47–3.39 (m, 5H), δ 3.63–3.56 (m, 1H), δ 3.71–3.64 (m, 1H), δ 3.81–3.72 (m, 1H), δ 7.09 (t, *J* = 8.6 Hz, 2H), and δ 7.44–7.33 (m, 2H). LRMS (ESI) m/z calcd for C_17_H_24_FN_2_O_3_ [M + H]^+^ 323.3, found 323.1.

##### 4.1.1.6 *tert*-Butyl 4-(4-chlorobenzoyl)-1,4-diazepane-1-carboxylate (3f)

Yellow oil; Yield: 10%; ^1^H NMR (CDCl_3_, 300 MHz): δ 1.49–1.37 (m, 9H), δ 1.77–1.63 (m, 1H), δ 2.06–1.89 (m, 1H), δ 3.47–3.39 (m, 5H), δ 3.63–3.57 (m, 1H), δ 3.68 (t, *J* = 5.5 Hz, 1H), δ 3.80–3.74 (m, 1H), δ 7.31 (d, *J* = 8.5 Hz, 2H), and δ 7.38 (d, *J* = 8.4 Hz, 2H). LRMS (ESI) m/z calcd for C_17_H_24_ClN_2_O_3_ [M + H]^+^ 339.8, found 339.0.

##### 4.1.1.7 *tert*-Butyl 4-(4-bromobenzoyl)-1,4-diazepane-1-carboxylate (3g)

Yellow oil; Yield: 53%; ^1^H NMR (CDCl_3_, 300 MHz): δ 1.48–1.35 (m, 9H), δ 1.69–1.62 (m, 1H), δ 2.01–1.88 (m, 1H), δ 3.49–3.33 (m, 5H), δ 3.62–3.57 (m, 1H), δ 3.68 (t, *J* = 5.5 Hz, 1H), δ 3.81–3.73 (m, 1H), δ 7.53 (d, *J* = 8.3 Hz, 2H), and δ 7.22 (d, *J* = 8.3 Hz, 2H). LRMS (ESI) m/z calcd for C_17_H_24_BrN_2_O_3_ [M + H]^+^ 383.0, found 383.0.

##### 4.1.1.8 *tert*-Butyl 4-(4-iodobenzoyl)-1,4-diazepane-1-carboxylate (3h)

Yellow oil; Yield: 18%; ^1^H NMR (CDCl_3_, 300 MHz): δ 1.49–1.38 (m, 9H), δ 1.75–1.62 (m, 1H), δ 2.03–1.89 (m, 1H), δ 3.47–3.35 (m, 5H), δ 3.62–3.56 (m, 1H), δ 3.68 (t, *J* = 5.5 Hz, 1H), δ 3.80–3.72 (m, 1H), δ 7.11 (d, *J* = 8.2 Hz, 2H), and δ 7.74 (d, *J* = 8.2 Hz, 2H). LRMS (ESI) m/z calcd for C_17_H_24_IN_2_O_3_ [M + H]^+^ 431.2, found 431.0.

##### 4.1.1.9 *tert*-Butyl 4-(3,4-dimethoxybenzoyl)-1,4-diazepane-1-carboxylate (3i)

Yellow oil; Yield: 54%; ^1^H NMR (DMSO-*d*_*6*_, 300 MHz): δ 1.42–1.18 1.36 (m, 9H), δ 1.62–1.47 (m, 1H), δ 1.77–1.67 (m, 1H), δ 3.38–3.33 (m, 4H), δ 3.54–3.43 (m, 4H), δ 3.72 (s, 3H), δ 3.75 (s, 3H), and δ 7.03–6.74 (m, 3H).

##### 4.1.1.10 *tert*-Butyl 4-(3,4-difluorobenzoyl)-1,4-diazepane-1-carboxylate (3j)

Yellow oil; Yield: 58%; ^1^H NMR (CDCl_3_, 300 MHz): δ 1.45–1.36 (m, 9H), δ 1.71–1.56 (m, 1H), δ 1.98–1.90 (m, 1H), δ 3.42 (t, *J* = 5.7 Hz, 3H), δ 3.49–3.44 (m, 2H), δ 3.61–3.55 (m, 1H), δ 3.67 (t, *J* = 5.0 Hz, 1H), δ 3.79–3.72 (m, 1H), and δ 7.22–7.09 (m, 3H).

#### 4.1.2 General procedure for the synthesis of Boc-protected 1,4-diazepane intermediates (3k–m)

The intermediates **3k–m** were synthesized via amide coupling of the respective carboxylic acids with 1-Boc-homopiperazine **1** in the presence of coupling agents and a base (Chan and Cox, 2007). In a 100 mL RBF, appropriate carboxylic acid derivatives (**2k–m**, 1.0 eq.) were dissolved in 10 mL of THF. To this solution, HOBt (1.0 g, 7.4 mmol, 1.5 eq.) and EDC•HCl (1.3 g, 6.8 mmol, 1.4 eq.) were added, and the mixture was stirred in an ice bath for 10 min. To this, 1-Boc-homopiperazine **1** (1.0 g, 5.0 mmol, 1.0 mL) was added, followed by TEA (0.6 g, 5.9 mmol, 0.8 mL, 1.2 eq.). The reaction mixture was stirred at r. t. overnight or refluxed at 70 °C. After completion, the solvent was removed under reduced pressure, and the residue was diluted with 10 mL of brine. The organic phase was extracted with EtOAc (3 × 10 mL). The combined organic extracts were dried over anhydrous MgSO_4_, filtered, and concentrated *in vacuo*. The crude product was purified by silica gel column chromatography using either DCM:MeOH (15:1) or EtOAc:Hexane (7:3) as the eluent, affording intermediates **3k–m** in yields ranging from 27% to 74%. ^1^H NMR data for **3k–m** are provided below:

##### 4.1.2.1 *tert*-Butyl 4-(4-hydroxy-3-methoxybenzoyl)-1,4-diazepane-1-carboxylate (3k)

Brown oil; Yield: 27%; ^1^H NMR (DMSO-*d*_*6*_, 300 MHz): δ 1.37–1.19 (m, 9H), δ 1.61–1.50 (m, 1H), δ 1.76–1.64 (m, 1H), δ 3.29–3.23 (m, 1H), δ 3.38–3.32 (m, 4H), δ 3.54–3.41 (m, 3H), δ 3.78 (s, 3H), δ 6.80–6.67 (m, 2H), δ 6.91–6.81 (m, 1H), and δ 9.33 (br s, 1H).

##### 4.1.2.2 *tert*-Butyl 4-(3,4-dihydroxybenzoyl)-1,4-diazepane-1-carboxylate (3l)

White crystalline solid; Yield: 74%; m. p. 207–209 °C. ^1^H NMR (DMSO-*d*_*6*_, 300 MHz): δ 1.40–1.19 (m, 9H), δ 1.60–1.49 (m, 1H), δ 1.77–1.61 (m, 1H), δ 3.39–3.32 (m, 4H), δ 3.63–3.40 (m, 4H), δ 6.61 (d, *J* = 7.2 Hz, 1H), δ 6.75–6.85 (m, 2H), δ 9.11 (br s, 1H), and δ 9.24 (br s, 1H).

##### 4.1.2.3 *tert*-Butyl 4-(4-benzo[*d*][1,3]dioxole-5-carbonyl)-1,4-diazepane-1-carboxylate (3m)

Yellow oil; Yield: 67%; ^1^H NMR (DMSO-*d*_*6*_, 300 MHz): δ 1.39–1.21 (m, 9H), δ 1.60–1.46 (m, 1H), δ 1.78–1.63 (m, 1H), δ 3.36–3.31 (m, 3H), δ 3.45–3.37 (m, 2H), δ 3.55–3.46 (m, 2H), δ 3.71–3.57 (m, 1H), δ 6.03 (s, 2H), and δ 6.94–6.83 (m, 3H).

#### 4.1.3 General procedure for the synthesis of (1,4-diazepan-1-yl) (phenyl)methanone derivatives (4a–m)

The Boc-protected intermediates (**3a–m**) were subjected to deprotection to afford the corresponding final amines (**4a–m**) (Takaishi et al., 2018). Deprotection was carried out by dissolving 0.1 g of the Boc-protected derivative in 7–10 mL of DCM in a 250 mL RBF and cooled in an ice bath under continuous stirring for 10 min. To this, TFA (1 mL) was added dropwise, and the reaction mixture was stirred at 0 °C for an additional 10 min before allowing the reaction to proceed at r. t. for 1–2 h. The reaction mixture was treated with 10 mL of NaHCO_3_ solution while keeping it in an ice bath. Following this, the DCM layer was extracted with brine, dried over anhydrous MgSO_4_, filtered, and concentrated *in vacuo* to obtain the crude product. Final purification was carried out using silica gel column chromatography, employing a DCM:MeOH (9:1) solvent system as the eluent. The yields of the final compounds ranged from 12% to 63%. Spectral data for all purified compounds are presented below:

##### 4.1.3.1 (1,4-Diazepan-1-yl) (phenyl)methanone (4a)

Yellow oil; Yield: 61% (25); ^1^H NMR (CDCl_3_, 300 MHz): δ 1.73 (br s, 1H), δ 2.01–1.86 (m, 1H), δ 2.87–2.78 (m, 1H), δ 3.01–2.88 (m, 2H), δ 3.13–3.03 (m, 1H), δ 3.24–3.15 (m, 1H), δ 3.48–3.37 (m, 2H), δ 3.83–3.69 (m, 2H), and δ 7.36–7.25 (m, 5H). ^13^C NMR (CDCl_3_, 75 MHz): δ 28.95, δ 31.09, δ 45.24, δ 47.34, δ 48.08, δ 48.59, δ 48.71, δ 48.78, δ 50.39, δ 52.31, δ 126.45, δ 126.61, δ 128.45, δ 129.33, δ 136.81, and δ 171.78. HRMS (ESI) m/z calcd for C_12_H_17_N_2_O [M + H]^+^ 205.1341, found 205.1337. Purity: > 95% (LCMS).

##### 4.1.3.2 (1,4-Diazepan-1-yl) (p-tolyl)methanone (4b)

Brown oil; Yield: 57%; ^1^H NMR (CDCl_3_, 300 MHz): δ 1.67 (br s, 1H), δ 1.93–1.84 (m, 1H), δ 2.18–2.12 (m, 1H), δ 2.33–2.24 (s, 3H), δ 2.84–2.76 (m, 1H), δ 2.96–2.85 (m, 2H), δ 3.07–2.97 (m, 1H), δ 3.48–3.35 (m, 2H), δ 3.79–3.66 (m, 2H), δ 7.16 (d, *J* = 7.7 Hz, 2H), and δ 7.26 (d, *J* = 7.7 Hz, 2H). ^13^C NMR (CDCl_3_, 75 MHz): δ 21.37, δ 28.46, δ 30.12, δ 45.22, δ 46.64, δ 47.51, δ 48.39, δ 48.69, δ 50.04, δ 51.71, δ 126.63, δ 129.03, δ 133.57, δ 139.44, and δ 172.00. HRMS (ESI) m/z calcd for C_13_H_19_N_2_O [M + H]^+^ 219.1497, found 219.1496. Purity: > 95% (LCMS).

##### 4.1.3.3 (1,4-Diazepan-1-yl) (4-methoxyphenyl)methanone (4c)

Brown oil; Yield: 25%; ^1^H NMR (CDCl_3_, 300 MHz): δ 1.69 (br s, 1H), δ 1.92–1.82 (m, 1H), δ 2.11–2.06 (m, 1H), δ 2.94–2.78 (m, 3H), δ 3.07–2.95 (m, 1H), δ 3.52–3.40 (m, 2H), δ 3.76–3.65 (m, 2H), δ 3.79 (s, 3H), δ 6.88 (d, *J* = 8.7 Hz, 2H), and δ 7.34 (d, *J* = 8.7 Hz, 2H). ^13^C NMR (DMSO-*d*_*6*_, 75 MHz): δ 29.48, δ 31.74, δ 44.97, δ 47.53, δ 48.23, δ 48.72, δ 48.94, δ 49.28, δ 50.39, δ 52.86, δ 55.62, δ 113.96, δ 128.96, δ 129.79, δ 160.14, and δ 170.65. HRMS (ESI) m/z calcd for C_13_H_19_N_2_O_2_ [M + H]^+^ 235.1447, found 235.1438. Purity: > 95% (LCMS).

##### 4.1.3.4 (1,4-Diazepan-1-yl) (4-nitrophenyl)methanone (4d)

Brown powder; Yield: 61%; m. p. 113 °C–115 °C. ^1^H NMR (CDCl_3_, 300 MHz): δ 1.71–1.63 (m, 1H), δ 1.95–1.85 (m, 2H), δ 2.95–2.82 (m, 3H), δ 3.07 (t, *J* = 5.3, 1H), δ 3.41–3.33 (m, 2H), δ 3.83–3.73 (m, 2H), δ 7.56 (d, *J* = 8.5 Hz, 2H), and δ 8.27 (dd, *J* = 8.5, 3.0 Hz, 2H). ^13^C NMR (DMSO-*d*_*6*_, 75 MHz): δ 29.21, δ 31.20, δ 44.91, δ 46.87, δ 47.27, δ 47.75, δ 48.51, δ 48.68, δ 49.80, δ 52.25, δ 124.15, δ 128.36, δ 143.99, δ 147.93, and δ 168.90. HRMS (ESI) m/z calcd for C_12_H_16_N_3_O_3_ [M + H]^+^ 250.1192, found 250.1190. Purity: > 95% (LCMS).

##### 4.1.3.5 (1,4-Diazepan-1-yl) (4-fluorophenyl)methanone (4e)

White powder; Yield: 38%; m. p. 55 °C–57 °C. ^1^H NMR (CDCl_3_, 300 MHz): δ 1.68 (br s, 1H), δ 1.95–1.85 (m, 1H), δ 2.17–2.12 (m, 1H), δ 2.85–2.78 (m, 1H), δ 2.94–2.86 (m, 2H), δ 3.08–2.99 (m, 1H), δ 3.47–3.36 (m, 2H), δ 3.78–3.68 (m, 2H), δ 7.07 (t, *J* = 8.5 Hz, 2H), and δ 7.39 (dd, *J* = 8.4, 5.5 Hz, 2H). ^13^C NMR (DMSO-*d*_*6*_, 75 MHz): δ 29.41, δ 31.59, δ 44.96, δ 47.48, δ 48.12, δ 48.67, δ 48.88, δ 49.19, δ 50.17, δ 52.70, δ 115.55 (d, ^2^J_CF_ = 21.4 Hz), δ 129.18 (dd, ^3^J_CF_ = 8.3 Hz, ^2^J_CF_ = 22.5 Hz), δ 134.14, δ 161.01, δ 164.26, and δ 169.86. HRMS (ESI) m/z calcd for C_12_H_16_FN_2_O [M + H]^+^ 223.1247, found 223.1245. Purity: > 95% (LCMS).

##### 4.1.3.6 (4-Chlorophenyl) (1,4-diazepan-1-yl)methanone (4f)

Brown solid; Yield: 62%; m. p. 99 °C–101 °C. ^1^H NMR (CDCl_3_, 300 MHz): δ 1.68 (br s, 1H), δ 1.98–1.82 (m, 1H), δ 2.26–2.08 (m, 1H), δ 2.85–2.79 (m, 1H), δ 2.94–2.86 (m, 2H), δ 3.07–3.02 (m, 1H), δ 3.52–3.33 (m, 2H), δ 3.85–3.65 (m, 2H), and δ 7.38–7.28 (m, 4H). ^13^C NMR (CDCl_3_, 75 MHz): δ 28.93, δ 31.12, δ 45.26, δ 47.46, δ 48.10, δ 48.70, δ 48.78, δ 49.00, δ 50.40, δ 52.40, δ 128.03, δ 128.22, δ 128.70, δ 135.23, and δ 170.65. HRMS (ESI) m/z calcd for C_12_H_16_ClN_2_O [M + H]^+^ 239.0951, found 239.0943. Purity: > 95% (LCMS).

##### 4.1.3.7 (4-Bromophenyl) (1,4-diazepan-1-yl)methanone (4g)

Brown solid; Yield: 63%; m. p. 89 °C–91 °C. ^1^H NMR (CDCl_3_, 300 MHz): δ 1.64 (br s, 1H), δ 1.89–1.83 (m, 1H), δ 1.95–1.90 (m, 1H), δ 2.81–2.75 (m, 1H), δ 2.90–2.83 (m, 2H), δ 3.03–2.97 (m, 1H), δ 3.40–3.33 (m, 2H), δ 3.81–3.62 (m, 2H), δ 7.24 (d, *J* = 8.4 Hz, 2H), and δ 7.49 (d, *J* = 7.9 Hz, 2H). ^13^C NMR (CDCl_3_, 75 MHz): δ 29.10, δ 31.55, δ 45.29, δ 47.75, δ 48.39, δ 48.71, δ 49.13, δ 49.28, δ 50.57, δ 52.62, δ 123.49, δ 128.22, δ 128.44, δ 131.65, δ 135.78, and δ 170.64. HRMS (ESI) m/z calcd for C_12_H_16_BrN_2_O [M + H]^+^ 283.0446, found 283.0439. Purity: > 95% (LCMS).

##### 4.1.3.8 (1,4-Diazepan-1-yl) (4-iodophenyl)methanone (4h)

White solid; Yield: 53%; m. p. 121 °C–123 °C. ^1^H NMR (CDCl_3_, 300 MHz): δ 1.72 (br s, 1H), δ 1.96–1.84 (m, 1H), δ 2.68–2.58 (m, 1H), δ 2.87–2.79 (m, 1H), δ 2.97–2.88 (m, 2H), δ 3.12–3.03 (m, 1H), δ 3.45–3.34 (m, 2H), δ 3.80–3.69 (m, 2H), δ 7.13 (d, *J* = 8.2 Hz, 2H), and δ 7.73 (d, *J* = 8.0 Hz, 2H). ^13^C NMR (CDCl_3_, 75 MHz): δ 29.00, δ 31.21, δ 45.26, δ 47.56, δ 48.22, δ 48.68, δ 48.87, δ 49.08, δ 50.56, δ 52.50, δ 95.44, δ 128.29, δ 128.48, δ 136.24, δ 137.59, and δ 170.75. HRMS (ESI) m/z calcd for C_12_H_16_IN_2_O [M + H]^+^ 331.0307, found 331.0298. Purity: > 95% (LCMS).

##### 4.1.3.9 (1,4-Diazepan-1-yl) (3,4-dimethoxyphenyl)methanone (4i)

Yellow oil; Yield: 57%; ^1^H NMR (DMSO-*d*_*6*_, 300 MHz): δ 1.76–1.66 (m, 2H), δ 2.51–2.43 (m, 1H), δ 2.90–2.79 (m, 1H), δ 3.44–3.30 (m, 5H), δ 3.61–3.45 (m, 2H), δ 3.74 (d, J = 4.0 Hz, 6H), and δ 7.02–6.82 (m, 3H). ^13^C NMR (CDCl_3_, 75 MHz): δ 28.98, δ 29.68, δ 31.08, δ 45.49, δ 47.26, δ 47.96, δ 48.44, δ 48.91, δ 50.44, δ 52.42, δ 55.95, δ 55.98, δ 110.40, δ 110.54, δ 119.56, δ 129.04, δ 148.86, δ 149.97, and δ 171.64. HRMS (ESI) m/z calcd for C_14_H_21_N_2_O_3_ [M + H]^+^ 265.1552, found 265.1545. Purity: > 95% (UPLC).

##### 4.1.3.10 (1,4-Diazepan-1-yl) (3,4-difluorophenyl)methanone (4j)

Yellow oil; Yield: 12%; ^1^H NMR (DMSO-*d*_*6*_, 300 MHz): δ 1.53 (br, s, 1H), δ 1.78–1.63 (m, 1H), δ 2.77–2.62 (m, 3H), δ 2.86–2.78 (m, 1H), δ 3.34–3.28 (m, 3H), δ 3.59–3.52 (m, 2H), δ 7.31–7.13 (m, 1H), and δ 7.58–7.38 (m, 2H). ^13^C NMR (DMSO-*d*_*6*_, 75 MHz): δ 28.79, δ 30.30 δ 44.87, δ 46.51 δ 47.08, δ 47.58, δ 48.39, δ 48.69, δ 49.31, δ 51.70, δ 117.17–115.95 (m), δ 118.69–117.53 (m), δ 134.83 δ 148.96–147.41 (m) 152.38–150.72 (m), and δ 168.62. HRMS (ESI) m/z calcd for C_12_H_15_F_2_N_2_O [M + H]^+^ 241.1152, found 241.1150. Purity: > 95% (UPLC).

##### 4.1.3.11 (1,4-Diazepan-1-yl) (4-hydroxy-3-methoxyphenyl)methanone (4k)

Purple oil; Yield: 40%; ^1^H NMR (DMSO-*d*_*6*_, 300 MHz): δ 2.02–1.91 (m, 2H), δ 3.54–3.08 (m, 8H), δ 3.84–3.69 (m, 4H), δ 6.87–6.76 (m, 2H), δ 7.04–6.96 (m, 1H), and δ 9.40 (br, s, 1H). ^13^C NMR (DMSO-*d*_*6*_, 75 MHz): δ 26.03, δ 42.13, δ 44.19, δ 45.07, δ 48.72, δ 56.19, δ 111.99, δ 115.38, δ 120.49, δ 127.33, δ 147.62, δ 148.35, and δ 171.06. HRMS (ESI) m/z calcd for C_13_H_19_N_2_O_3_ [M + H]^+^ 251.1396, found 251.1390. Purity: > 95% (LCMS).

##### 4.1.3.12 (1,4-Diazepan-1-yl) (3,4-dihydroxyphenyl)methanone (4l)

Greenish oil; Yield: 47%; ^1^H NMR (DMSO-*d*_*6*_, 300 MHz): δ 1.83–1.66 (m, 2H), δ 2.93–2.78 (m, 3H), δ 3.06–2.94 (m, 1H), δ 3.50–3.31 (m, 3H), δ 3.66–3.51 (m, 2H), δ 6.66 (dd, *J* = 9.5, 1.8 Hz, 1H), δ 6.72 (d, *J* = 8.0 Hz, 1H), and δ 6.77–6.74 (m, 1H). ^13^C NMR (DMSO-*d*_*6*_, 75 MHz): δ 23.06, δ 25.73, δ 29.43, δ 45.58, δ 48.51, δ 115.36, δ 115.85, δ 119.15, δ 127.24, δ 145.29, δ 147.26, and δ 171.41. HRMS (ESI) m/z calcd for C_12_H_16_N_2_O_3_ [M + H]^+^ 237.1239, found 237.1231. Purity: > 95% (LCMS).

##### 4.1.3.13 Benzo[*d*][1,3]dioxole-5-yl-(1,4-Diazepan-1-yl)methanone (4m)

Yellow oil; Yield: 27%; ^1^H NMR (DMSO-*d*_*6*_, 300 MHz): δ 1.72–1.52 (m, 2H), δ 2.78–2.65 (m, 3H), δ 2.87–2.81 (m, 1H), δ 3.33–3.20 (m, 3H), δ 3.63–3.43 (m, 2H), δ 6.03 (s, 2H), δ 6.85 (dd, *J* = 8.0, 1.5 Hz, 1H), and δ 6.94–6.88 (m, 2H). ^13^C NMR (CDCl_3_, 75 MHz): δ 28.72, δ 29.68, δ 30.38, δ 45.35, δ 46.97, δ 47.88, δ 48.80, δ 50.32, δ 52.16, δ 101.38, δ 107.60, δ 108.24, δ 120.94, δ 130.20, δ 147.56, δ 148.54, and δ 171.25. HRMS (ESI) m/z calcd for C_13_H_17_N_2_O_3_ [M + H]^+^ 249.1239, found 249.1237. Purity: > 95% (LCMS).

#### 4.1.4 General procedure for the synthesis of (4-aminophenyl) (1,4-diazepan-1-yl)methanone (4n)

Compound **4n** was prepared via catalytic reduction of the corresponding nitro compound **4d** (Qiu et al., 2019). In a 100 mL RBF, **4d** (0.5 g, 2.01 mmol) and 10% Pd/C (0.15 g, 1.41 mmol) were dissolved in 15 mL of ethanol. Hydrazine hydrate (0.7 mL, 21.84 mmol) was added dropwise to the reaction mixture under continuous stirring. The resulting mixture was refluxed at 80 °C for 2 h, cooled to r. t., and the Pd/C was removed by filtration through a cotton plug by washing with ethanol (3 × 25 mL). The combined ethanol fractions were concentrated under reduced pressure to afford the crude residue. Purification by silica gel column chromatography using DCM:MeOH (9:1) as the eluent afforded compound **4n** in 89% yield. Analytical data for compound **4n** is provided below:

##### 4.1.4.1 (4-Aminophenyl) (1,4-diazepan-1-yl)methanone (4n)

White powder; Yield: 89%; m. p. 187 °C–189 °C. ^1^H NMR (DMSO-*d*_*6*_, 300 MHz): δ 1.68–1.57 (m, 2H), δ 2.52–2.44 (m, 1H), δ 3.36–3.31 (m, 3H), δ 3.43–3.37 (m, 3H), δ 3.51–3.44 (m, 2H), δ 5.35 (s, 2H), δ 6.51 (d, *J* = 8.3 Hz, 2H), and δ 7.06 (d, *J* = 8.3 Hz, 2H). ^13^C NMR (DMSO-*d*_*6*_, 75 MHz): δ 29.79, δ 31.78, δ 45.08, δ 48.79, δ 53.02, δ 113.14, δ 124.13, δ 128.91, δ 150.31, and δ 171.48. HRMS (ESI) m/z calcd for C_12_H_18_N_3_O [M + H]^+^ 220.1450, found 220.1447. Purity: > 95% (UPLC).

### 4.2 Biology

#### 4.2.1 Methodology for the ThT-based Aβ aggregation kinetic study

The anti-aggregation activity of compounds **4a–n** toward Aβ42 and Aβ40 was evaluated using the thioflavin T (ThT)-based fluorescence assay (Karuturi et al., 2026; Mohamed et al., 2016). The assay was conducted in black, clear-bottom 384-well plates (Nunc®), with fluorescence intensity (RFU) recorded at 440 nm excitation and 490 nm emission using a BioTek Synergy H1 plate reader. The plate was incubated at 37 °C with continuous shaking (730 cpm, 30 s) and fluorescence readings were collected at 10-min intervals over 24 h. Compounds, including the reference agents, RVT and MB, were first dissolved in DMSO to obtain 10 mM stock solutions, ensuring a final DMSO concentration of ≤ 2% (v/v) in the assay. These were diluted in 215 mM sodium phosphate buffer (Na_2_HPO_4_.7H_2_O, pH 7.4) to achieve final concentrations of 1, 5, 10, and 25 µM. The Aβ.HFIP samples (Aβ42/Aβ40, ≥ 95% pure, rPeptide, GA, United States) were solubilized in 10% NH_4_OH (Aβ42) or 1% NH_4_OH (Aβ40), followed by dilution in phosphate buffer to 50 µM and sonication on ice for 5 min. A freshly prepared 15 µM ThT solution was made in 50 mM glycine-NaOH buffer (pH 7.4) and protected from light throughout the experiment. Each well contained 40 µL of a sample solution containing ThT (11 µl), phosphate buffer, DMSO, the test compound (4 µl), and either Aβ42 or Aβ40 (8 µl each). Control wells included the ThT background, Aβ control, compound control, and phosphate buffer. Fluorescence data at 24 h were used to calculate the percentage inhibition of Aβ42/Aβ40 aggregation. Results represent the mean ± SD of triplicate measurements from three independent experiments.

#### 4.2.2 Transmission electron microscopy (TEM)

The morphology of Aβ42/40 peptide aggregates in the presence of compounds **4k**, **4l**, and **4m** was examined using transmission electron microscopy (TEM) (Karuturi et al., 2026). Imaging was conducted on a Philips CM 10 TEM instrument operating at 60 k, Department of Biology, University of Waterloo. Micrographs were captured with a 14-megapixel AMT camera. After completing the 24 h ThT fluorescence aggregation assay in the presence of Aβ42 or Aβ40 and test compounds **4k**, **4l**, or **4m** (25 µM each), aliquots were taken from the 384-well plates. The TEM grids were prepared by applying 20 µL of the sample to formvar-coated 400-mesh copper grids using an Eppendorf pipette, then allowed to air-dry overnight. To remove excess buffer salts, the grids were carefully rinsed with 2–3 drops (~ 40 µL) of ultrapure water (UPW) and dried carefully using filter paper. Grids were then stained with 20 µL of 2% phosphotungstic acid (PTA), immediately blotted dry using filter paper, rinsed three times with 40 µL UPW, and finally air-dried overnight prior to image acquisition.

#### 4.2.3 Cell viability assay

The neuroprotective effects of compounds **4g**, **4k**, **4l**, and **4m** were evaluated in mouse hippocampal HT22 cells exposed to Aβ42 using a cell viability assay (Karuturi et al., 2026) based on the Cell Counting Kit-8 (CCK-8, TargetMol, MA, United States). This colorimetric assay is driven by the enzymatic conversion of the water-soluble tetrazolium salt WST-8 [2-(2-methoxy-4-nitrophenyl)-3-(4-nitrophenyl)-5-(2,4-disulfophenyl)-2*H*-tetrazolium, monosodium salt] into an orange formazan dye by intracellular dehydrogenases. The extent of formazan formation, measured by absorbance at 450 nm, directly reflects the viable cells. HT22 cells were cultured in 96-well plates at a density of 5 × 10^4^ cells/mL using a 50:50 mixture of Dulbecco’s Modified Eagle Medium (DMEM) and Ham’s F12, supplemented with 10% fetal bovine serum (FBS) and 1% penicillin-streptomycin (10,000 U/mL), and incubated at 37 °C in 5% CO_2_. After 24 h of incubation, the medium was replaced with DMEM/F12 without FBS, prior to treatment. The test compounds and the reference control RVT were added at a final concentration of 25 μM, prepared in PBS and < 2% DMSO. Oligomeric Aβ42 was made by dissolving Aβ42. HFIP (purity > 95%, Bachem, Switzerland) in DMSO and subsequently diluted in DMEM to achieve a 200 µM stock solution, which was stored at 4 °C for 24 h. Following this, the cells were co-incubated with 10 μM Aβ42 and either compounds (**4g**, **4k**, **4l**, and **4m**) or RVT for 48 h at 37 °C. Post-treatment, CCK-8 reagent was added to each well, followed by a 2 h incubation. Absorbance was recorded at 450 nm using a microplate reader. Cell viability was calculated as the mean of quadruplicates (n = 4) across two or three independent experiments, and statistical analysis was performed using one-way ANOVA followed by Bonferroni *post hoc* tests.

#### 4.2.4 DPPH scavenging assay

The antioxidant potential of selected 1,4-diazepane derivatives (**4a**, **4k**, and **4l**) was evaluated to determine their ability to neutralize the 2,2-diphenyl-1-picrylhydrazyl radical (DPPH•). This assay relies on the reduction of the purple-coloured DPPH• radical to a yellow-coloured form (DPPH) (Kleinrichert and Alappat, 2019). The change in colour intensity was quantified spectrophotometrically to determine the radical scavenging activity of the compounds. Test samples (**4a**, **4k**, and **4l**), alongside reference agents RVT and trolox, were prepared in anhydrous methanol at a concentration of 25 µM. A 0.09 mM DPPH stock solution was also prepared in anhydrous methanol. The assay was performed in a clear 96-well microplate, with 25 µL of each test compound combined with 100 µL of DPPH solution for compound screening. Several controls were included: 25 µL of test compound with 100 µL methanol, 25 µL methanol with 100 µL DPPH solution, and 125 µL methanol alone. After that, the plate was incubated for 1 h at room temperature with continuous shaking, protected from light. Absorbance was recorded at 517 nm using a Varioskan LUX multimode microplate reader. Data are presented as the mean percentage of DPPH radical scavenging activity, calculated from triplicate measurements (n = 3) from two independent experiments.

#### 4.2.5 H_2_O_2_-induced cytotoxicity assay

The cytoprotective effects of compounds **4a**, **4k**, and **4l** toward H_2_O_2_-induced cytotoxicity were assessed in HT22 mouse hippocampal neuronal cells (Hefny et al., 2025). Cells were seeded at 5 × 10^4^ cells/mL in 96-well plates and cultured in complete growth medium (DMEM/Ham’s F-12, 50:50), supplemented with 10% fetal bovine serum and 1% penicillin-streptomycin (10,000 U/mL), at 37 °C in 5% CO2. Cells were allowed to reach ∼80% confluency over 48 h. Following 24 h incubation at 37 °C, cells were treated with either **4a**, **4k** or **4l** (25 µM) for 2 h. This was followed by exposure to H_2_O_2_ (200 µM) for an additional 24 h. Cell viability was subsequently assessed using the CCK-8 assay. After 2 h, absorbance was recorded at 450 nm using a microplate reader. Data represent the mean of quadruplicates (n = 4) from two independent experiments, and statistical analysis was performed using one-way ANOVA followed by Bonferroni *post hoc* tests.

#### 4.2.6 BBB permeability assay

The blood-brain barrier (BBB) permeability of compounds **4a**, **4g**, **4k**, **4l**, and **4m** was evaluated using the parallel artificial membrane permeability assay (PAMPA) in a 96-well sandwich plate format (BioAssay Systems, CA, United States) (Gujral et al., 2022). This system consists of a donor plate and an acceptor plate separated by a lipid-infused artificial membrane. Stock solutions of compounds and permeability controls (high = promazine HCl, medium = clonidine, and low = diclofenac) were initially prepared at 10 mM in DMSO and subsequently diluted in phosphate-buffered saline (PBS) to a final concentration of 500 µM. Equilibrium standards at 200 µM were prepared by mixing 80 µL of the 500 µM solution with 120 µL of PBS, alongside a DMSO blank control (5 µL DMSO in 245 µL PBS). The acceptor plate wells were filled with 300 µL PBS prior to adding 200 µL of each test compound or control to duplicate wells in the donor plate, which contained 5 µL of dodecane. The donor plate was then placed over the acceptor plate, and the assembly was incubated at 37 °C for 18 h. Post-incubation, 100 µL of the acceptor solution was transferred to a fresh 96-well plate containing 100 µL of the corresponding equilibrium standard for UV absorbance measurement. Absorbance was recorded between 200 and 500 nm at 10 nm intervals using a microplate reader. Peak absorbance wavelengths for the high, medium, and low-permeability controls were identified at 250 nm, 250 nm, and 270 nm, respectively, in accordance with the manufacturer’s protocol. Permeability coefficient (P_e_) was calculated using mean absorbance values from duplicate wells across two independent experiments (n = 2).

#### 4.2.7 Computational modelling studies

The binding interaction between compound **4k** and Aβ42/40 aggregates was evaluated via molecular docking using BIOVIA Discovery Studio (DS) Structure-Based Design (SBD) software (San Diego, United States) (Karuturi et al., 2026; Mohamed et al., 2016). The 3D structures for the Aβ42 (PDB ID: 5KK3) and Aβ40 (PDB ID: 2LMN) were retrieved from the Protein Data Bank and were used to prepare Aβ42 pentamer and Aβ40 dimer models. The molecular structure of **4k** was constructed and energy-minimized in DS employing the small molecule module. Energy minimization of **4k** utilized the CHARMm force field, following a two-step process consisting of 1,000 steps of steepest descent, followed by 2000 steps of conjugate gradient to obtain the starting structure for docking studies. A 20 Å radius sphere covering both the N- and C-terminal regions in the Aβ42 pentamer and Aβ40 dimer was prepared as a region for ligand binding, and molecular docking was carried out using the receptor-ligand interaction protocol, applying the LibDock algorithm in DS to predict optimal binding orientations. Subsequent analyses included a detailed characterization of polar and hydrophobic contacts, as well as distance parameters between **4k** and amino acid residues of Aβ assemblies. To further quantify binding affinity, the ligand binding energies were calculated using the Generalized Born with a simple SWitching (GBSW) implicit solvent function implemented within the SBD suite (receptor-ligand interaction module), by using the following equation:

$$E_{\text{binding}}=\text{Energy of complex }\left(E_{\text{ligand}-\text{receptor}}\right)-\text{Energy of ligand }\left(E_{\text{ligand}}\right)-\text{Energy of receptor }\left(E_{\text{receptor}}\right)$$

## Data Availability

The original contributions presented in the study are included in the article/Supplementary Material, further inquiries can be directed to the corresponding author.
