# Supplementary material for: Novel 1,4-Diazepane derivatives as amyloid-beta (Aβ) aggregation inhibitors
Source: Front Chem. 2026 Sep 1;14:1919936. doi: 10.3389/fchem.2026.1919936 (PMC13574327; doi:10.3389/fchem.2026.1919936)
Supplement: Supplementary file 1 [file DataSheet1.pdf]

Supplementary Material

Novel 1,4-Diazepane Derivatives as Amyloid-Beta (Aβ) Aggregation Inhibitors

Rahul C. Karuturi[1], Ahmed A. Hefny[1], Arash Shakeri[1], and Praveen P.N. Rao[1*]

[1]School of Pharmacy, Health Sciences Campus, University of Waterloo, 200 University Avenue West, Waterloo, Ontario, N2L 3G1, Canada

[*] Corresponding author contact information: praveen.nekkar@uwaterloo.ca

Table of Contents

1. Analytical data (^{1}H NMR and LCMS) for intermediates **3a**–**m**……………………………………………………………………………….. …..S2
2. Analytical data (^{1}H NMR, ^{13}C NMR, LCMS, UPLC and HRMS) for compounds **4a**–**n**……………………………………………………………………………......…S21
3. Evaluation of compounds **4a–n** as inhibitors of Aβ40 aggregation at 25 µM…………S63
4. Evaluation of compound **4k** as inhibitor of Aβ40 aggregation…………………………S63
5. DPPH Scavenging by compounds **4a, 4k,** and **4l**………………………………………S64
6. BBB Permeability assay data for compounds **4a, 4g, 4k, 4l, 4m,** and RVT…………..S64
7. Table S1. Permeability coefficient (P_e) of **4a, 4g, 4k, 4l,** and **4m** in the PAMPA-BBB assay………………………………………………………………………………S65
8. Table S2. Summary of physicochemical and drug-likeness properties of **4k** and **4m** calculated from Web Tool SwissADME……………………………………………S66

1 ^{1}H NMR data for intermediates 3a–m

***tert*-Butyl 4-benzoyl-1,4-diazepane-1-carboxylate (3a)**

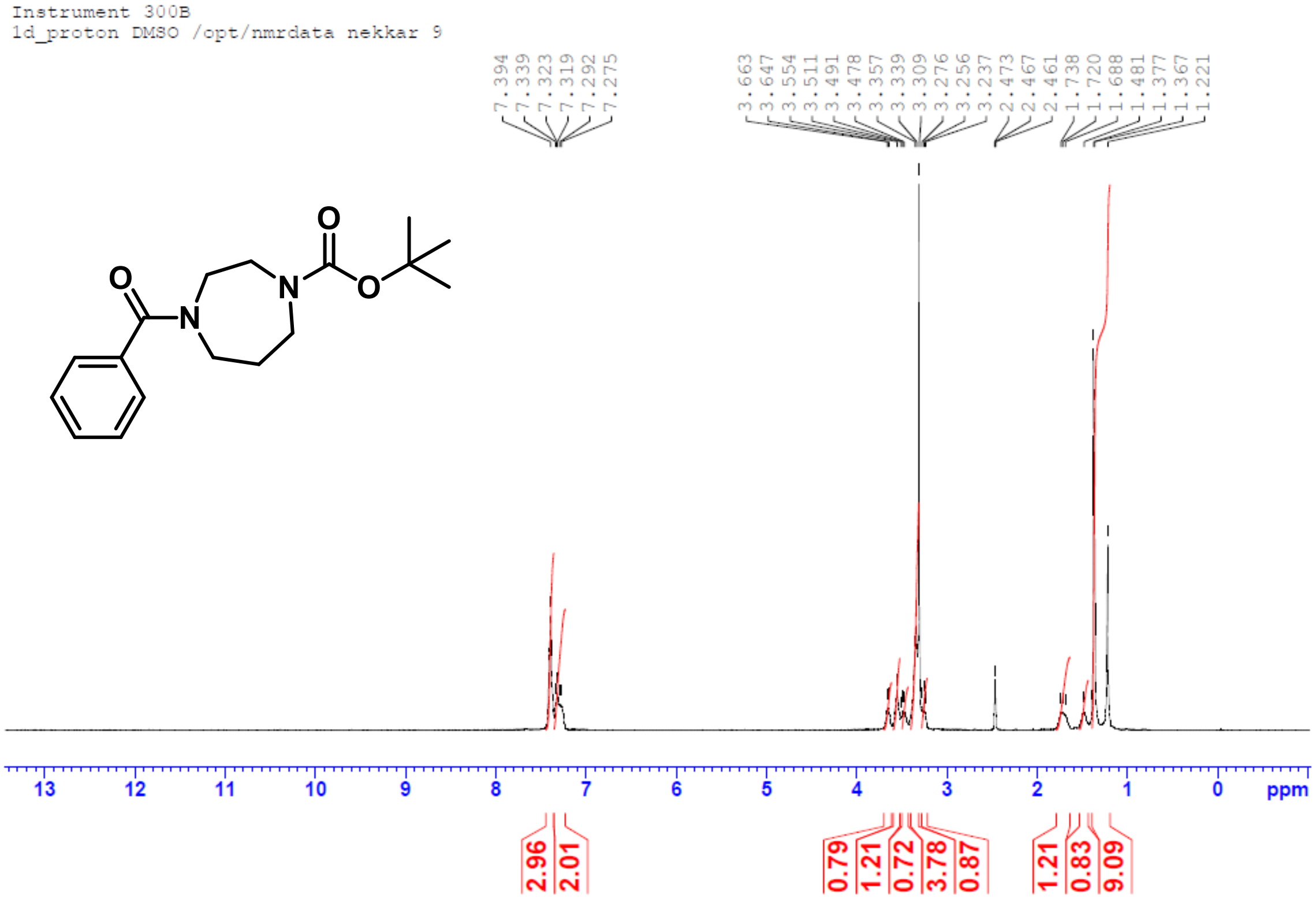

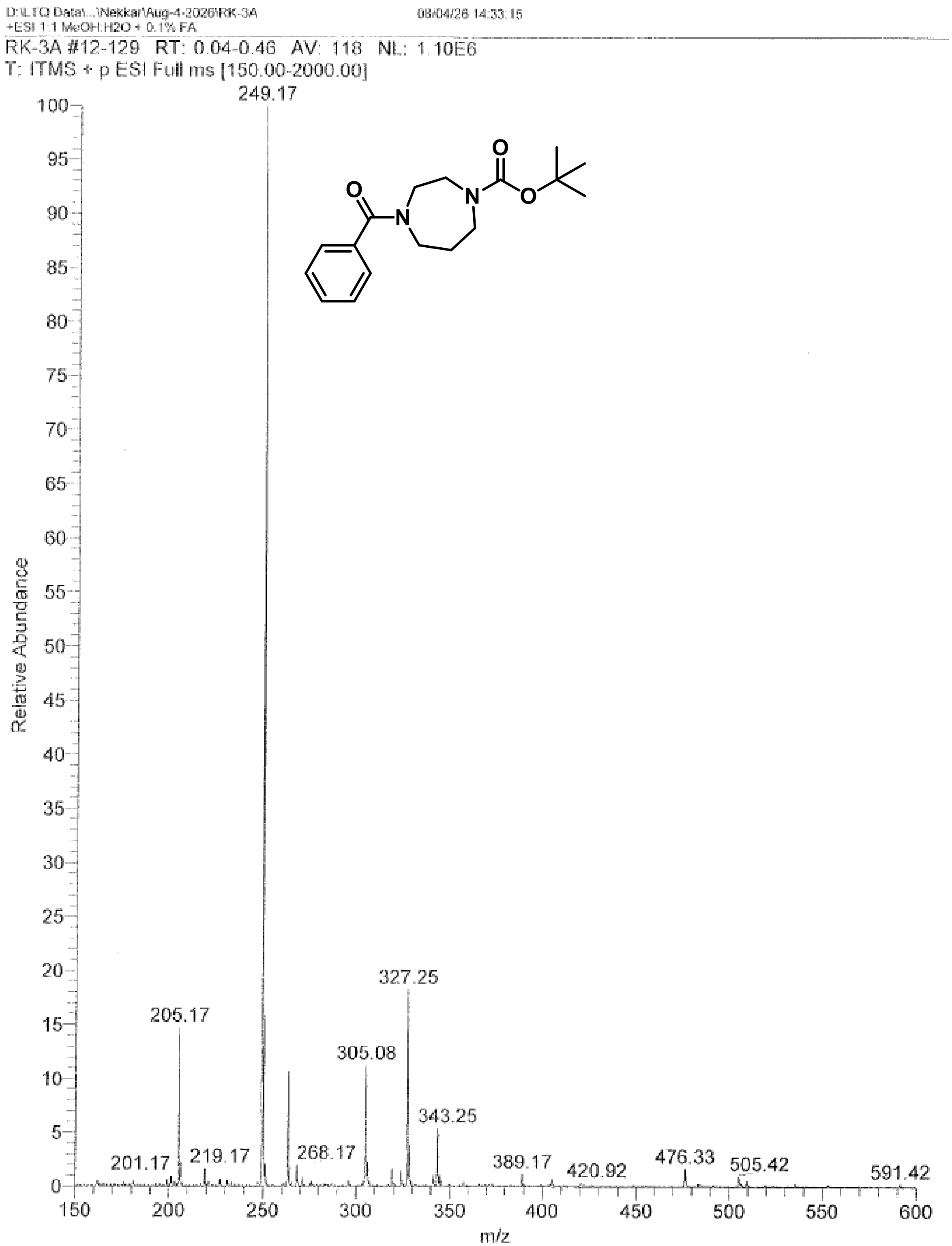

D:\LTQ Data\...\Nekkar\Aug-4-2026\RK-3A
+ESI 1:1 MeOH:H2O + 0.1% FA
08/04/26 14:33:15
RK-3A #12-129 RT: 0.04-0.46 AV: 118 NL: 1.10E6
T: ITMS + p ESI Full ms [150.00-2000.00]
249.17
205.17
327.25
305.08
343.25
201.17
219.17
268.17
389.17
420.92
476.33
505.42
591.42
Relative Abundance
m/z

***tert*-Butyl 4-(4-methylbenzoyl)-1,4-diazepane-1-carboxylate (3b)**

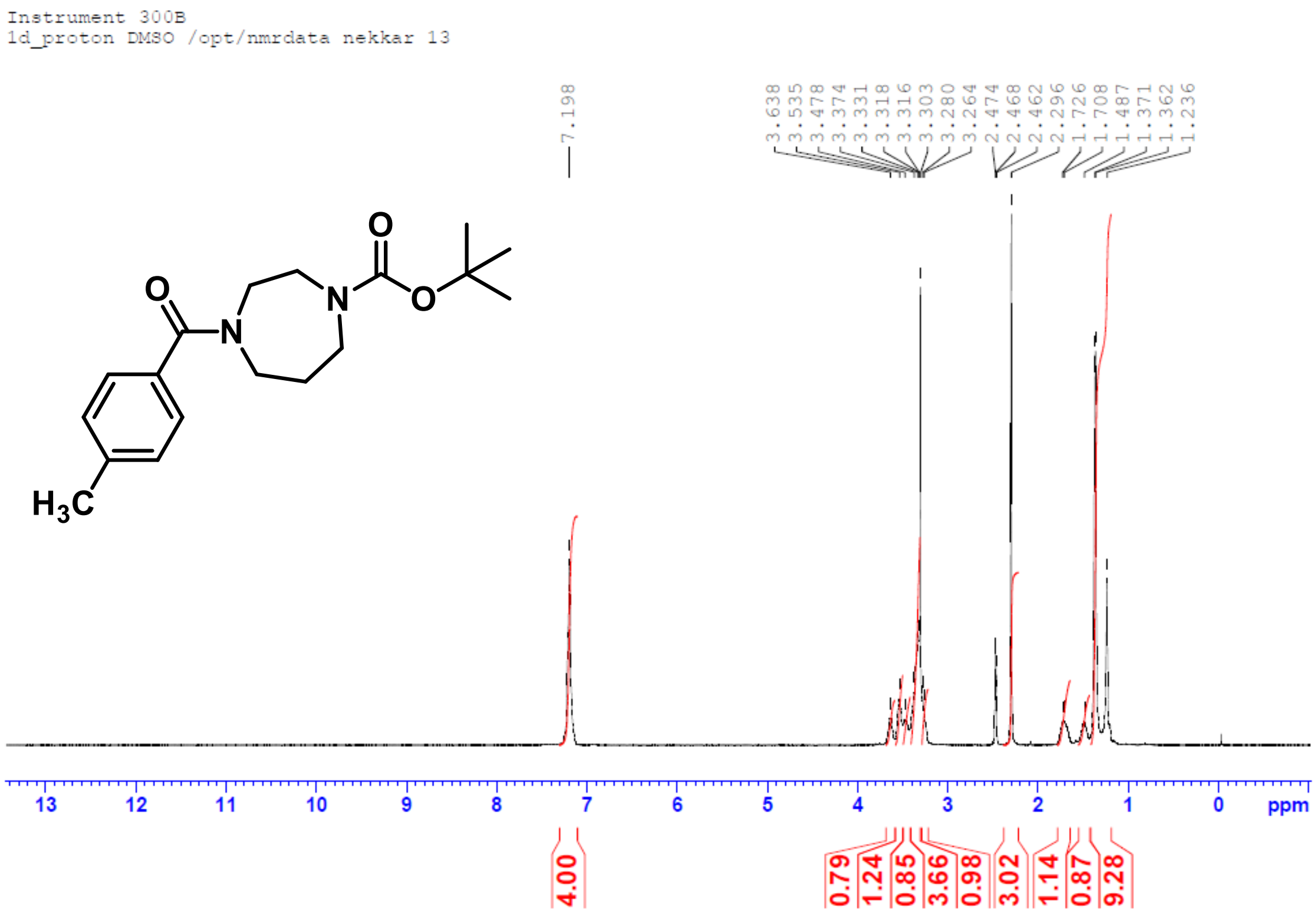

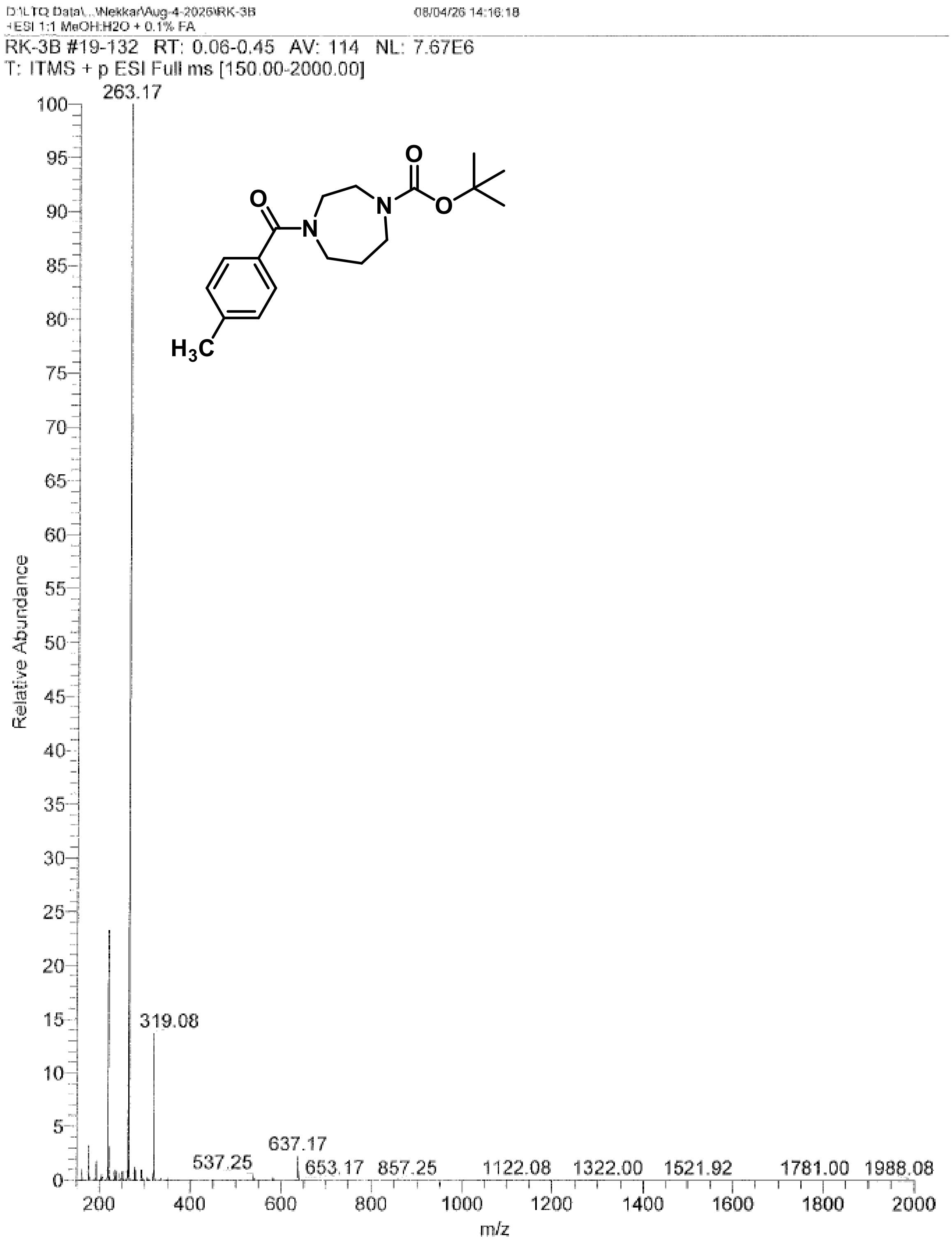
D:\LTQ Data\...\Wekkar\Aug-4-2026\RK-3B
08/04/26 14:16:18
+ESI 1:1 MeOH:H2O + 0.1% FA
RK-3B #19-132 RT: 0.06-0.45 AV: 114 NL: 7.67E6
T: ITMS + p ESI Full ms [150.00-2000.00]
263.17
319.08
537.25
637.17
653.17
857.25
1122.08
1322.00
1521.92
1781.00
1988.08
Relative Abundance
m/z
O
N
H3C

***tert*-Butyl 4-(4-methoxybenzoyl)-1,4-diazepane-1-carboxylate (3c)**

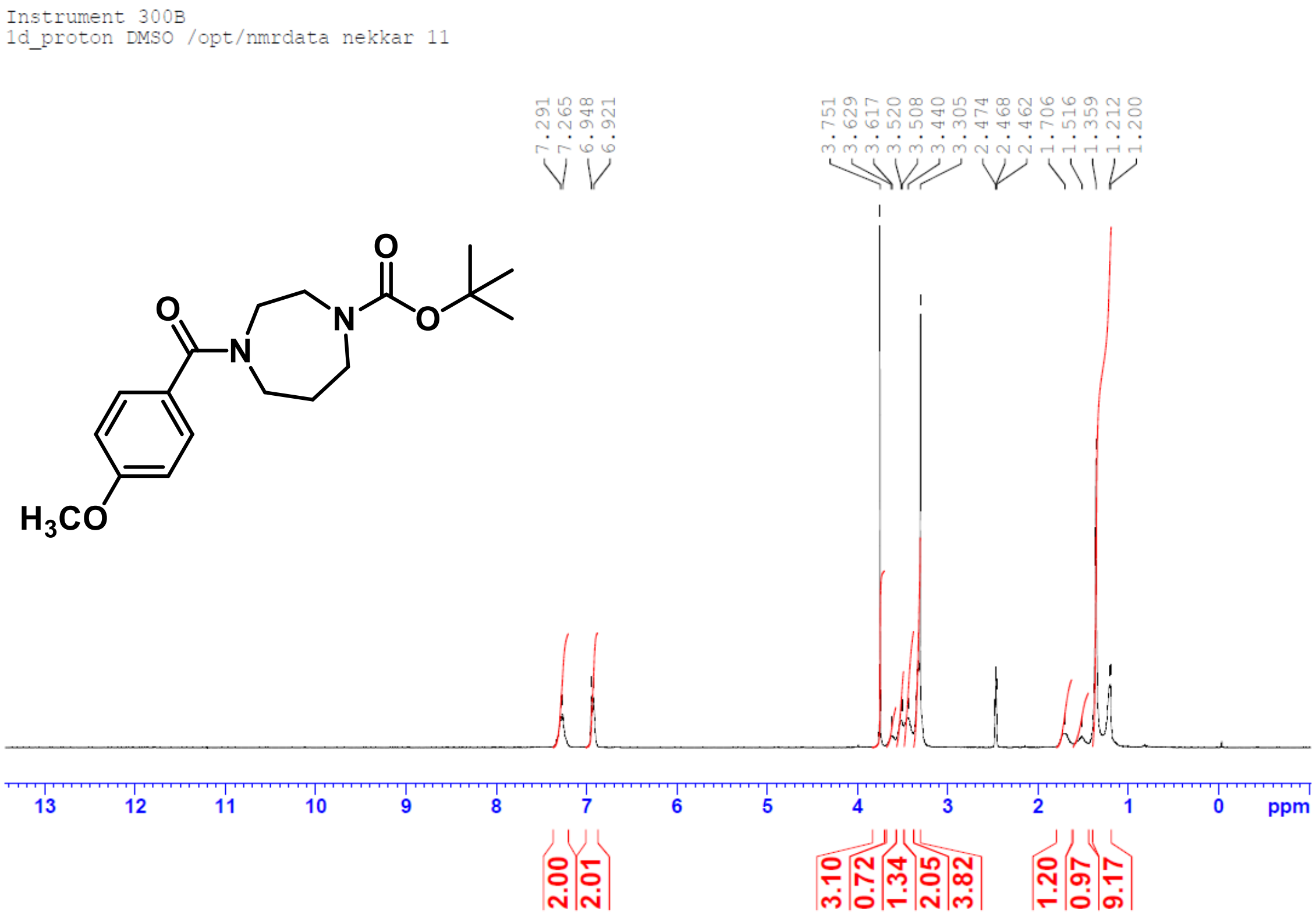

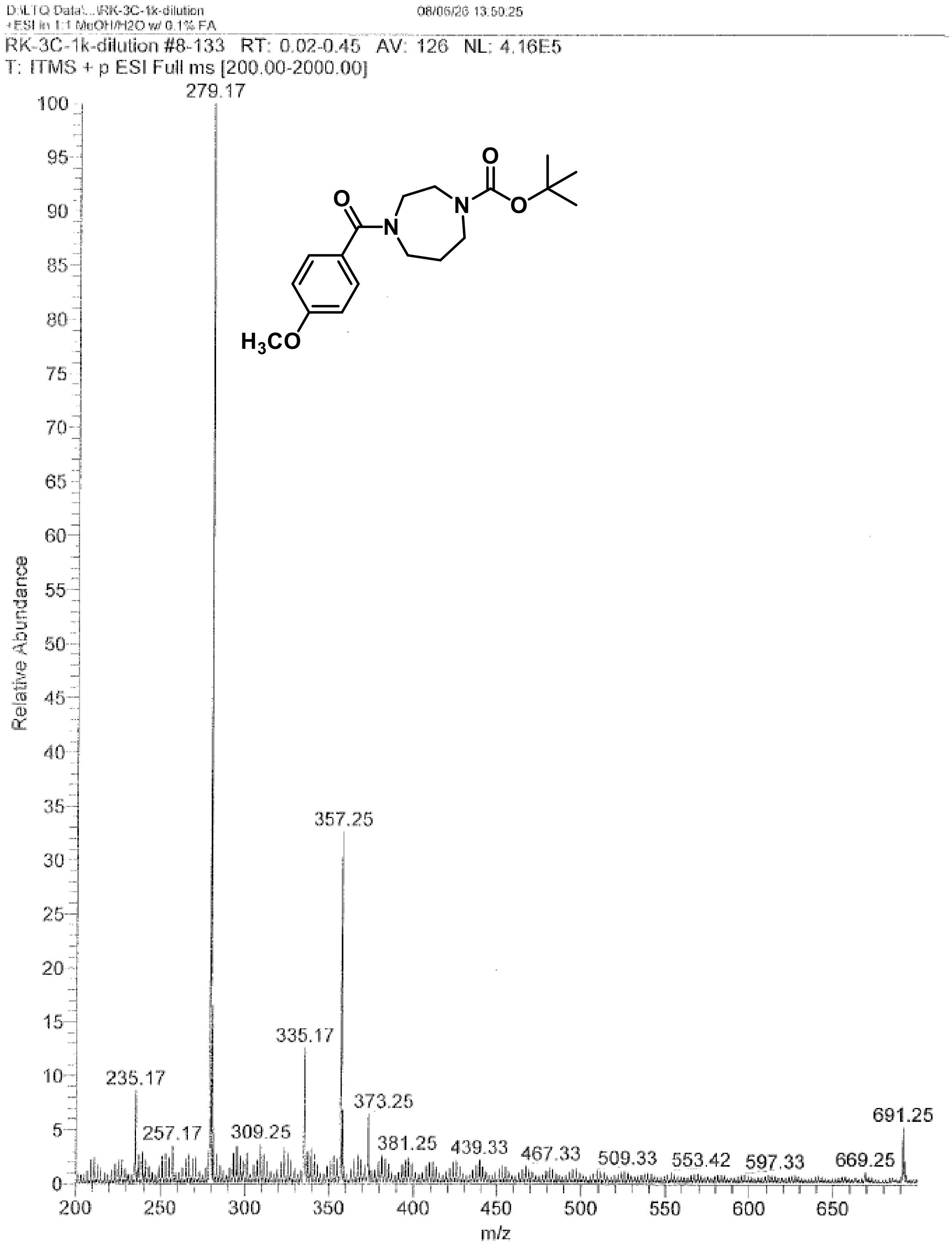
D:\LTQ Data\...\RK-3C-1k-dilution
08/06/26 13:50:25
+ESI in 1:1 MeOH/H2O w/ 0.1% FA
RK-3C-1k-dilution #8-133 RT: 0.02-0.45 AV: 126 NL: 4.16E5
T: ITMS + p ESI Full ms [200.00-2000.00]
279.17
357.25
335.17
235.17
373.25
257.17
309.25
381.25
439.33
467.33
509.33
553.42
597.33
669.25
691.25
Relative Abundance
m/z
H3CO
O
N

tert-Butyl 4-(4-nitrobenzoyl)-1,4-diazepane-1-carboxylate (3d)

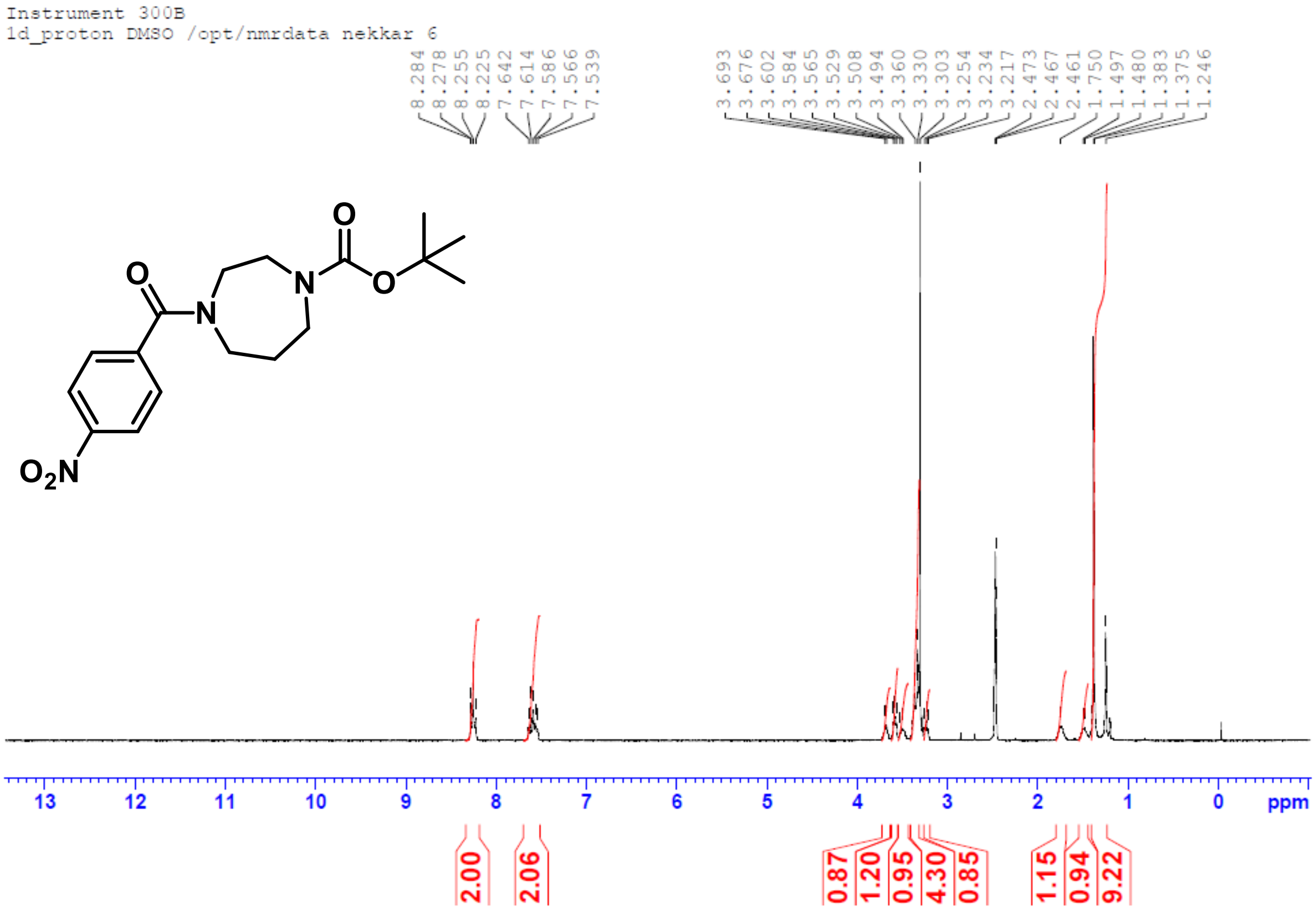

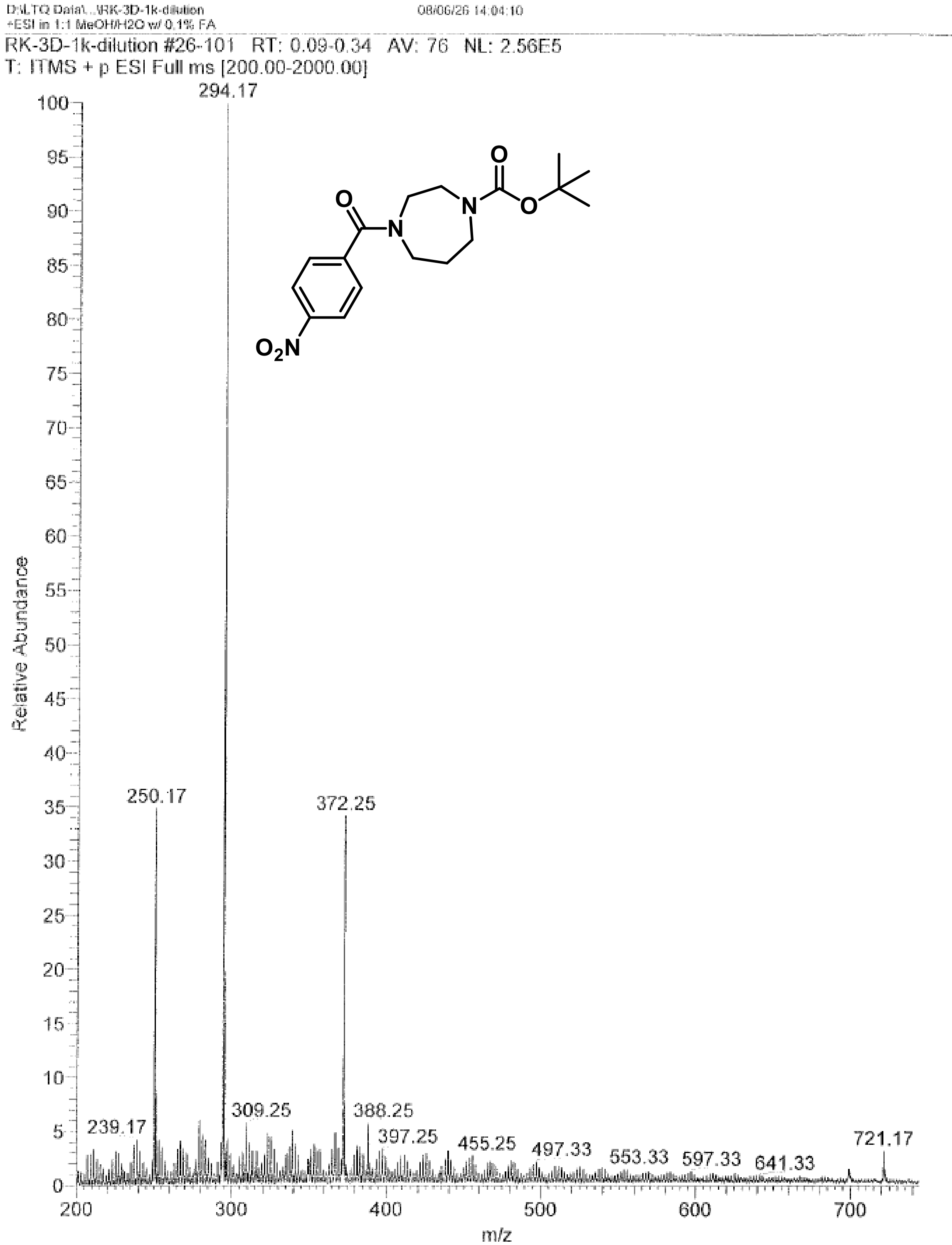
D:\LTQ Data\...\RK-3D-1k-dilution
+ESI in 1:1 MeOH/H2O w/ 0.1% FA
08/06/26 14:04:10
RK-3D-1k-dilution #26-101 RT: 0.09-0.34 AV: 76 NL: 2.56E5
T: ITMS + p ESI Full ms [200.00-2000.00]
294.17
250.17
372.25
239.17
309.25
388.25
397.25
455.25
497.33
553.33
597.33
641.33
721.17
Relative Abundance
m/z
O
N
O
O
N
O2N

***tert*-Butyl 4-(4-fluorobenzoyl)-1,4-diazepane-1-carboxylate (3e)**

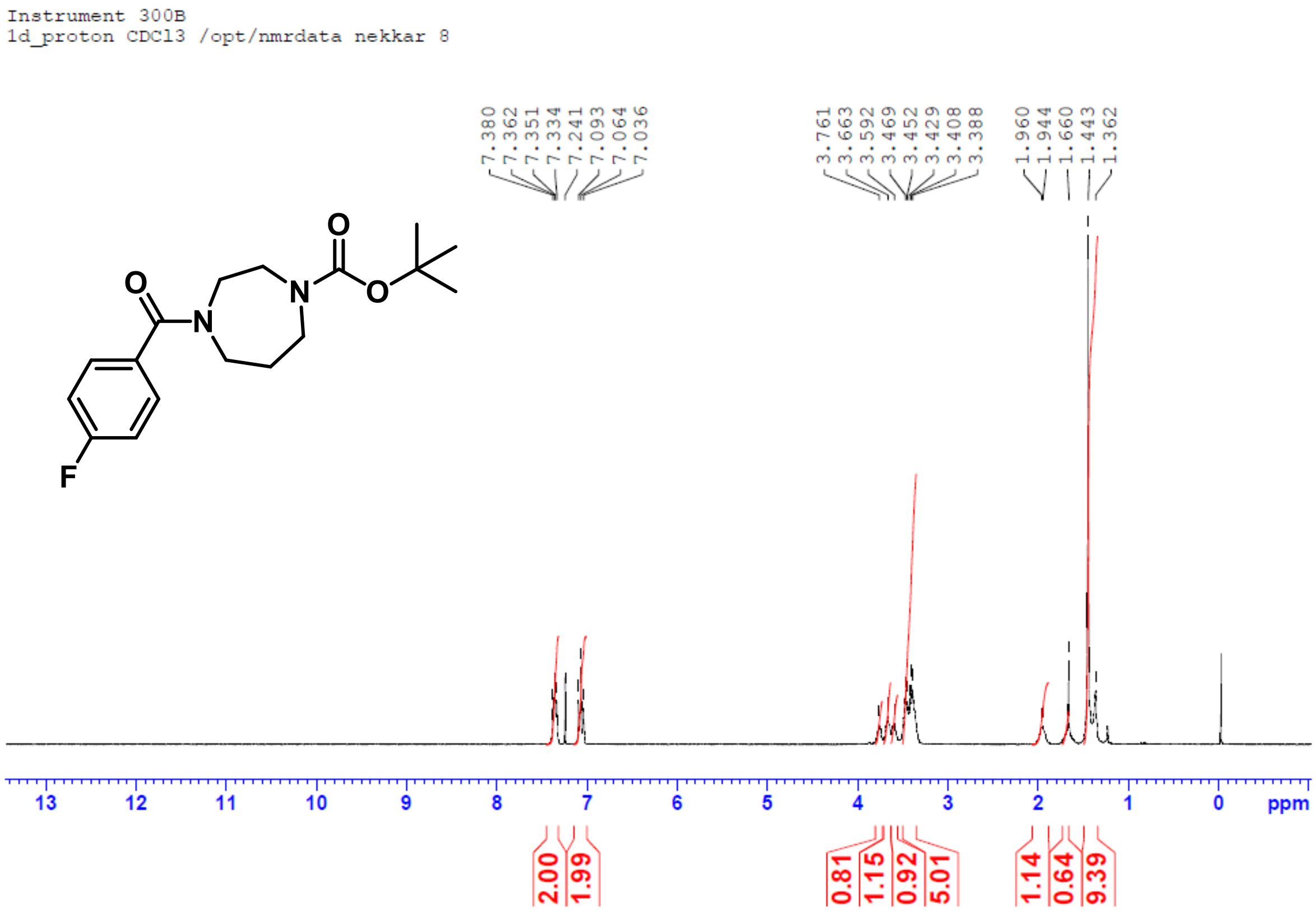

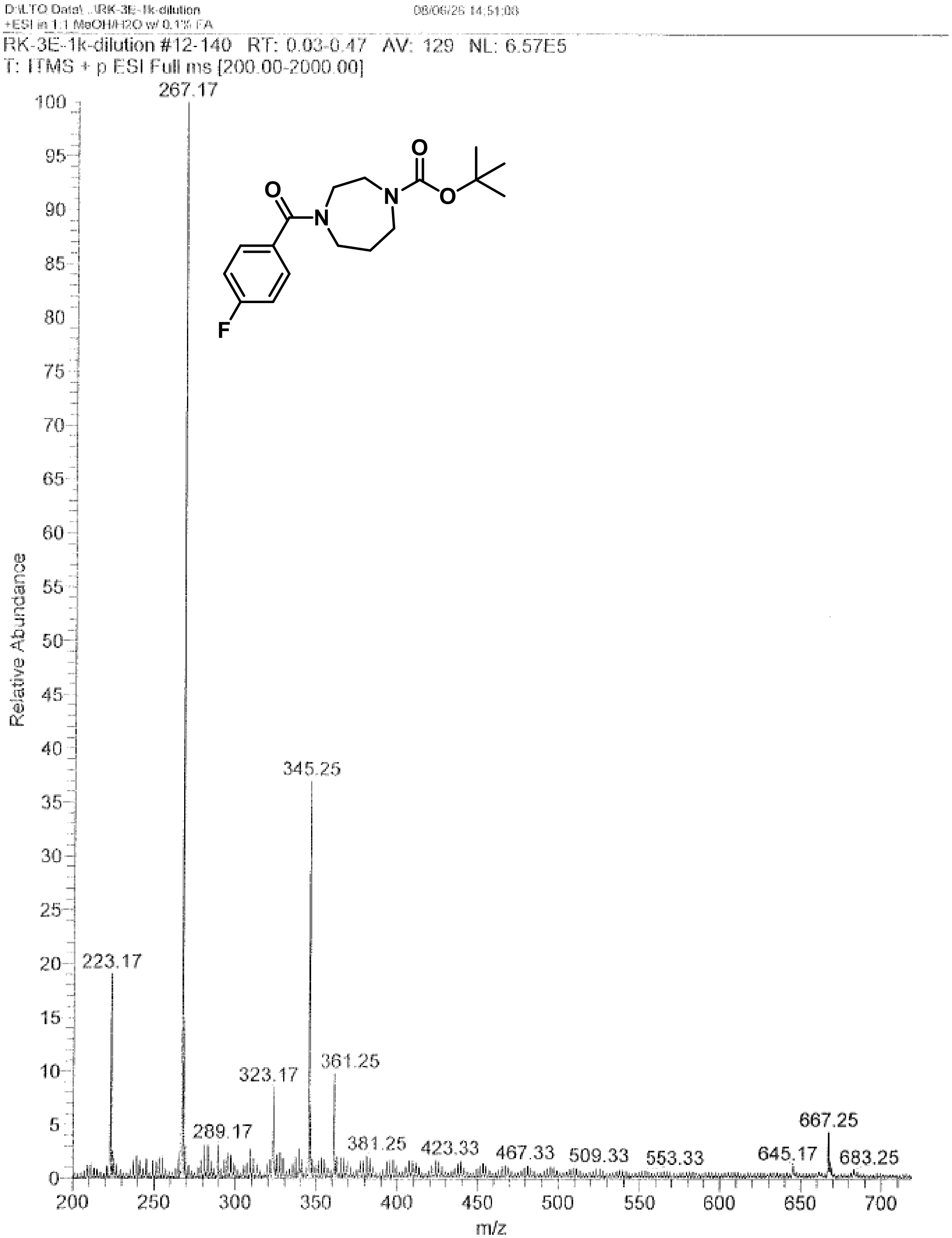
D:\LTQ Data\ ..\RK-3E-1k-dilution
+ESI in 1:1 MeOH/H2O w/ 0.1% FA
08/06/26 14:51:08
RK-3E-1k-dilution #12-140 RT: 0.03-0.47 AV: 129 NL: 6.57E5
T: ITMS + p ESI Full ms [200.00-2000.00]
267.17
345.25
223.17
323.17
361.25
289.17
381.25
423.33
467.33
509.33
553.33
645.17
667.25
683.25
Relative Abundance
m/z
O
O
N
N
O
F

tert-Butyl 4-(4-chlorobenzoyl)-1,4-diazepane-1-carboxylate (3f)

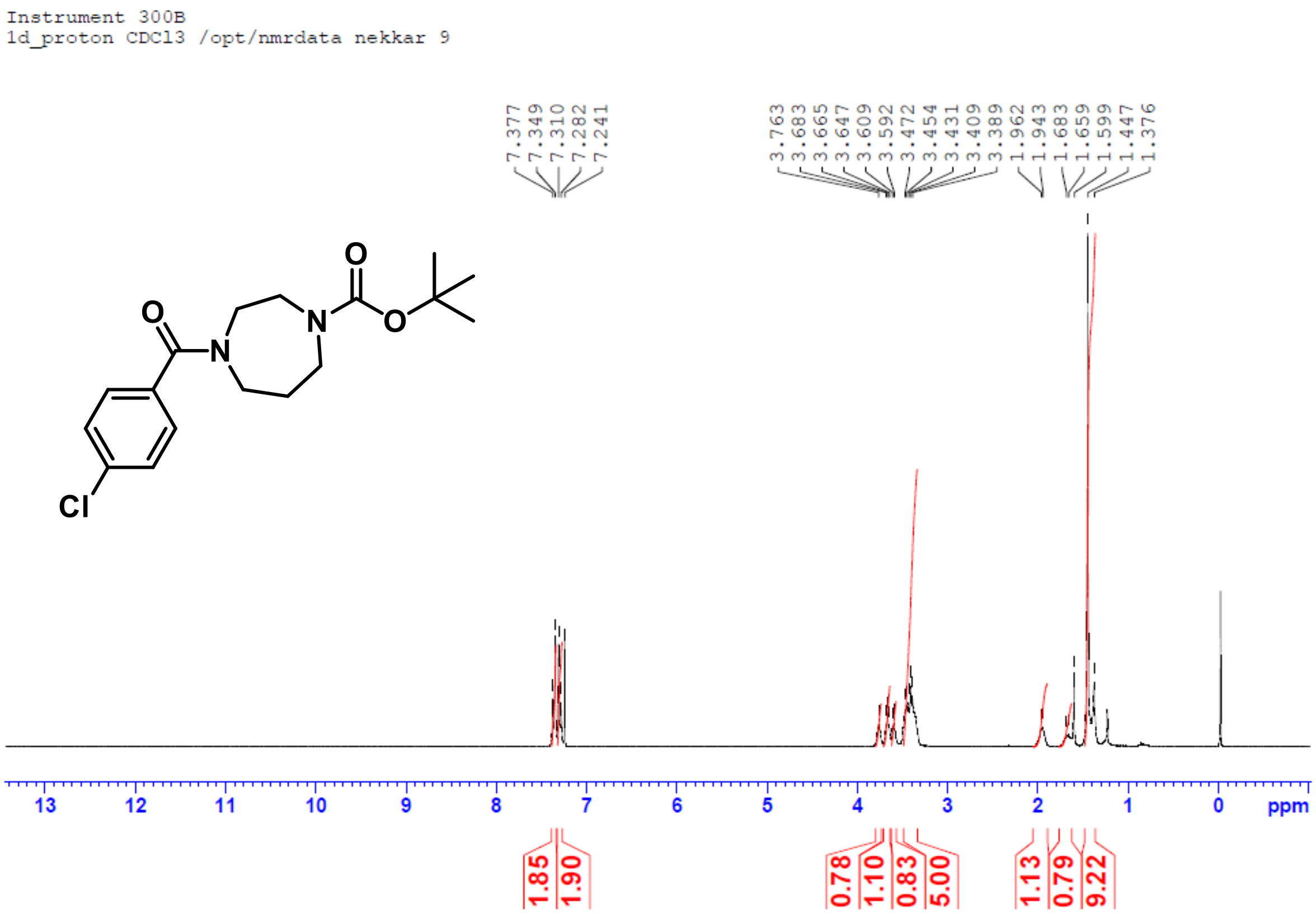

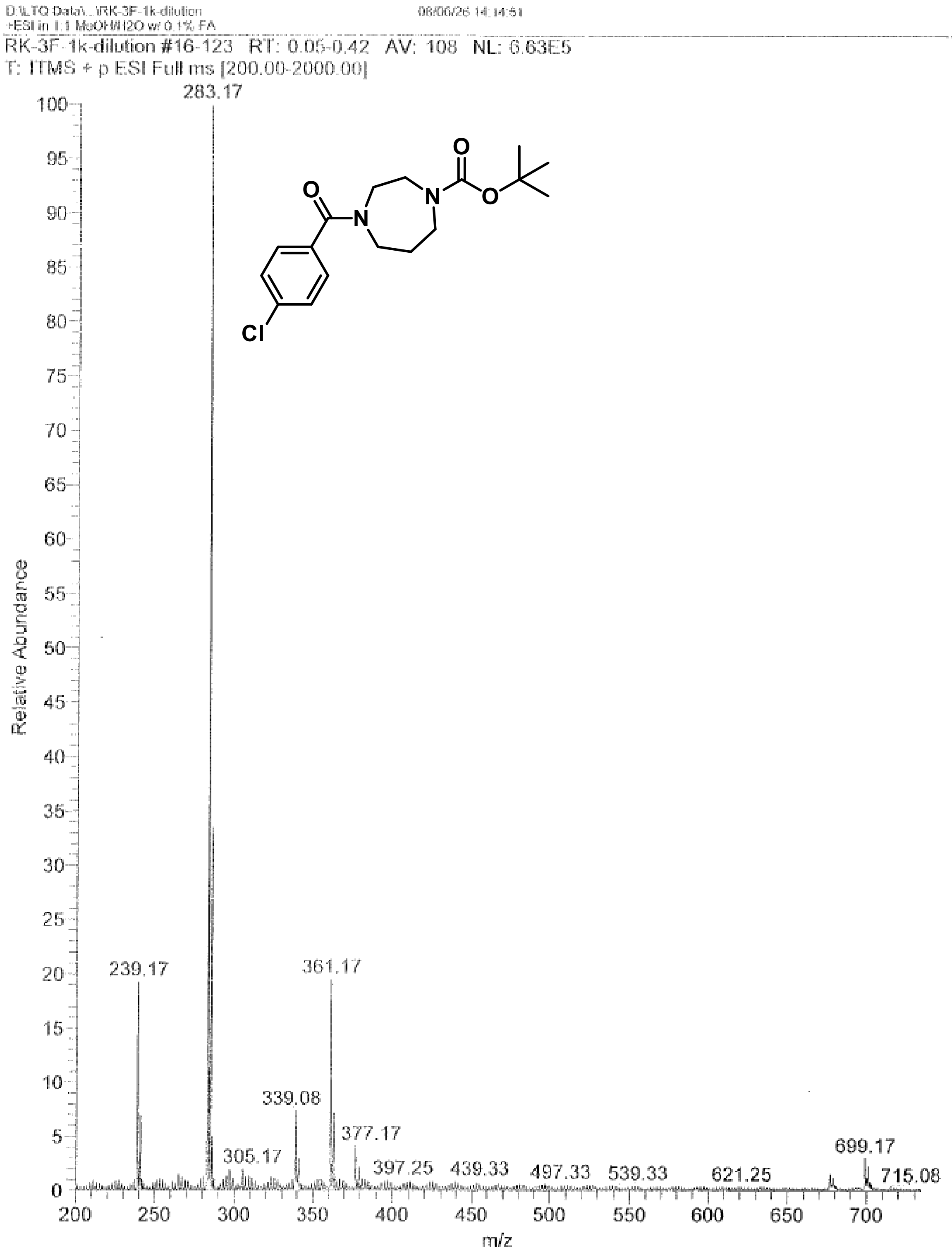
D:\LTQ Data\...\RK-3F-1k-dilution
+ESI in 1:1 MeOH/H2O w/ 0.1% FA
08/06/26 14:14:51
RK-3F-1k-dilution #16-123 RT: 0.05-0.42 AV: 108 NL: 6.63E5
T: ITMS + p ESI Full ms [200.00-2000.00]
283.17
239.17
361.17
339.08
377.17
305.17
397.25
439.33
497.33
539.33
621.25
699.17
715.08
Relative Abundance
m/z
O
N
N
O
O
Cl

tert-Butyl 4-(4-bromobenzoyl)-1,4-diazepane-1-carboxylate (3g)

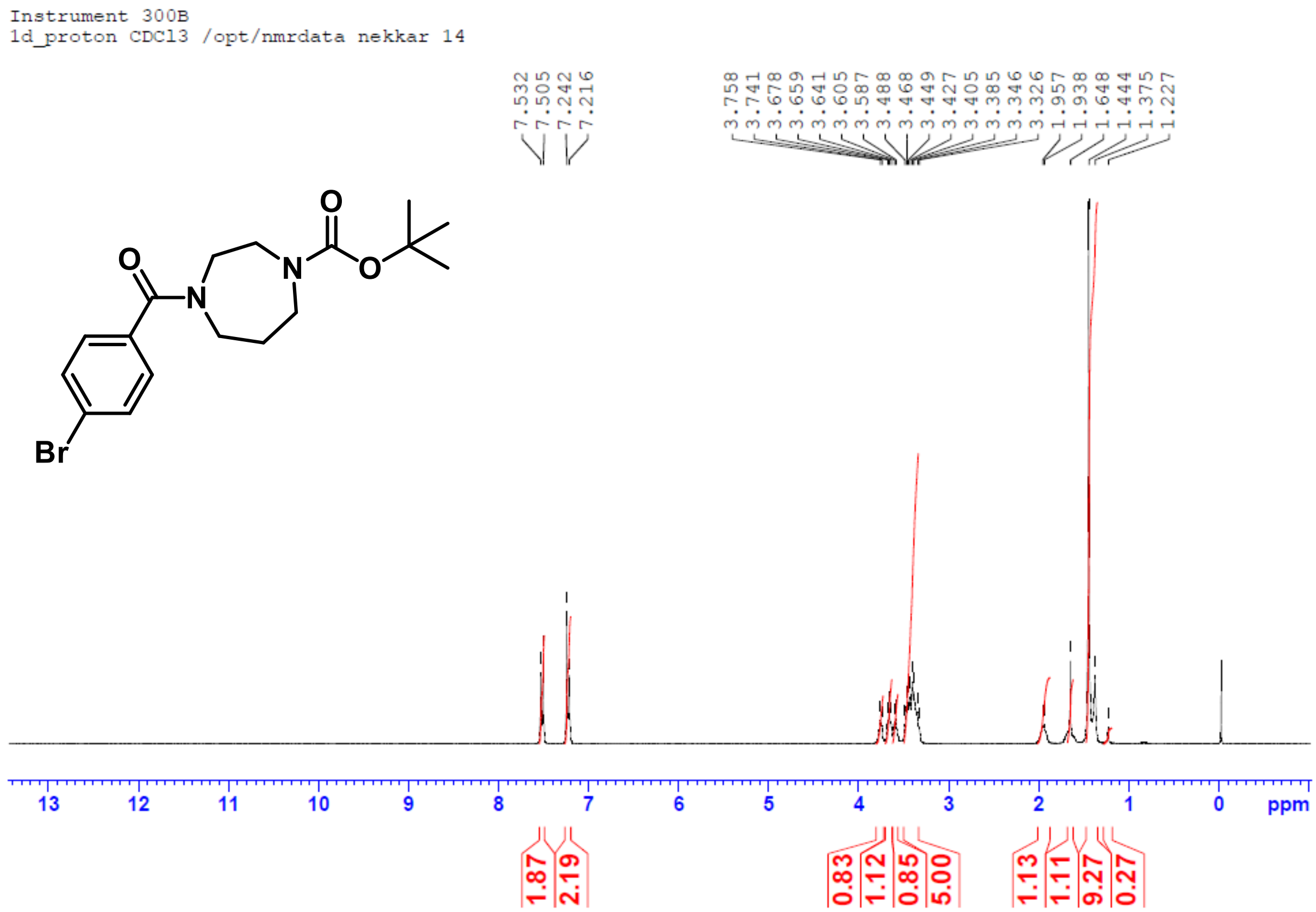

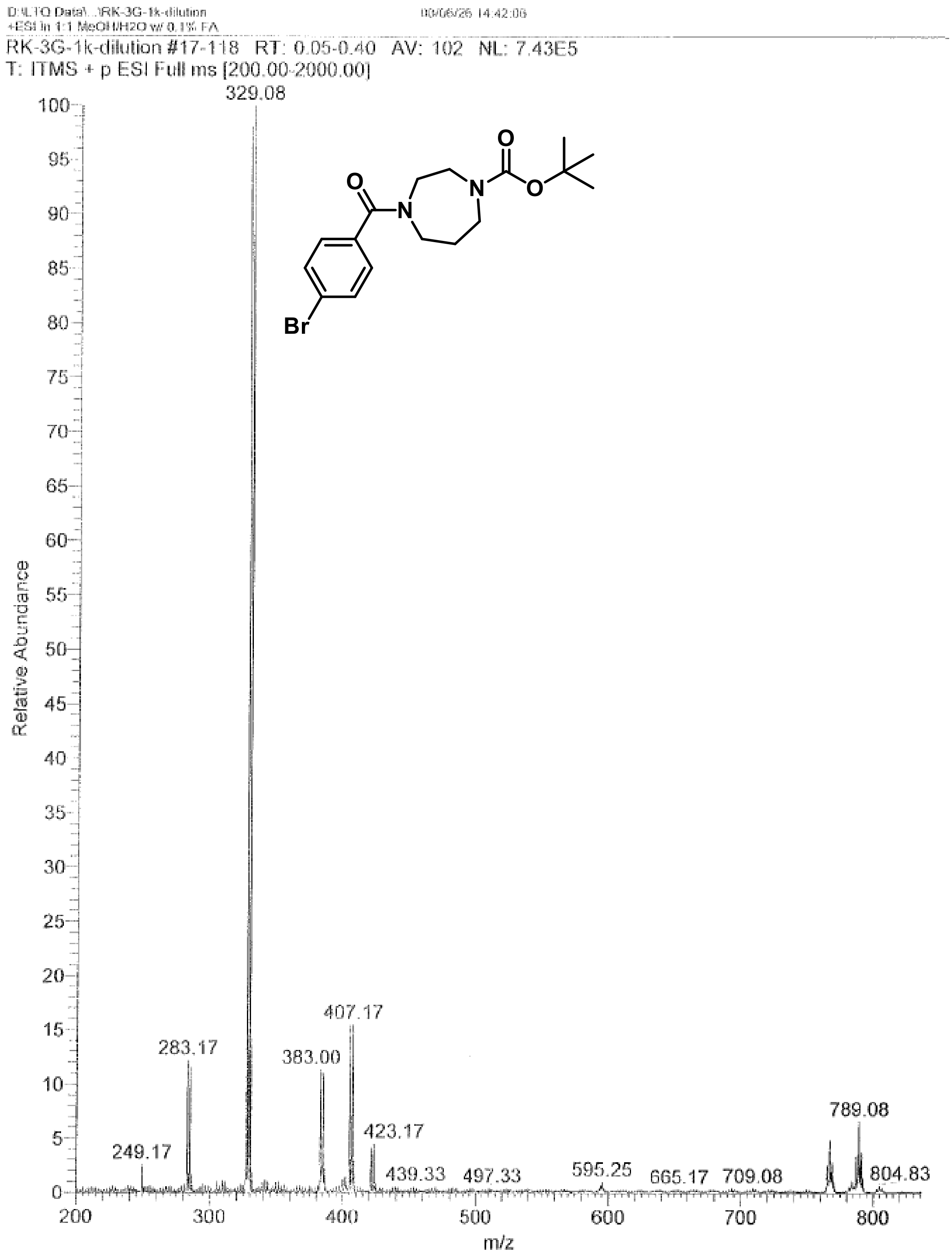
D:\LTQ Data\...\RK-3G-1k-dilution
+ESI in 1:1 MeOH/H2O w/ 0.1% FA
09/06/26 14:42:06
RK-3G-1k-dilution #17-118 RT: 0.05-0.40 AV: 102 NL: 7.43E5
T: ITMS + p ESI Full ms [200.00-2000.00]
329.08
407.17
283.17
383.00
423.17
249.17
439.33
497.33
595.25
665.17
709.08
789.08
804.83
Relative Abundance
m/z
O
N
N
O
O
Br

***tert*-Butyl 4-(4-iodobenzoyl)-1,4-diazepane-1-carboxylate (3h)**

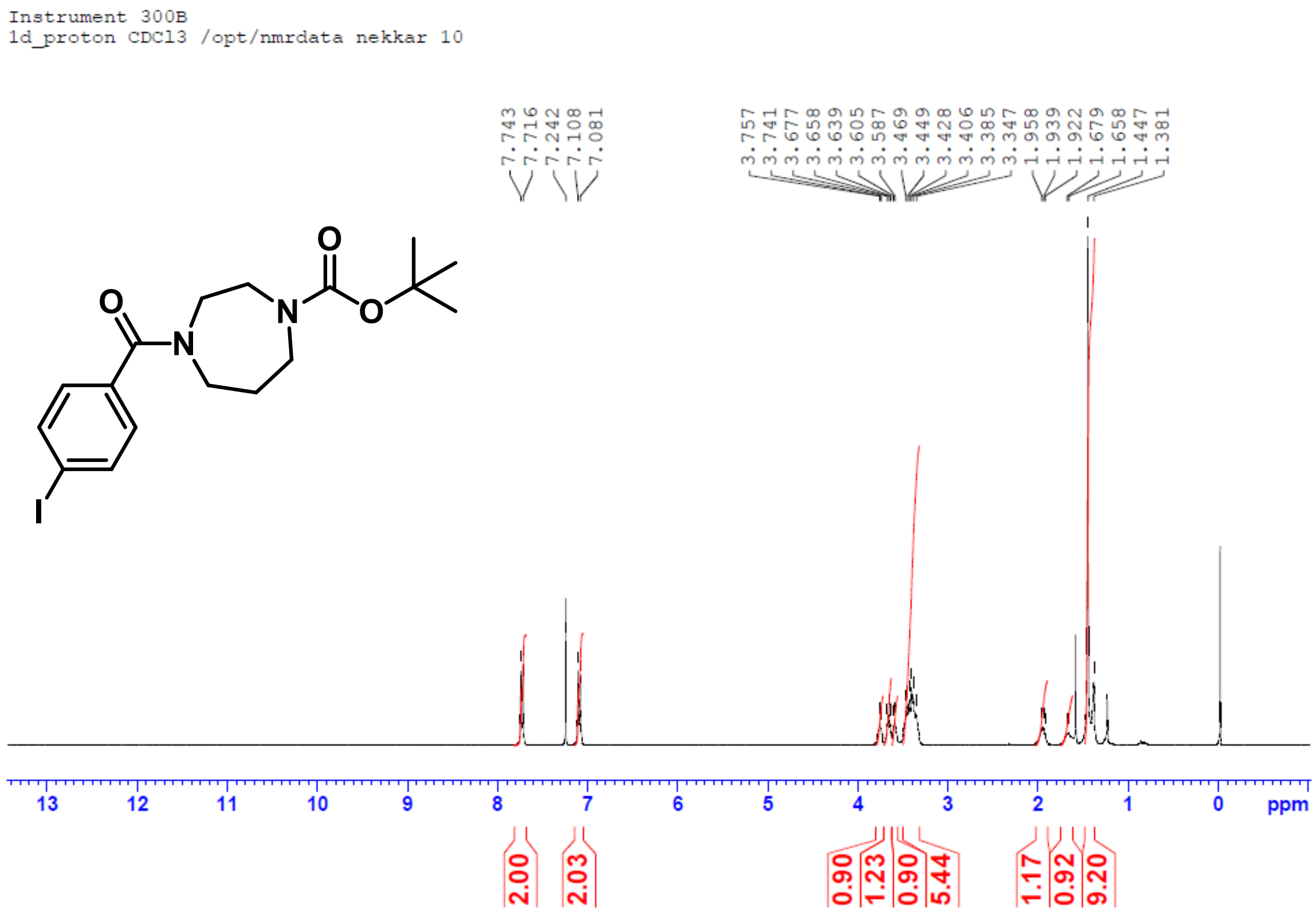

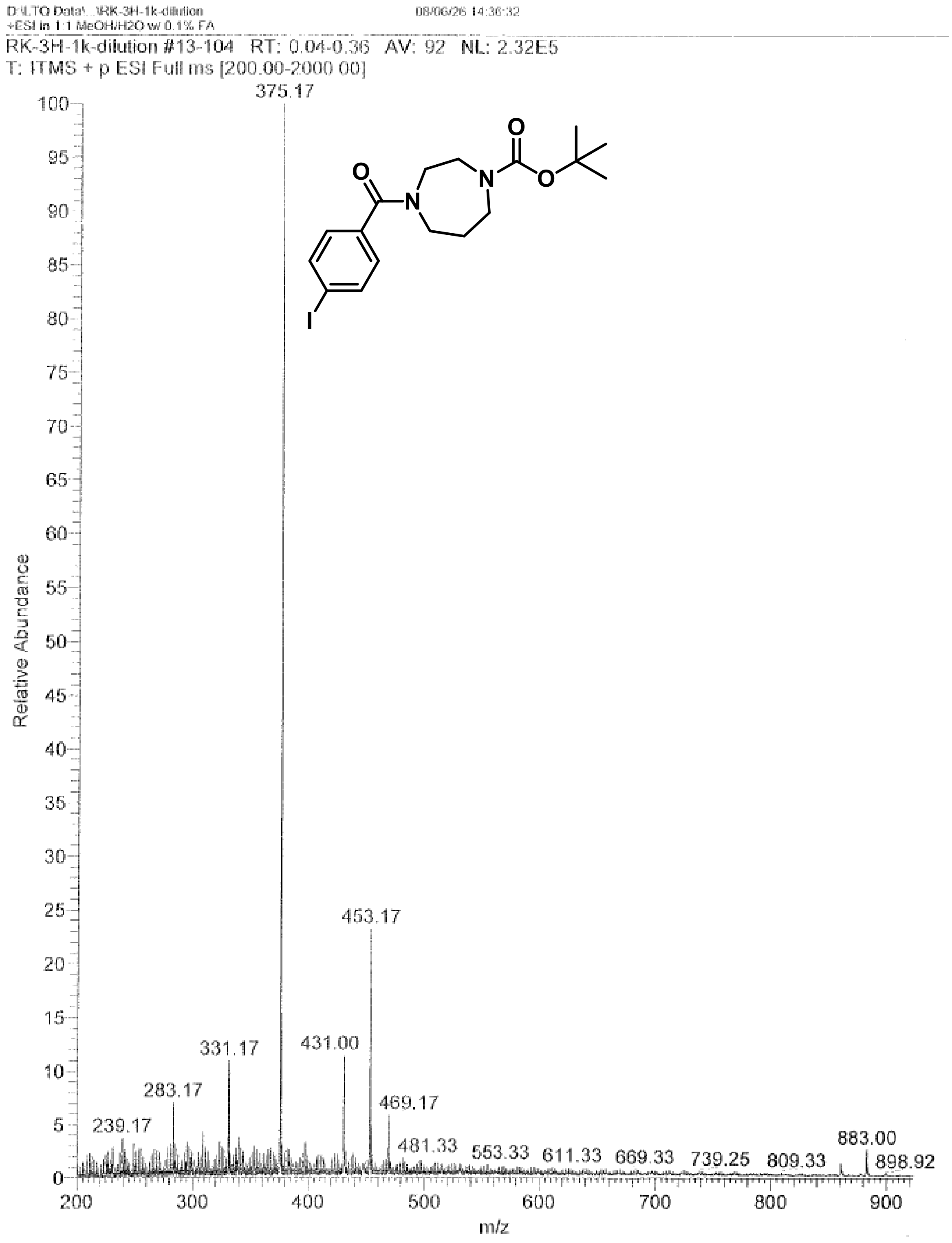
D:\LTQ Data\...\RK-3H-1k-dilution
+ESI in 1:1 MeOH/H2O w/ 0.1% FA
08/06/26 14:36:32
RK-3H-1k-dilution #13-104 RT: 0.04-0.36 AV: 92 NL: 2.32E5
T: ITMS + p ESI Full ms [200.00-2000.00]
375.17
453.17
431.00
331.17
283.17
469.17
239.17
481.33
553.33
611.33
669.33
739.25
809.33
883.00
898.92
Relative Abundance
m/z

***tert*-Butyl 4-(3,4-dimethoxybenzoyl)-1,4-diazepane-1-carboxylate (3i)**

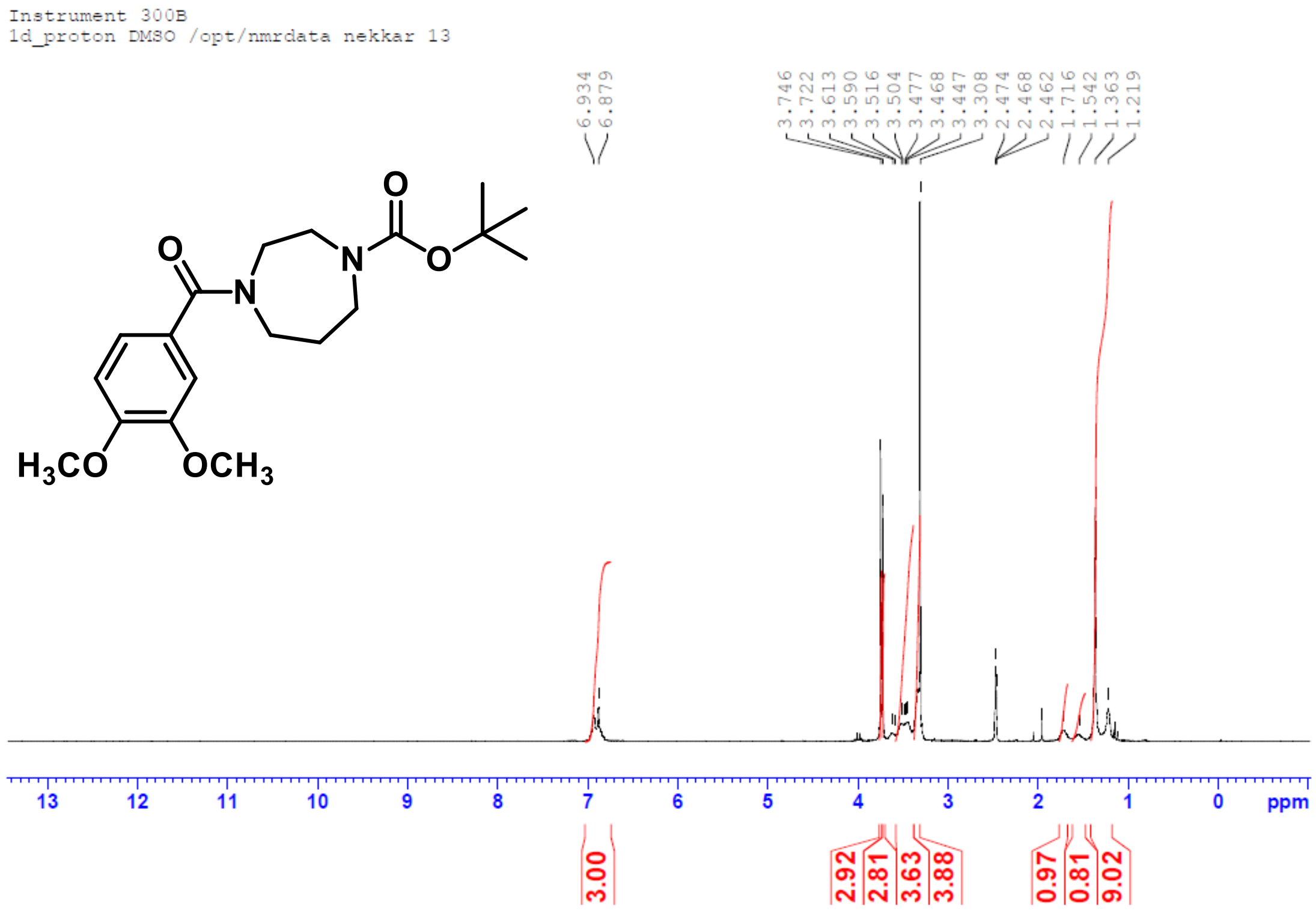

***tert*-Butyl 4-(3,4-difluorobenzoyl)-1,4-diazepane-1-carboxylate (3j)**

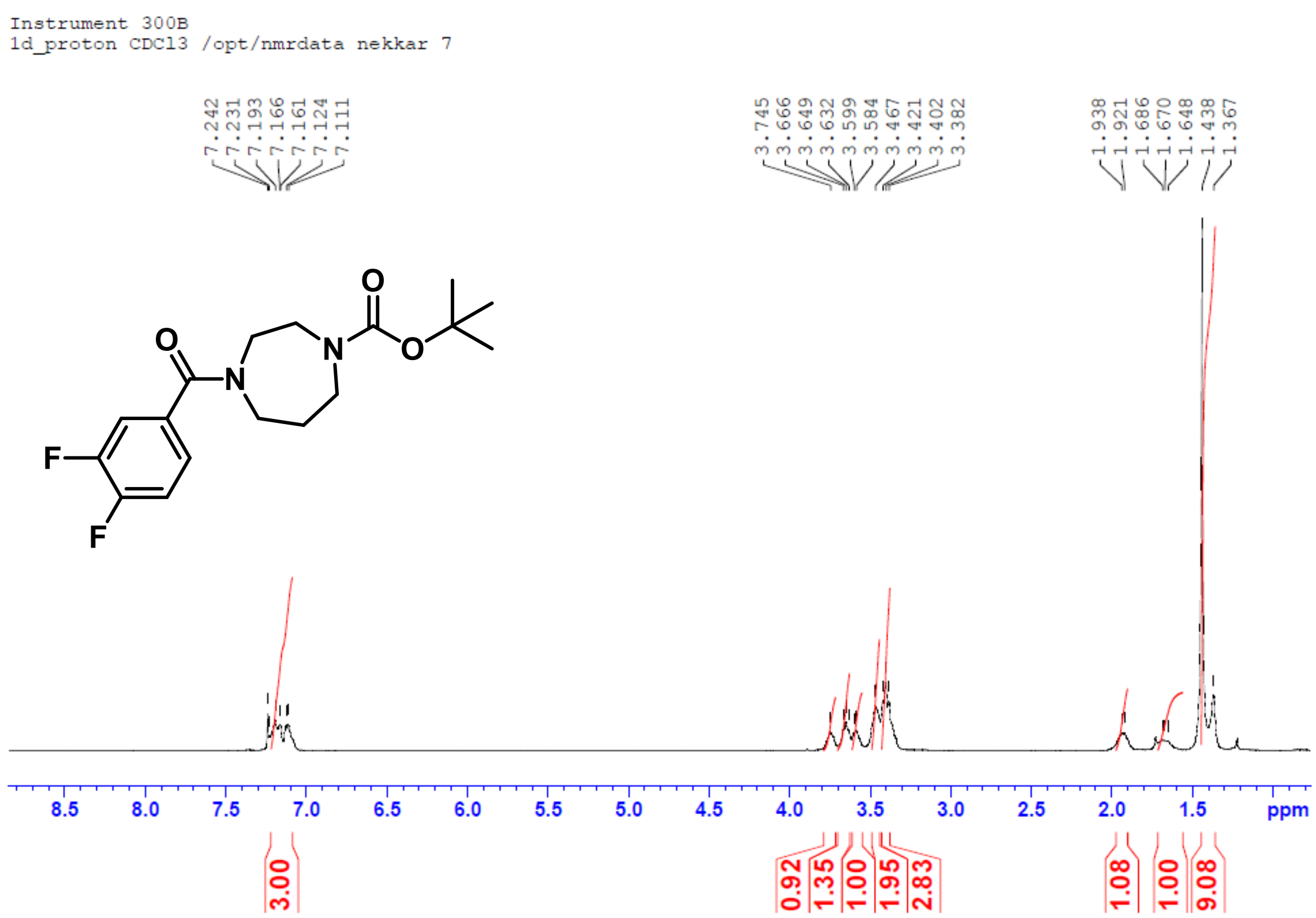

***tert*-Butyl 4-(4-hydroxy-3-methoxybenzoyl)-1,4-diazepane-1-carboxylate (3k)**

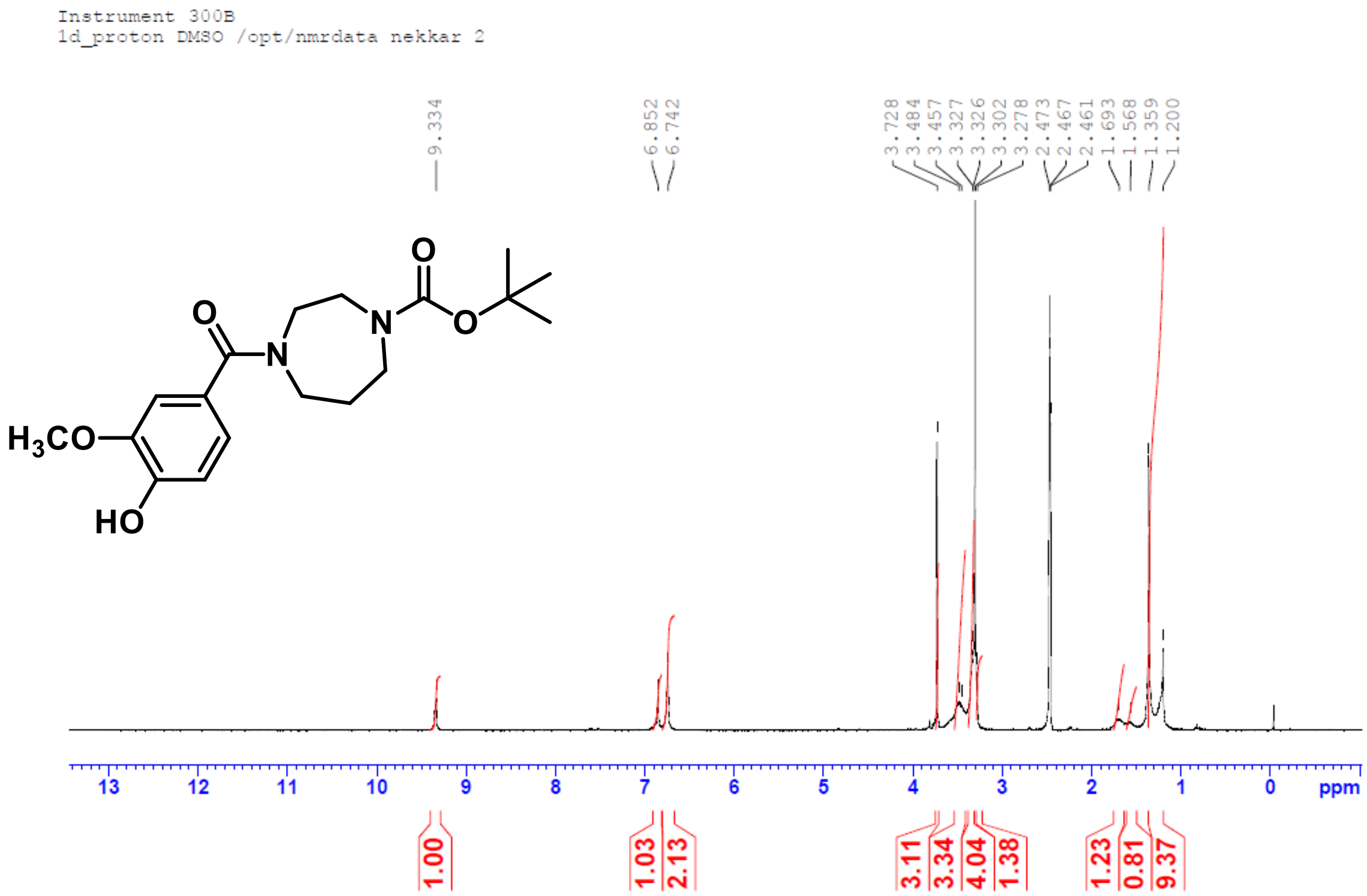

***tert*-Butyl 4-(3,4-dihydroxybenzoyl)-1,4-diazepane-1-carboxylate (3l)**

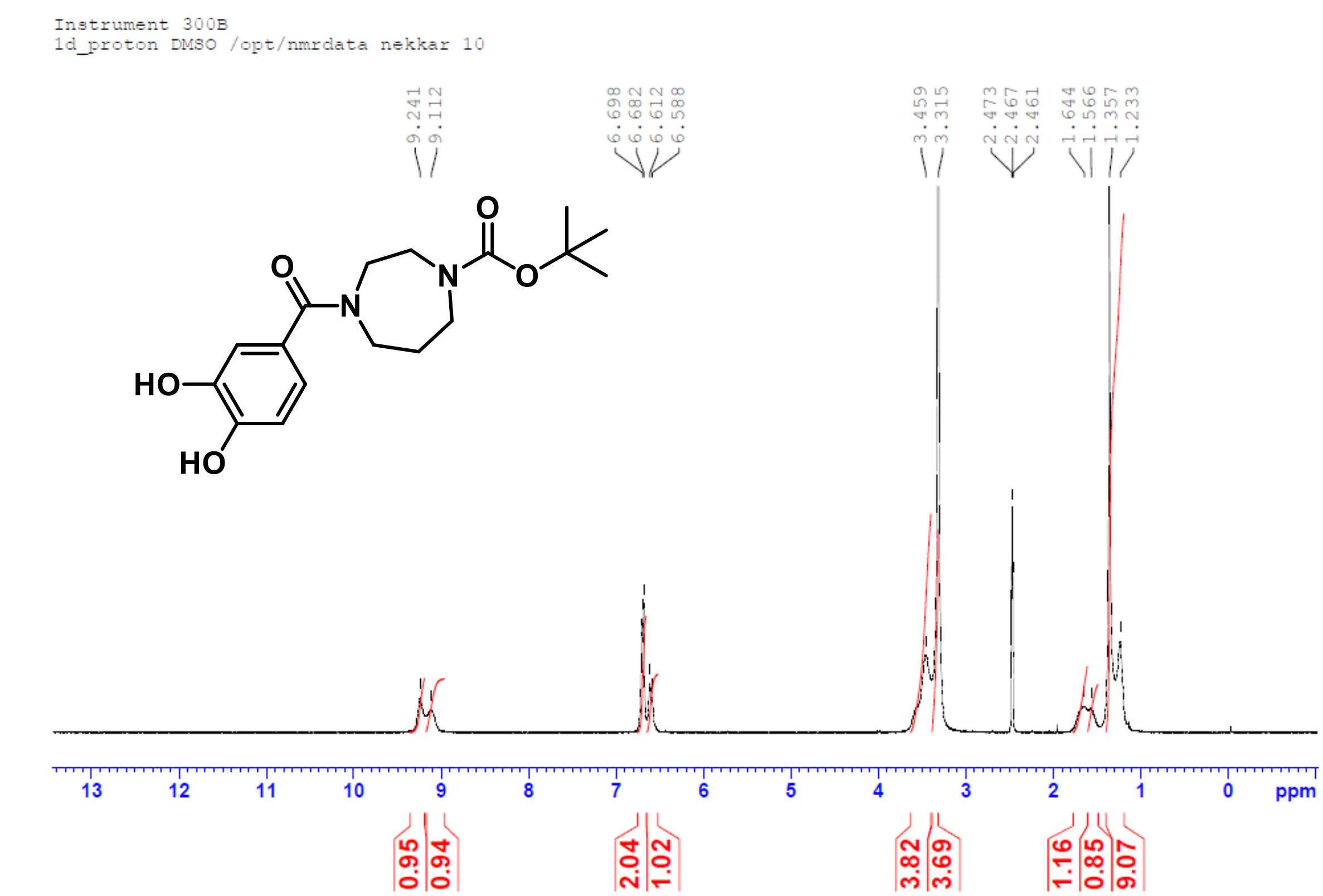

tert-Butyl 4-(benzo[_d_][1,3]dioxole-5-carbonyl)-1,4-diazepane-1-carboxylate (3m)

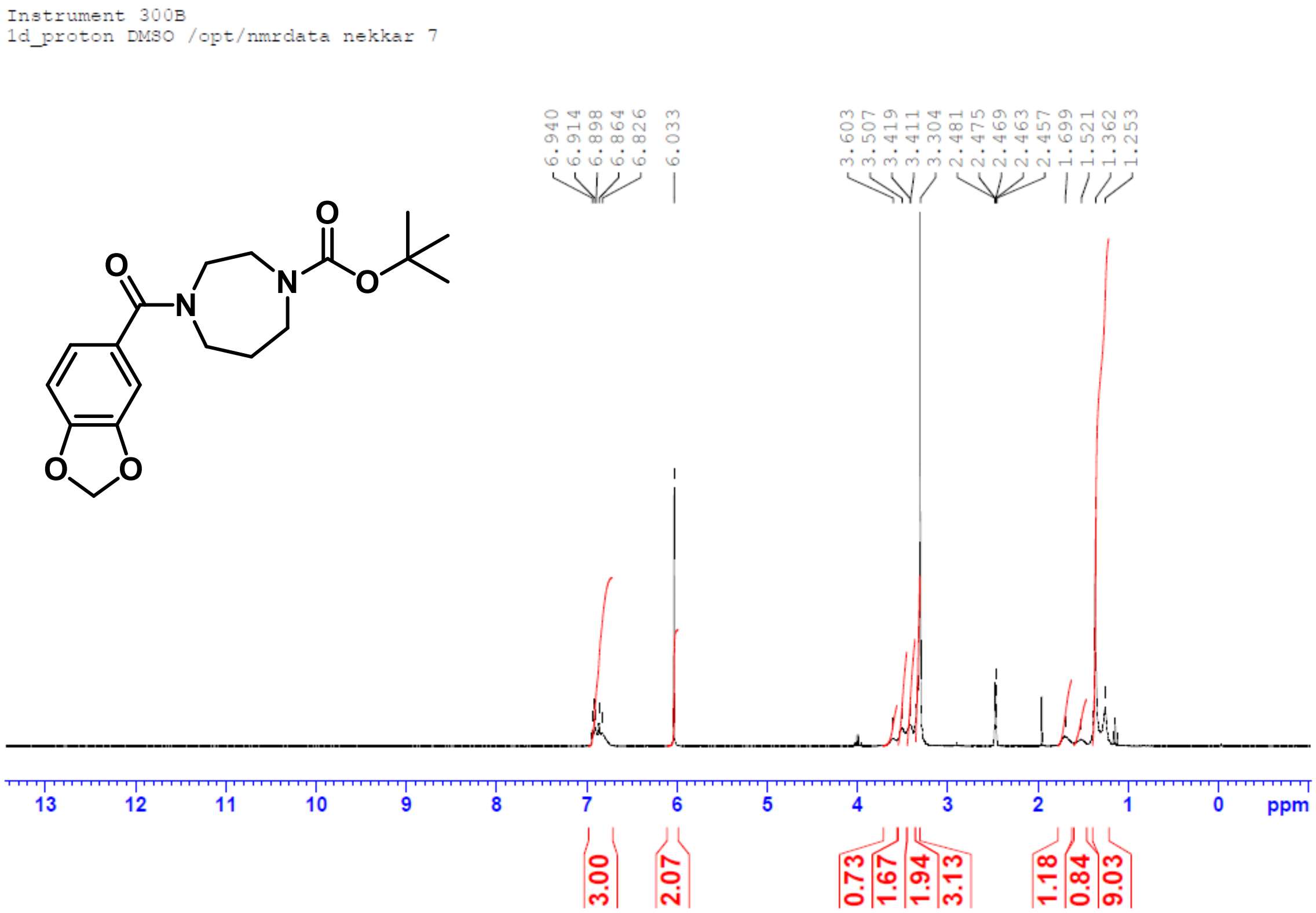

2 Analytical data (^{1}H NMR, ^{13}C NMR, LCMS, UPLC, and HRMS) for compounds 4a–n

(1,4-Diazepan-1-yl)(phenyl)methanone (4a)

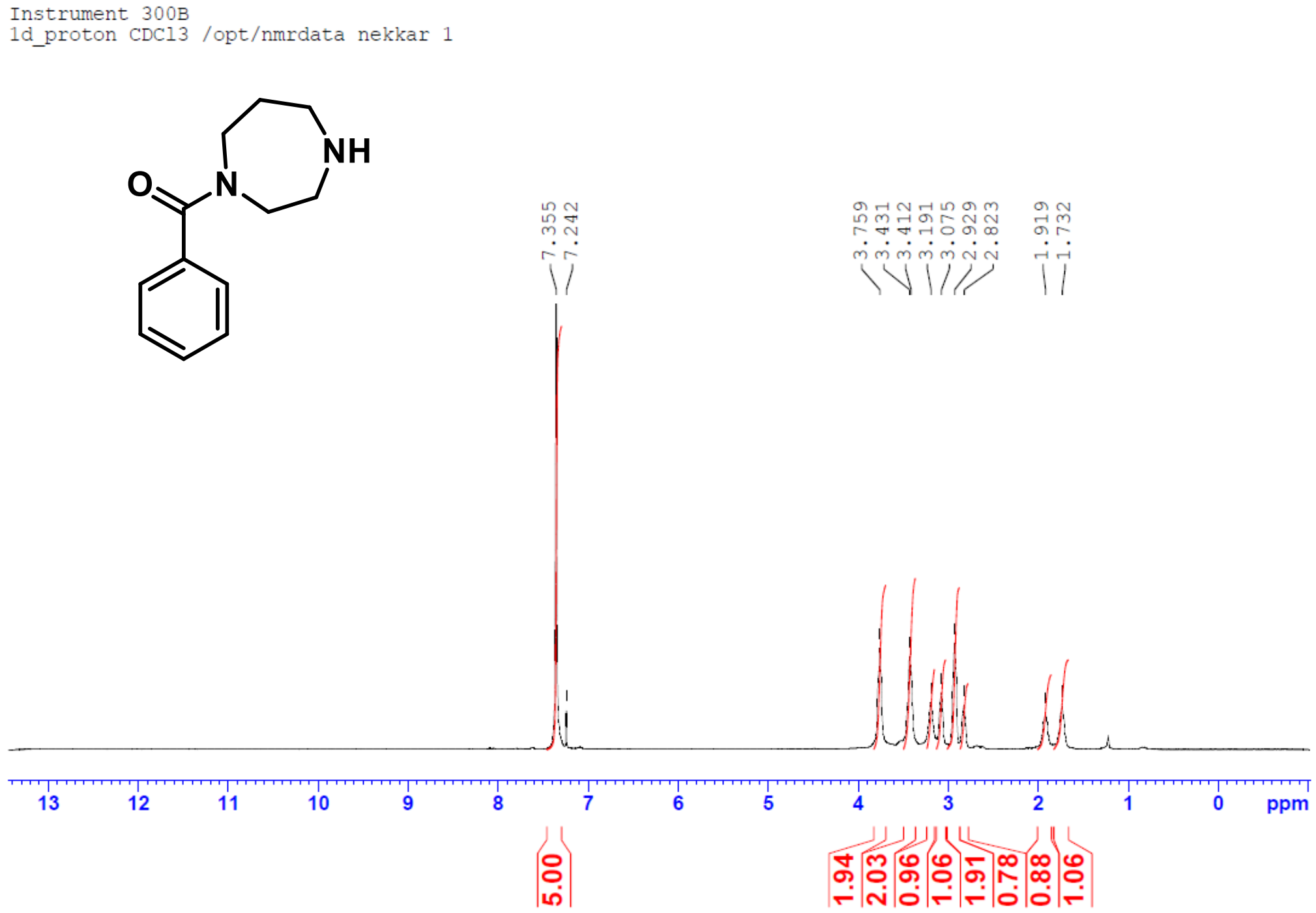

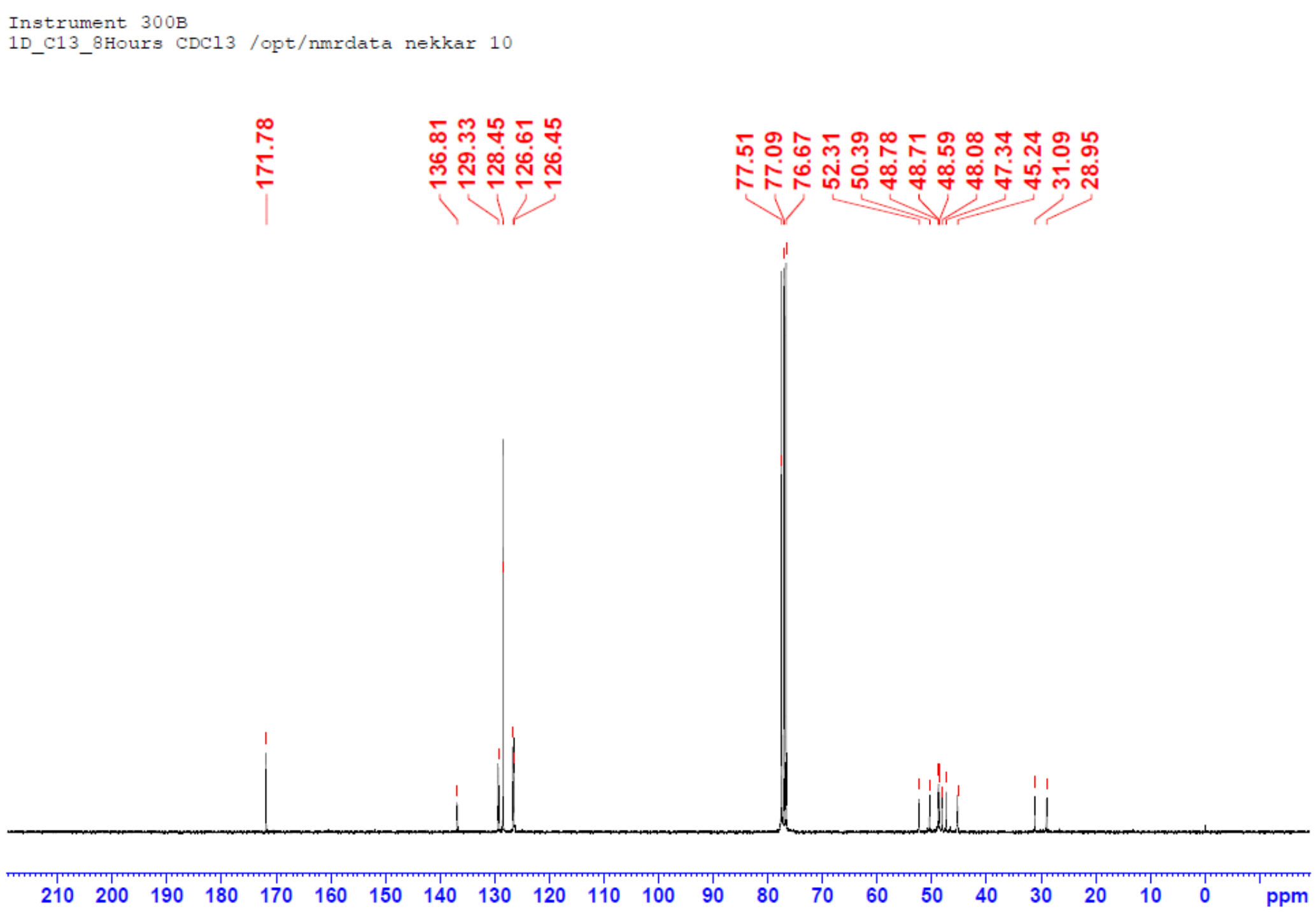

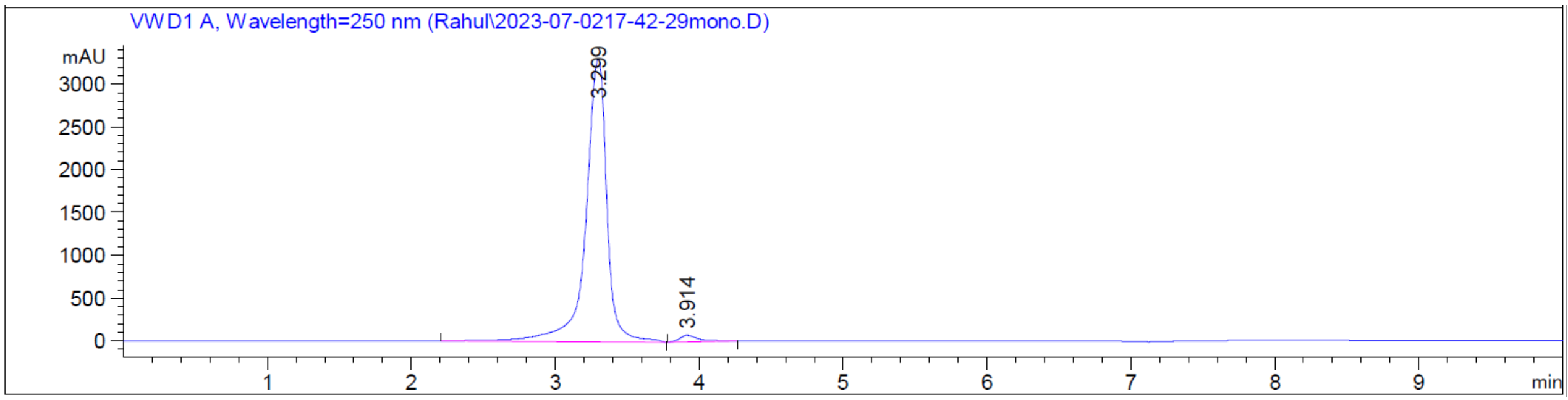

Signal 3: VWD1 A, Wavelength=250 nm

Peak #	RetTime [min]	Type	Width [min]	Area [mAU*s]	Height [mAU]	Area %
1	3.299	BB	0.1535	3.38335e4	3303.57178	98.0627
2	3.914	BB	0.1283	668.40906	76.24219	1.9373

Totals : 3.45019e4 3379.81396

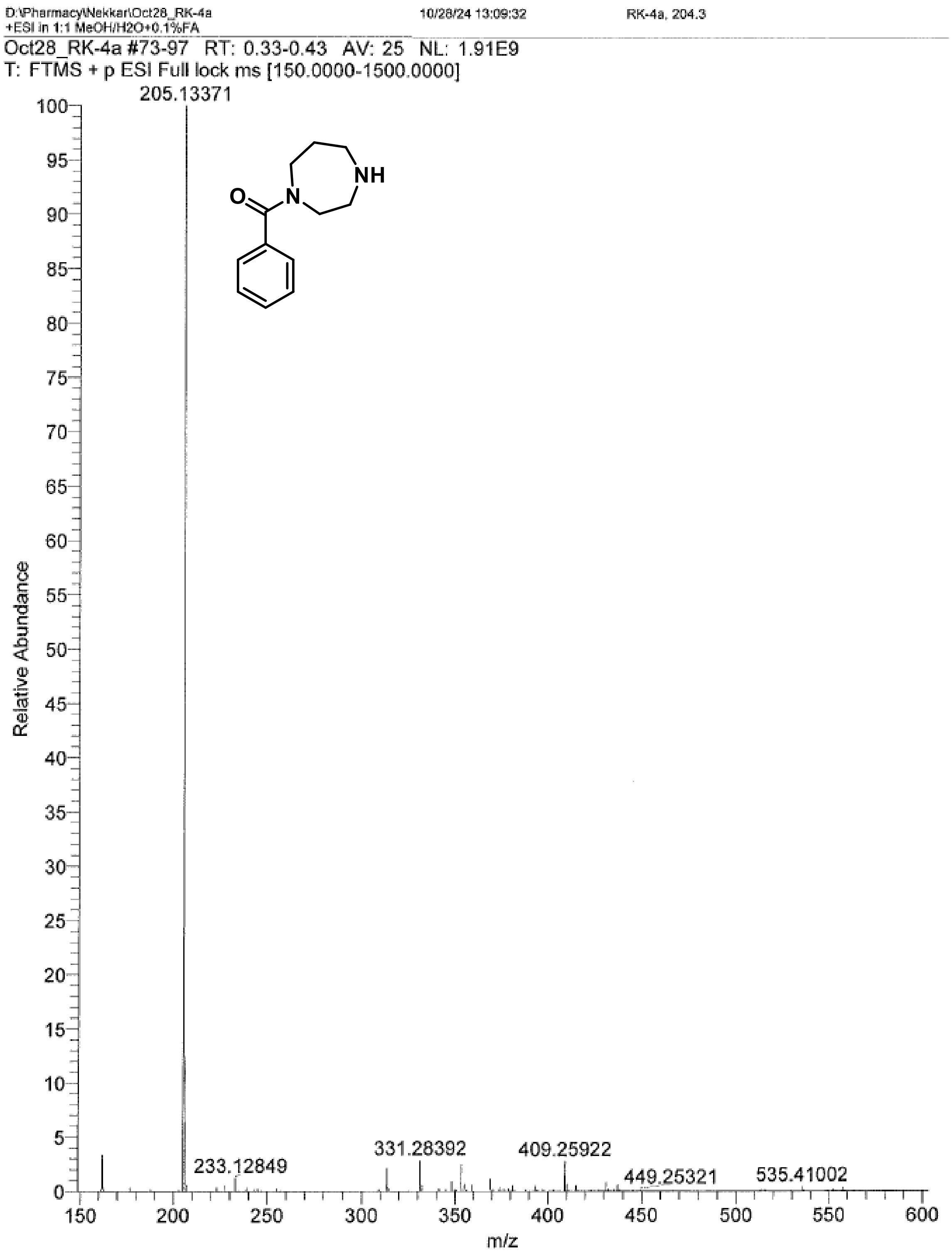
D:\Pharmacy\Nekkar\Oct28_RK-4a
+ESI in 1:1 MeOH/H2O+0.1%FA
10/28/24 13:09:32
RK-4a, 204.3
Oct28_RK-4a #73-97 RT: 0.33-0.43 AV: 25 NL: 1.91E9
T: FTMS + p ESI Full lock ms [150.0000-1500.0000]
205.13371
233.12849
331.28392
409.25922
449.25321
535.41002
O
N
NH
Relative Abundance
m/z

(1,4-Diazepan-1-yl)(*p*-tolyl)methanone (4b)

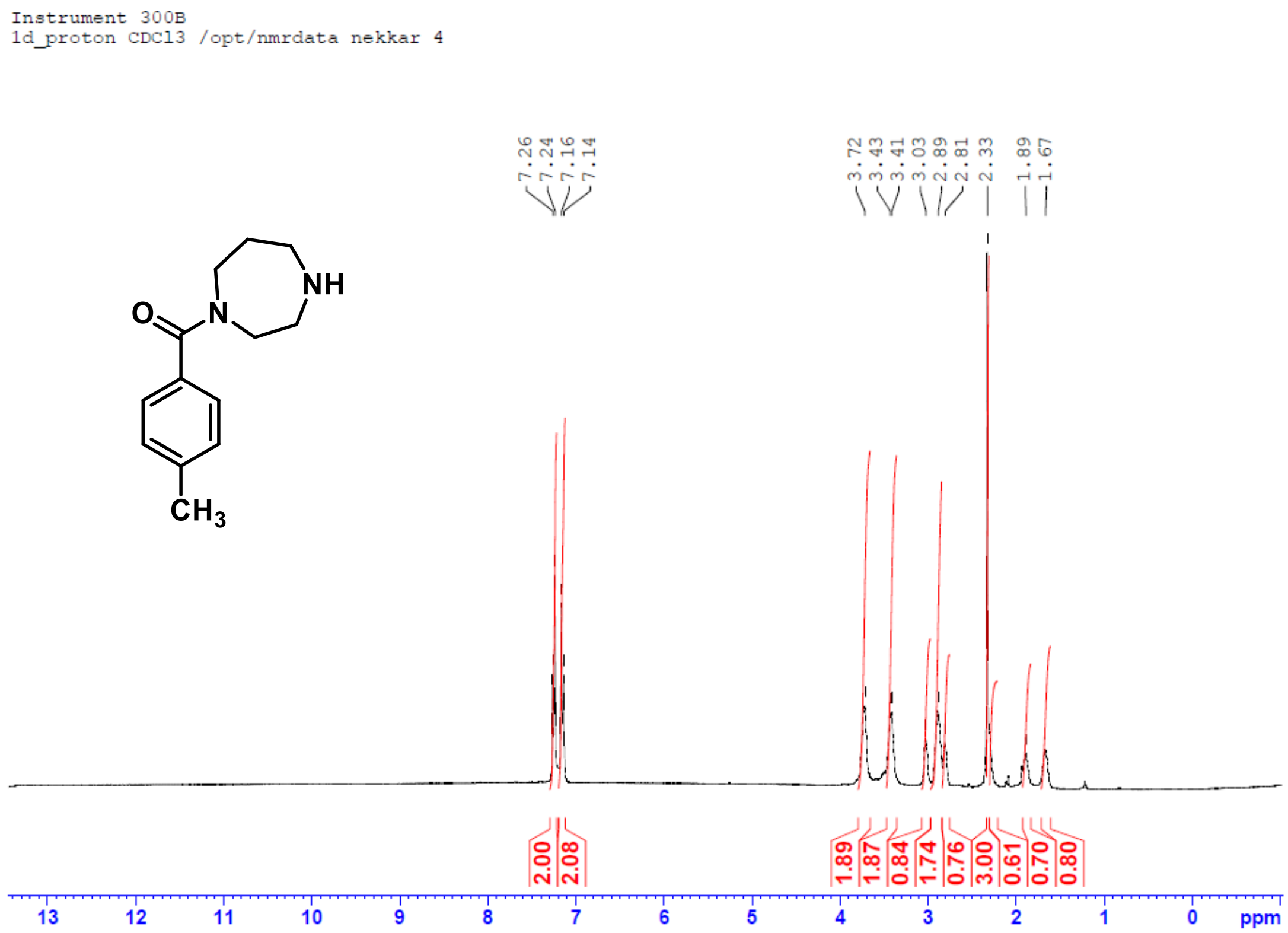

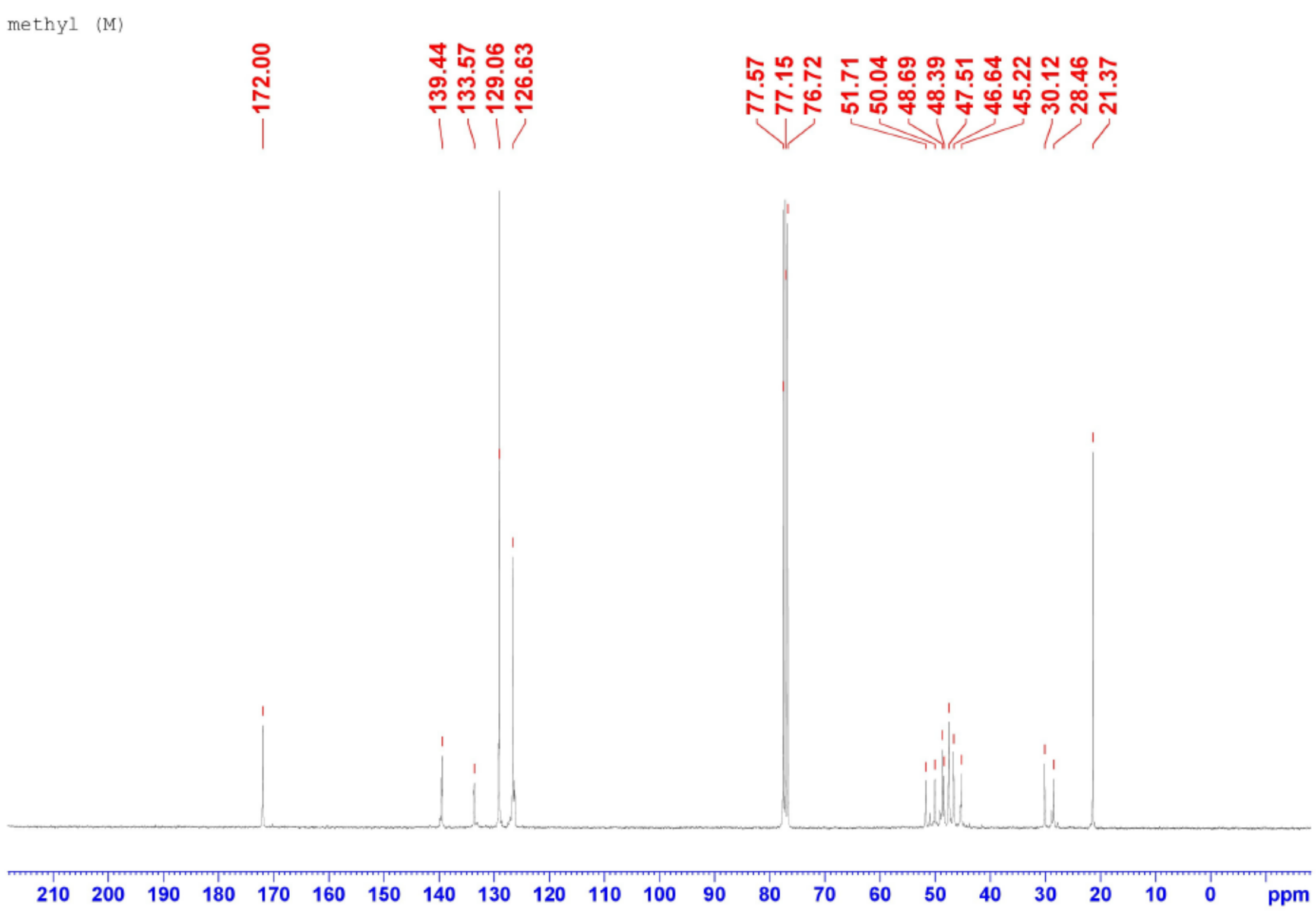

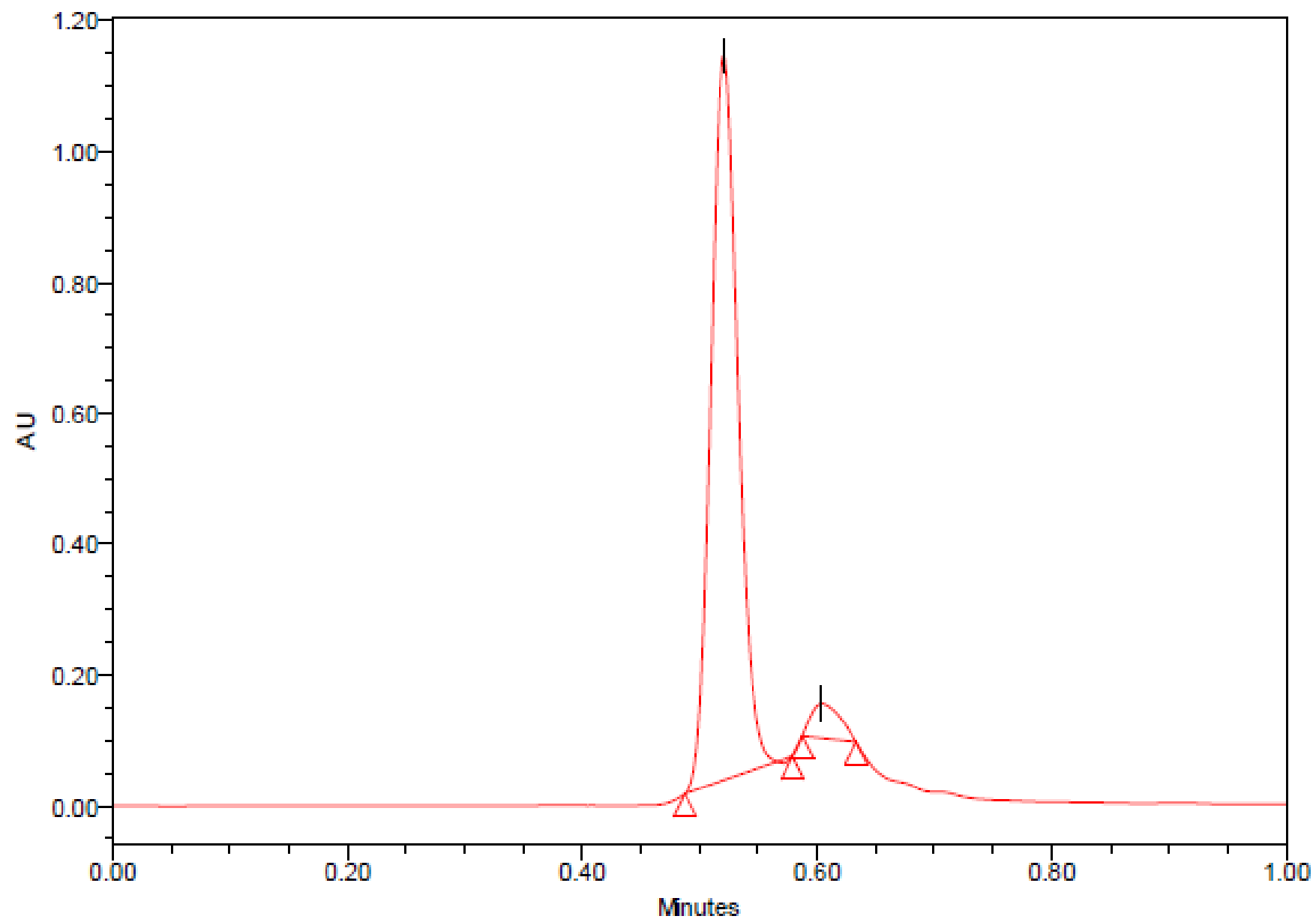

Peak Name:

	Injection	RT	Area	% Area
1	1	0.604	91065	4.86
2	1	0.521	1781620	95.14
Mean		0.562		
Std. Dev.		0.059		
% RSD		10.52		

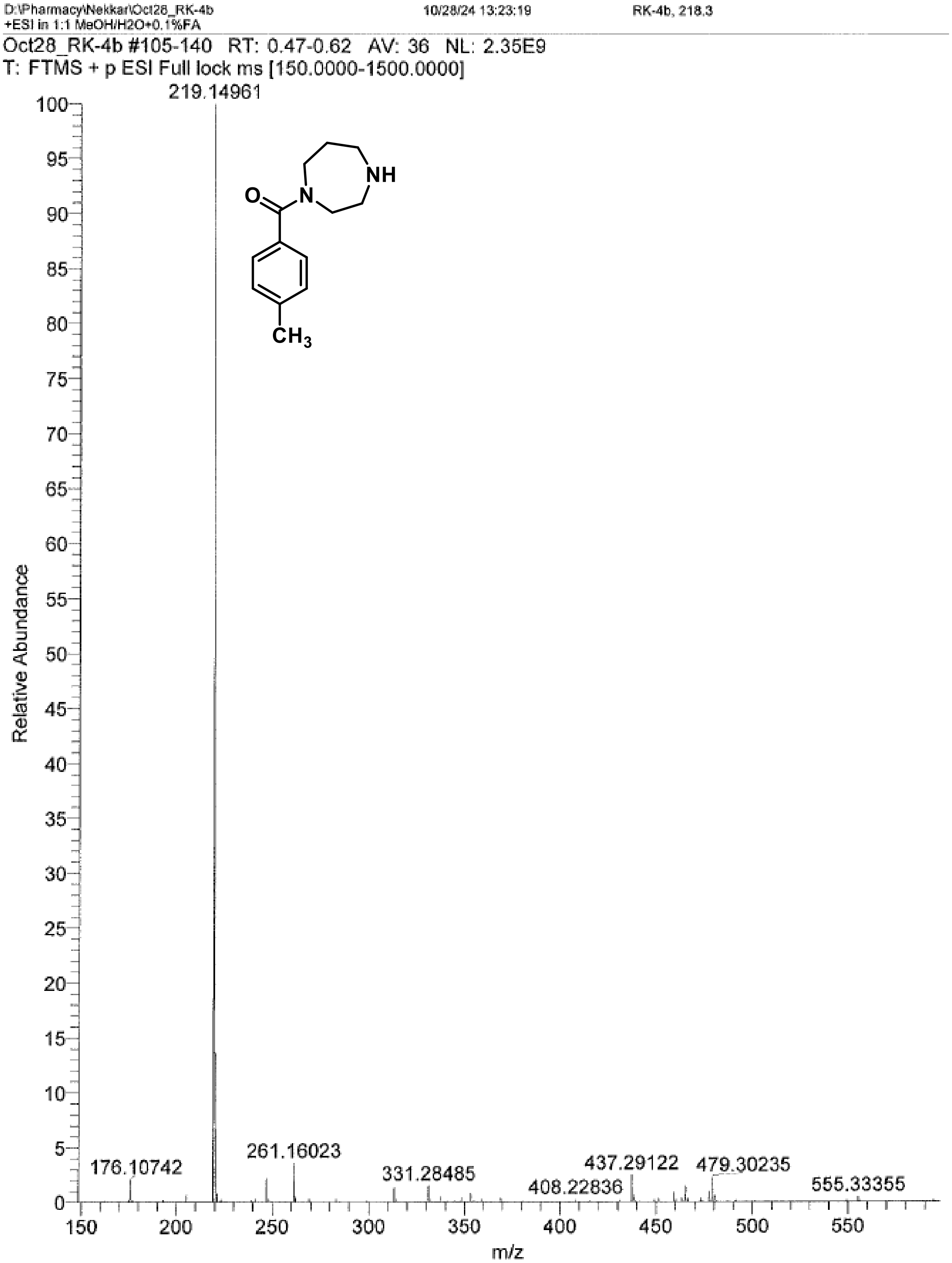
D:\Pharmacy\Nekkar\Oct28_RK-4b
+ESI in 1:1 MeOH/H2O+0.1%FA
10/28/24 13:23:19
RK-4b, 218.3
Oct28_RK-4b #105-140 RT: 0.47-0.62 AV: 36 NL: 2.35E9
T: FTMS + p ESI Full lock ms [150.0000-1500.0000]
219.14961
NH
O
N
CH3
Relative Abundance
176.10742
261.16023
331.28485
408.22836
437.29122
479.30235
555.33355
m/z

(1,4-Diazepan-1-yl)(4-methoxyphenyl)methanone (4c)

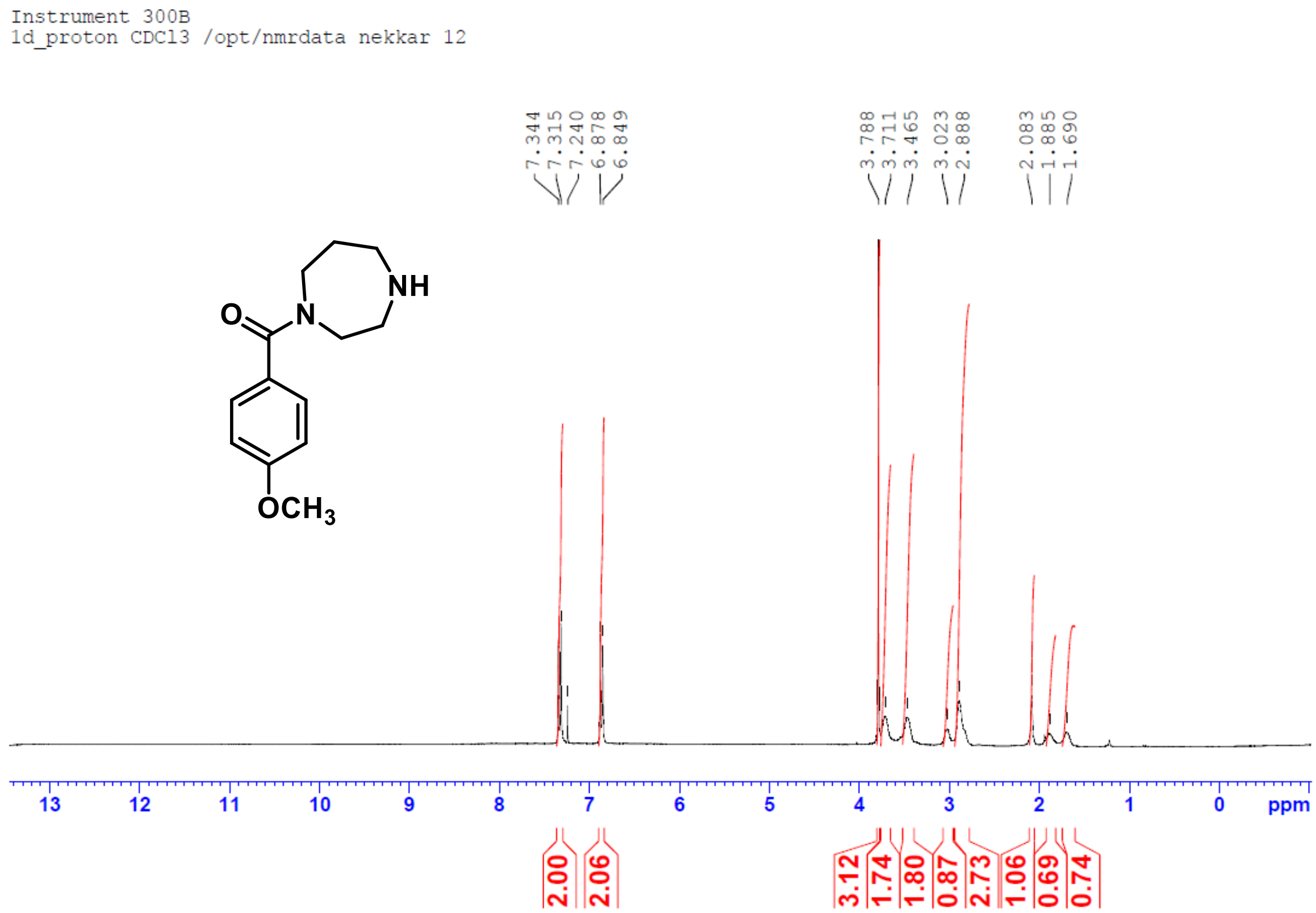

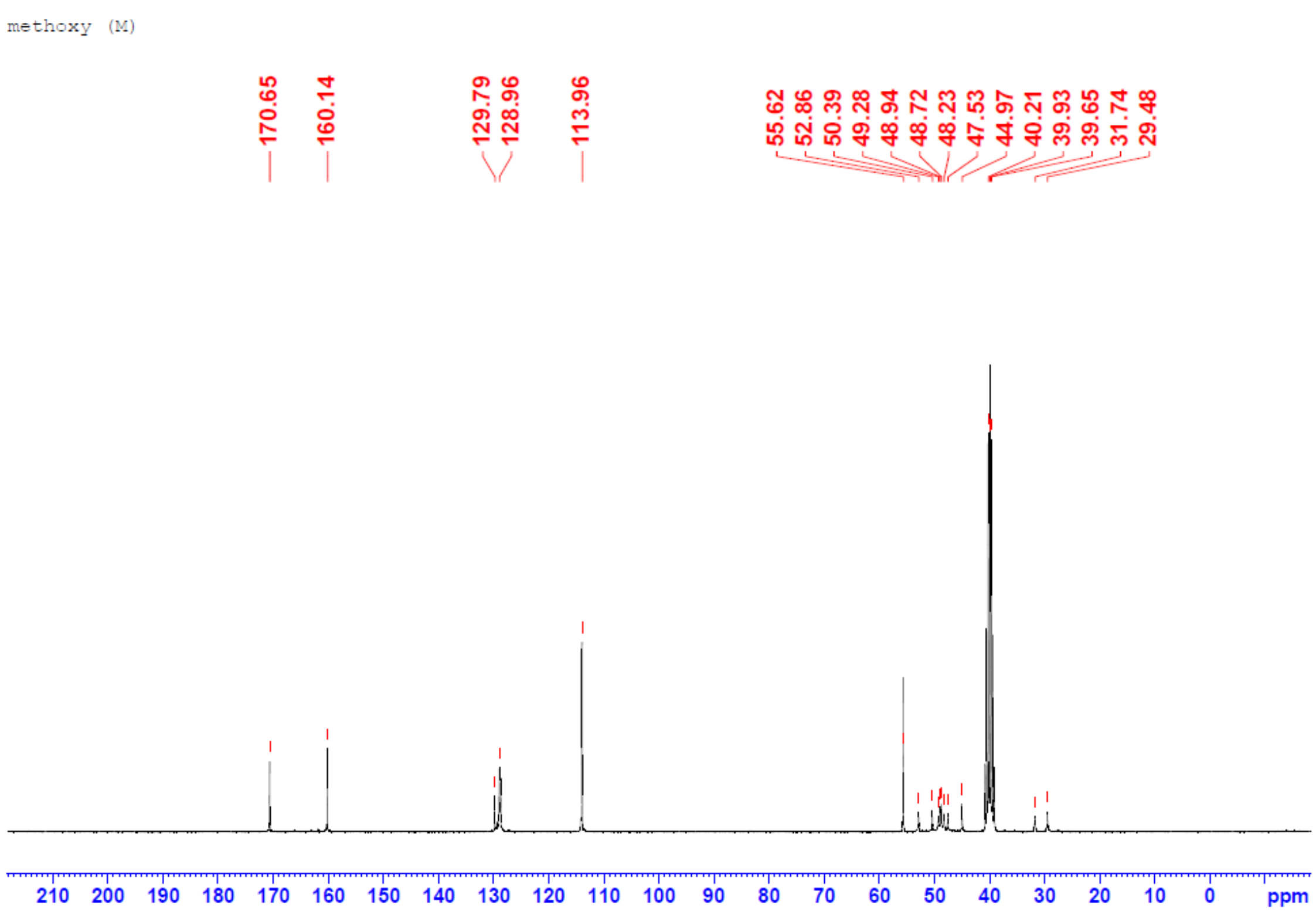

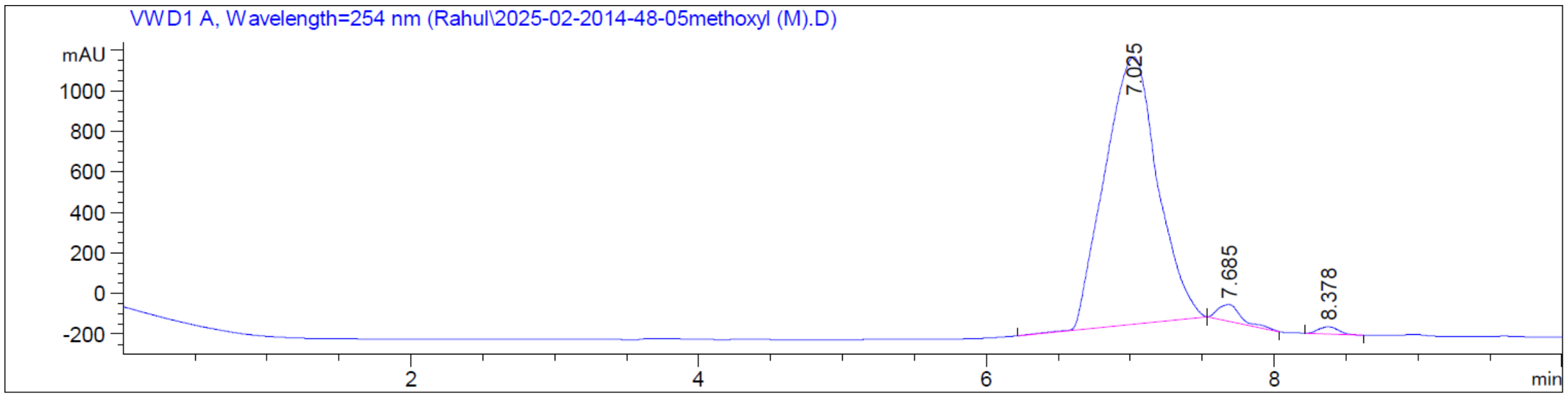

Signal 3: VWD1 A, Wavelength=254 nm

Peak #	RetTime [min]	Type	Width [min]	Area [mAU*s]	Height [mAU]	Area %
1	7.025	BB	0.3904	3.36233e4	1319.12878	96.3711
2	7.685	BB	0.1789	923.08606	82.87625	2.6457
3	8.378	BB	0.1555	343.00247	35.58229	0.9831
Totals :				3.48894e4	1437.58733	

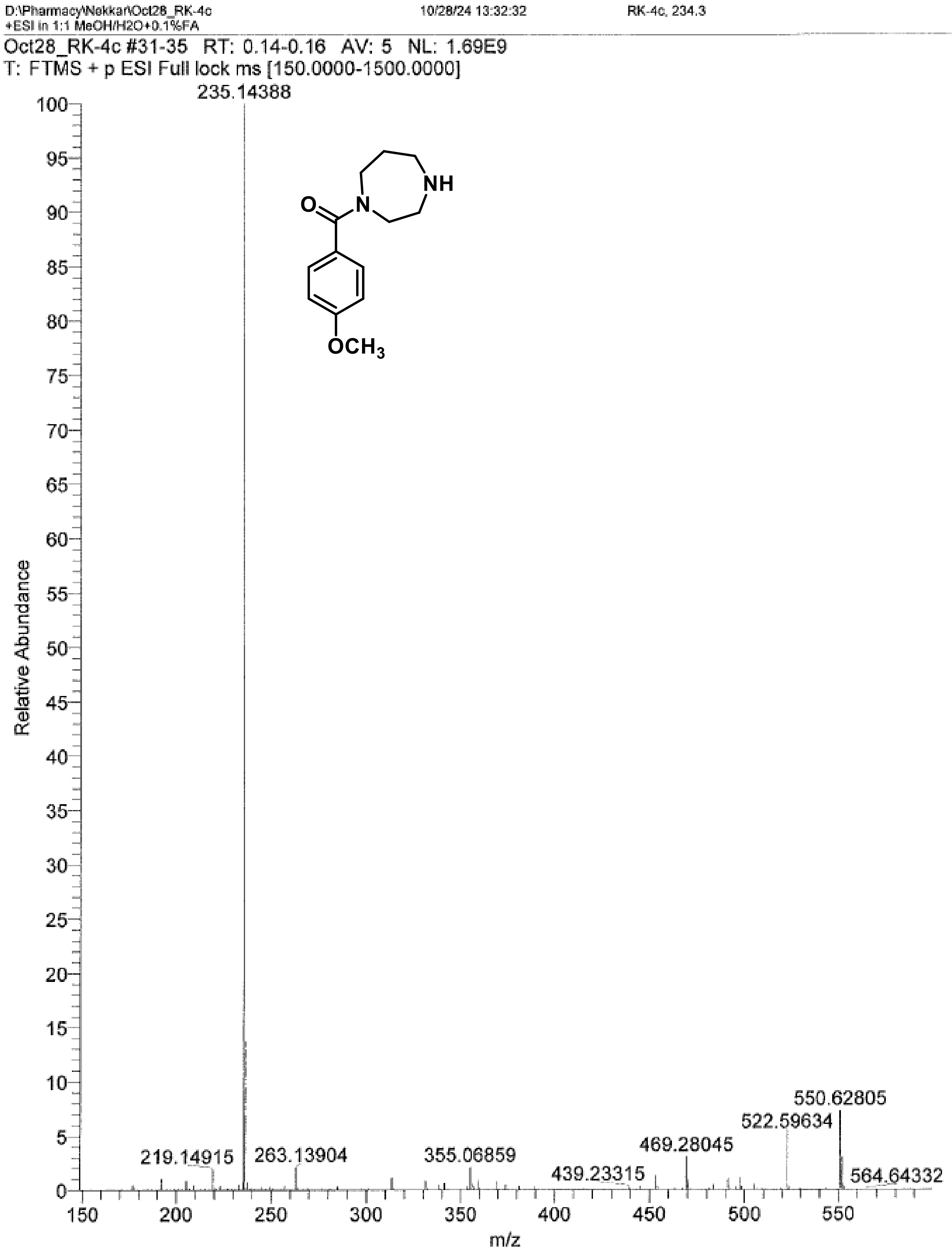
D:\Pharmacy\Nekkar\Oct28_RK-4c
+ESI in 1:1 MeOH/H2O+0.1%FA
10/28/24 13:32:32
RK-4c, 234.3
Oct28_RK-4c #31-35 RT: 0.14-0.16 AV: 5 NL: 1.69E9
T: FTMS + p ESI Full lock ms [150.0000-1500.0000]
235.14388
O
N
NH
OCH3
Relative Abundance
219.14915
263.13904
355.06859
439.23315
469.28045
522.59634
550.62805
564.64332
m/z

(1,4-Diazepan-1-yl)(4-nitrophenyl)methanone (4d)

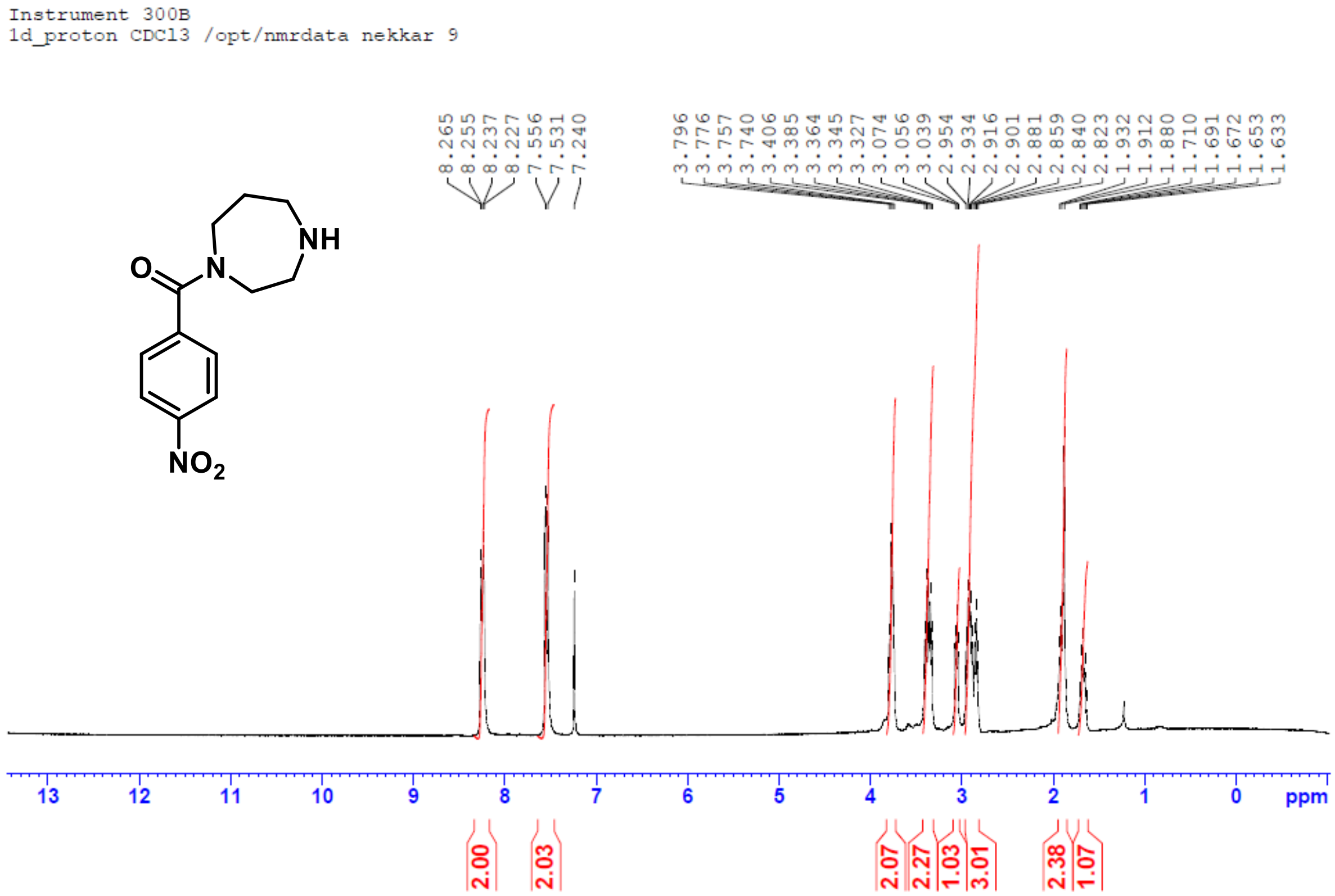

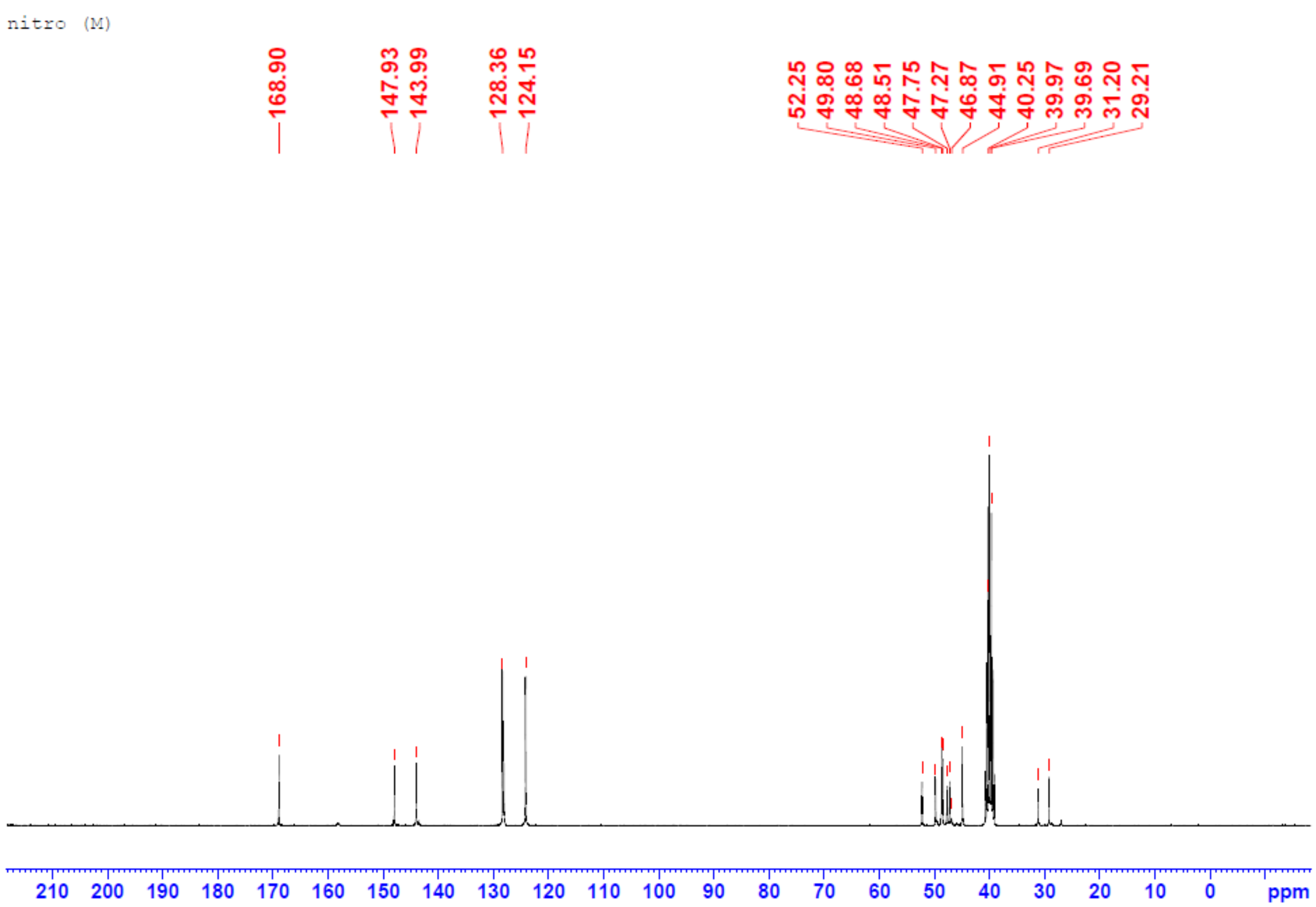

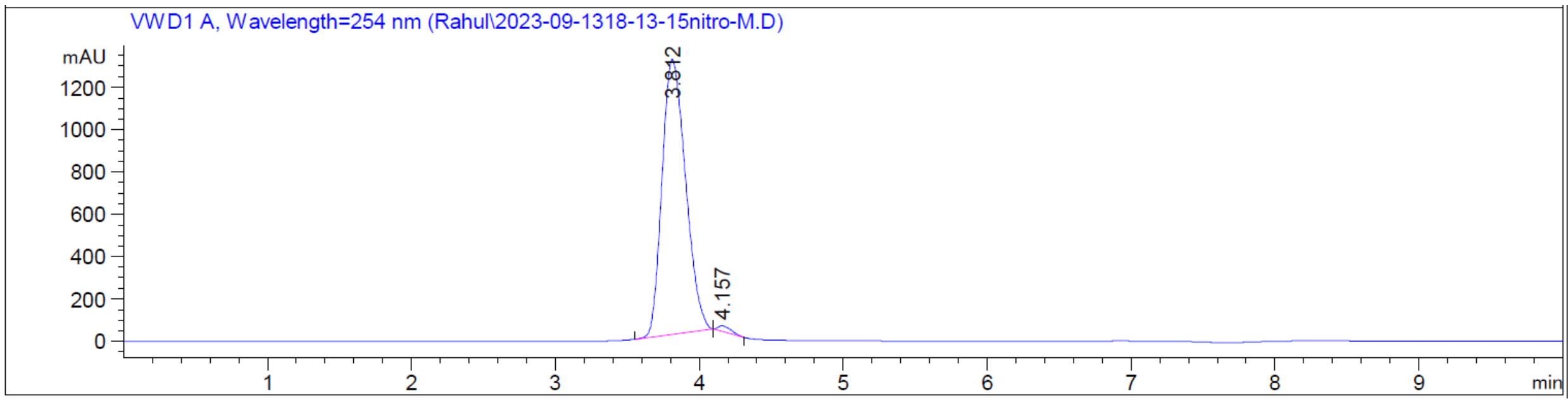

Signal 3: VWD1 A, Wavelength=254 nm

Peak #	RetTime [min]	Type	Width [min]	Area [mAU*s]	Height [mAU]	Area %
1	3.812	BB	0.1794	1.47383e4	1298.98645	98.8531
2	4.157	BB	0.0899	170.99492	26.29789	1.1469

Totals : 1.49093e4 1325.28434

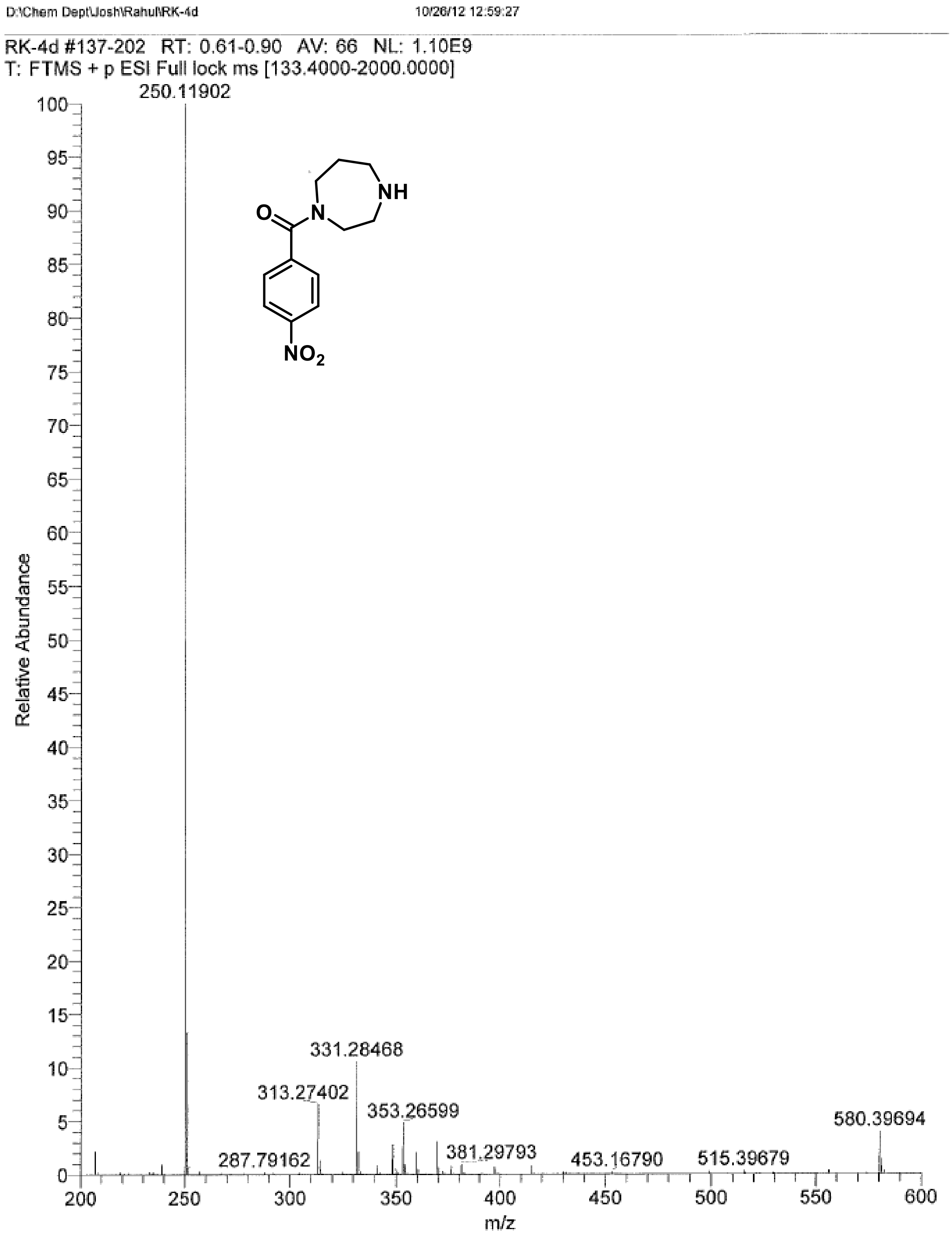
D:\Chem Dept\Josh\Rahul\RK-4d
10/26/12 12:59:27
RK-4d #137-202 RT: 0.61-0.90 AV: 66 NL: 1.10E9
T: FTMS + p ESI Full lock ms [133.4000-2000.0000]
250.11902
331.28468
313.27402
353.26599
580.39694
381.29793
287.79162
453.16790
515.39679
Relative Abundance
m/z
O
N
NH
NO2

(1,4-Diazepan-1-yl)(4-fluorophenyl)methanone (4e)

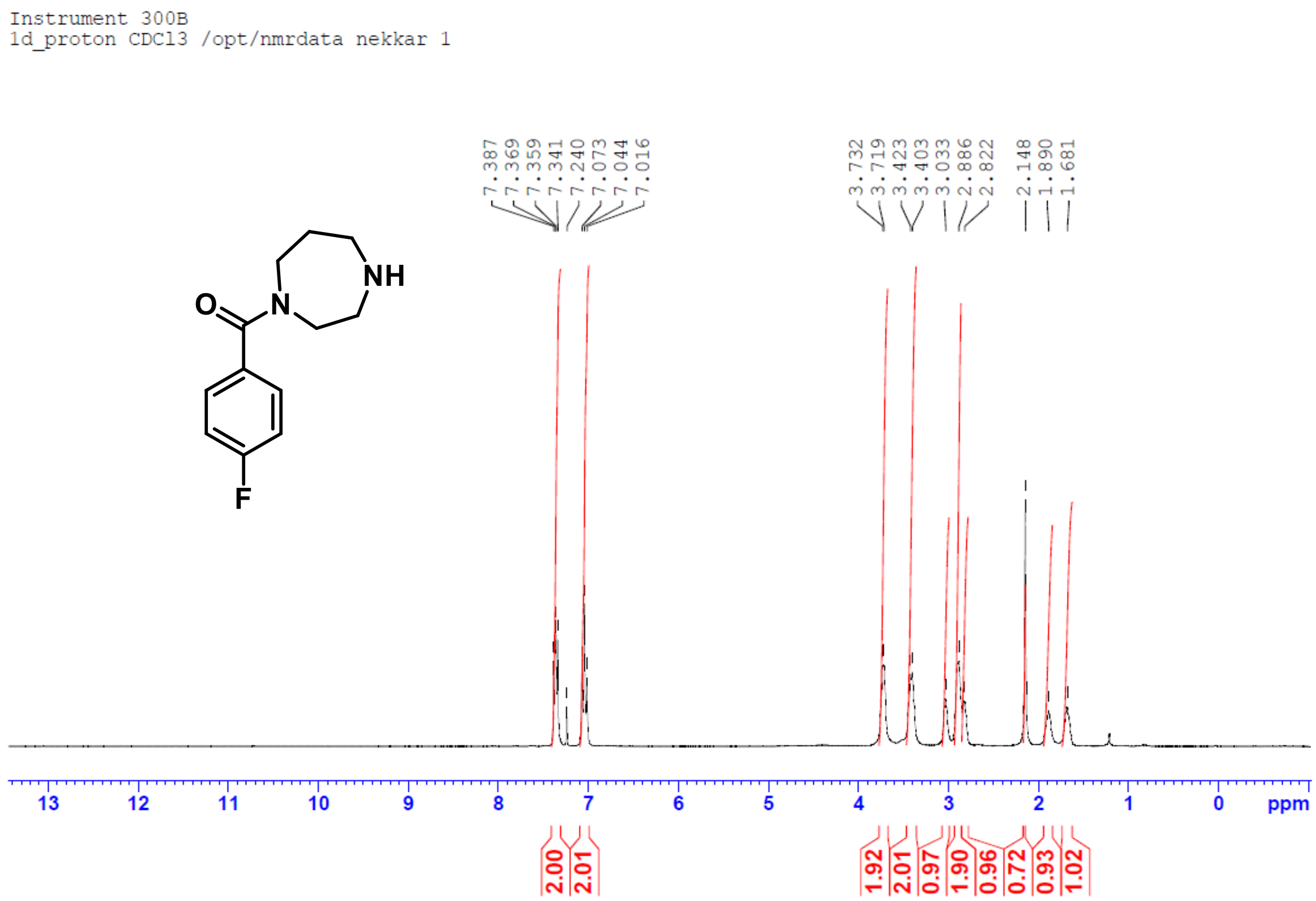

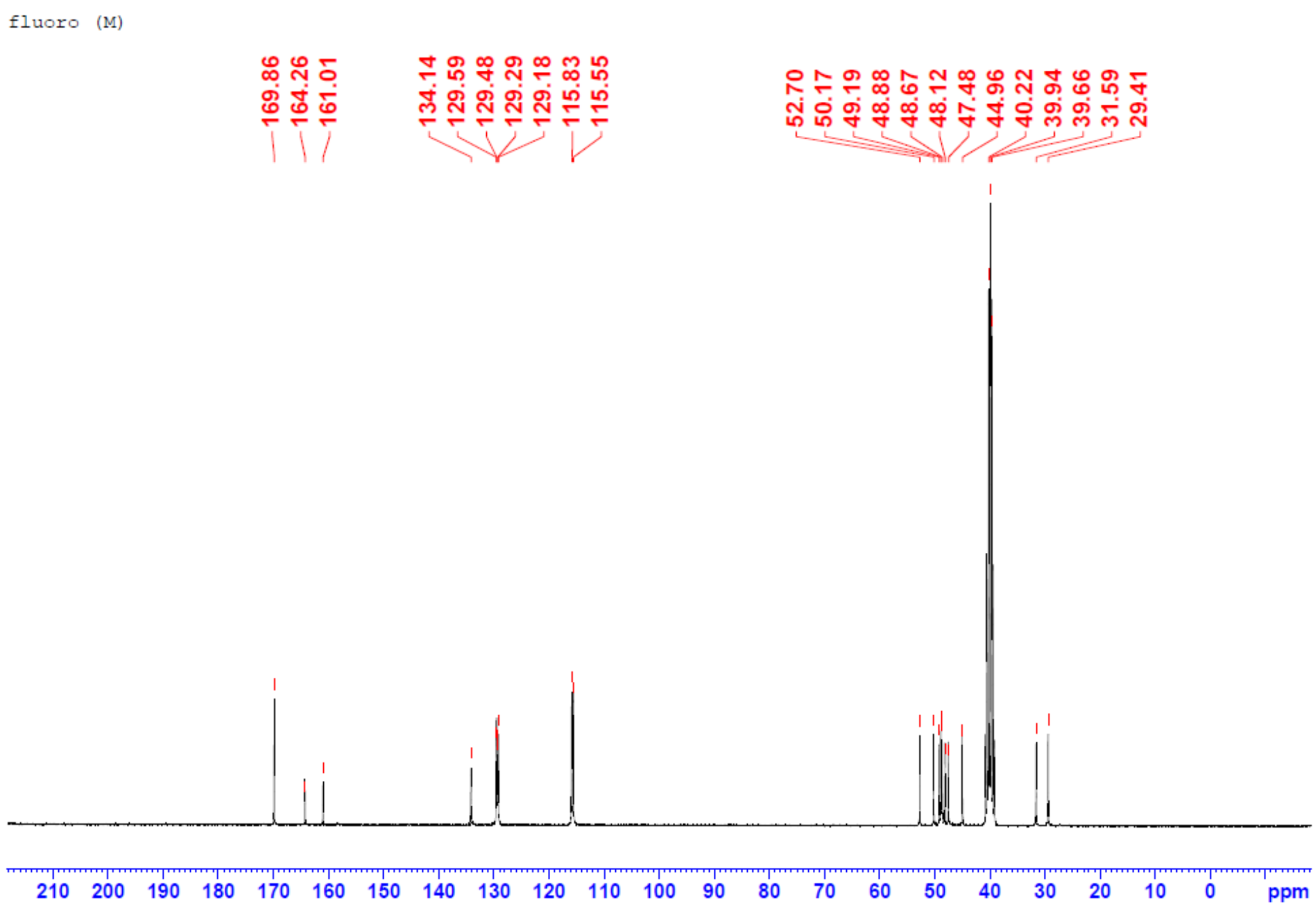

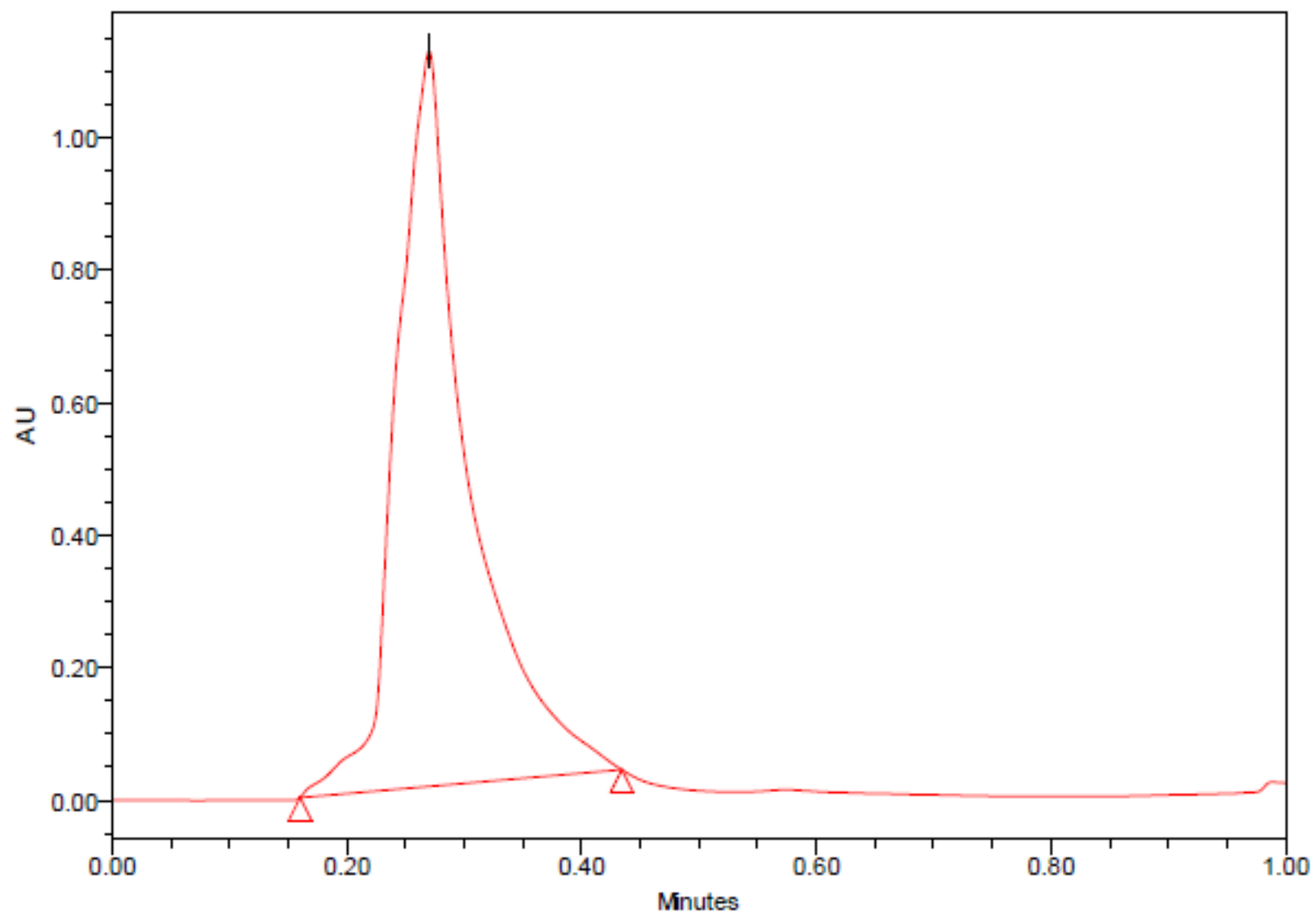

Peak Name:

	Injection	RT	Area	% Area
1	1	0.270	4725998	100.00
Mean		0.270		
Std. Dev.				
% RSD				

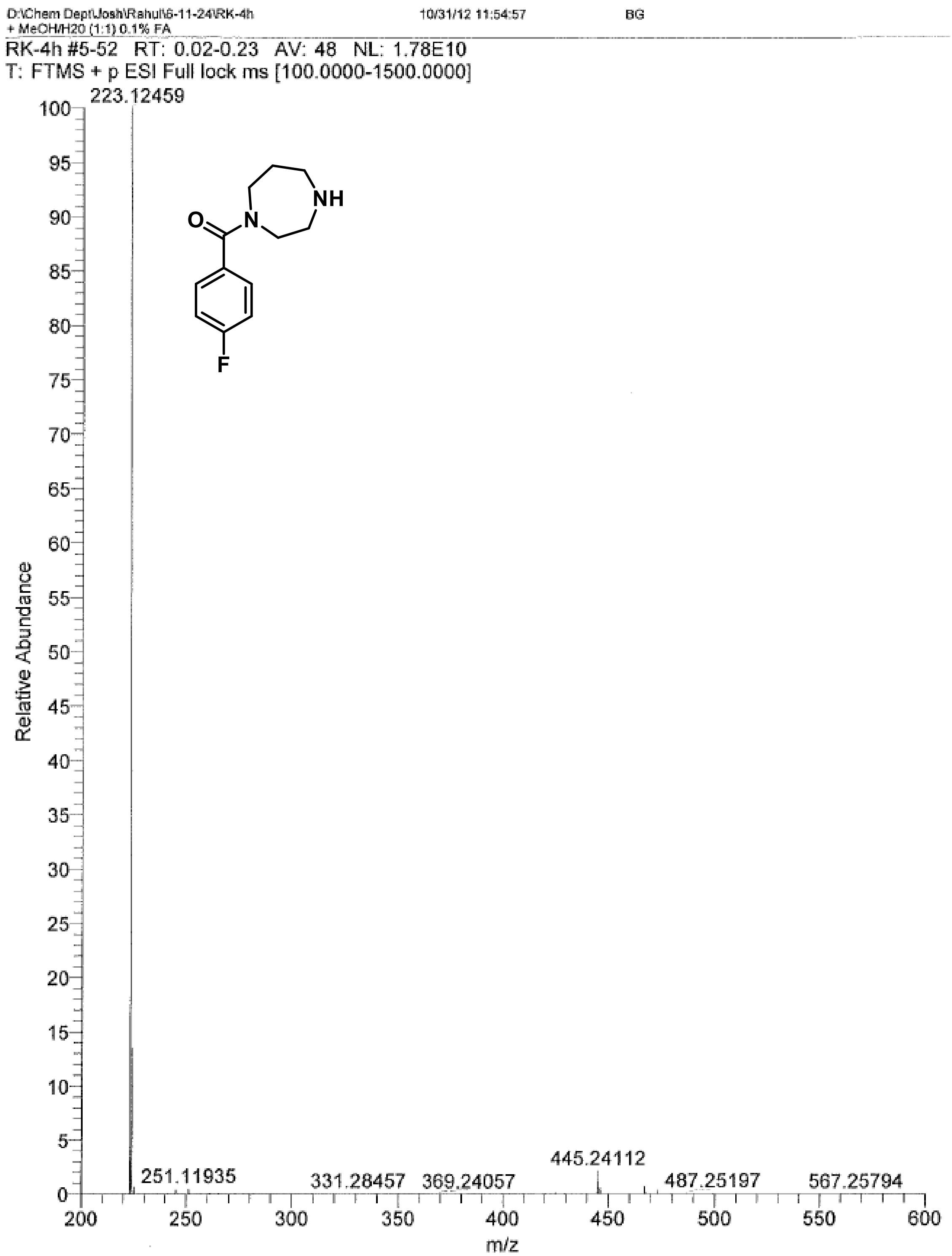
D:\Chem Dept\Josh\Rahul\6-11-24\RK-4h
+ MeOH/H20 (1:1) 0.1% FA
10/31/12 11:54:57
BG
RK-4h #5-52 RT: 0.02-0.23 AV: 48 NL: 1.78E10
T: FTMS + p ESI Full lock ms [100.0000-1500.0000]
223.12459
251.11935
331.28457
369.24057
445.24112
487.25197
567.25794
Relative Abundance
m/z
O
N
NH
F

(4-Chlorophenyl)(1,4-diazepan-1-yl)methanone (4f)

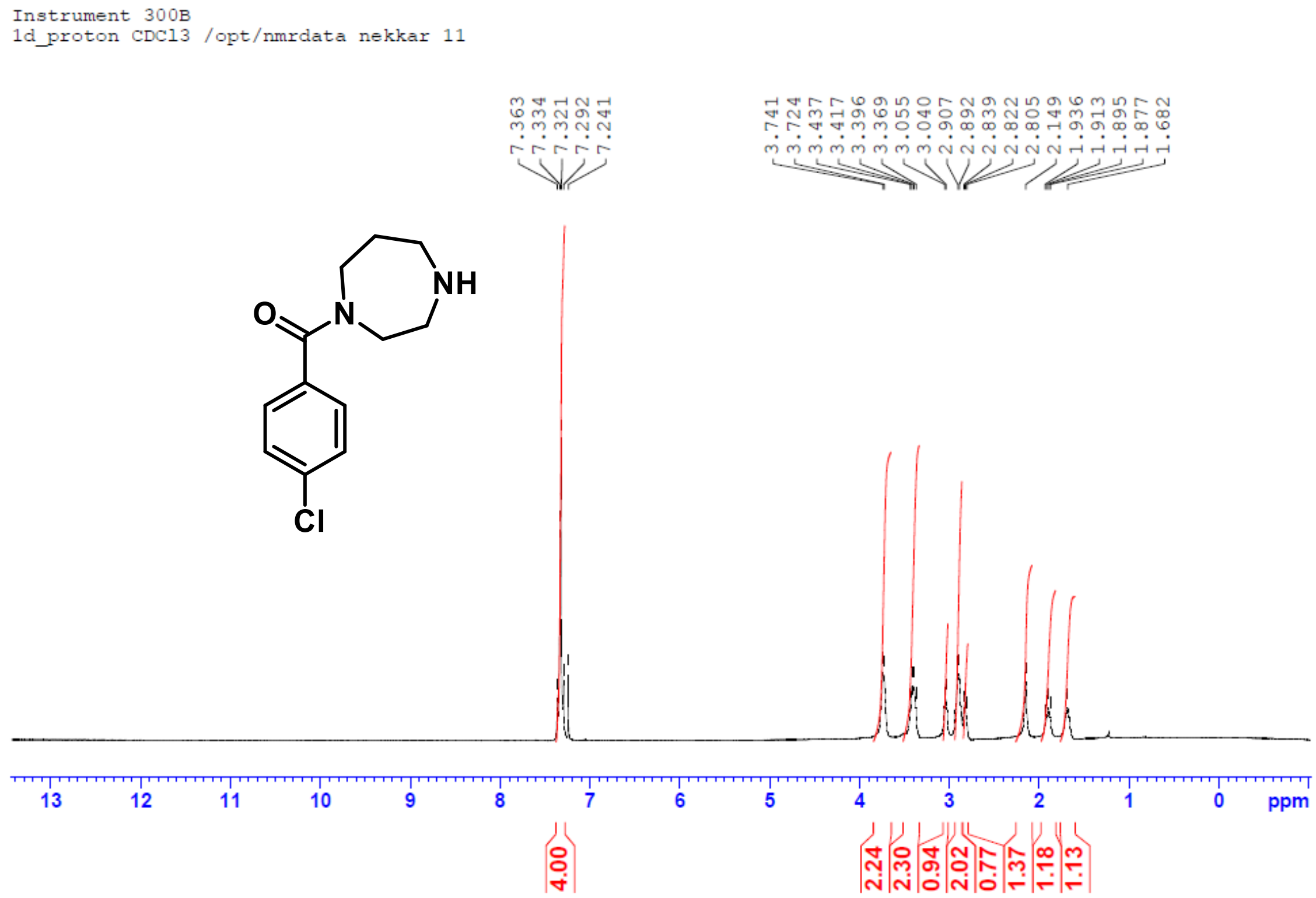

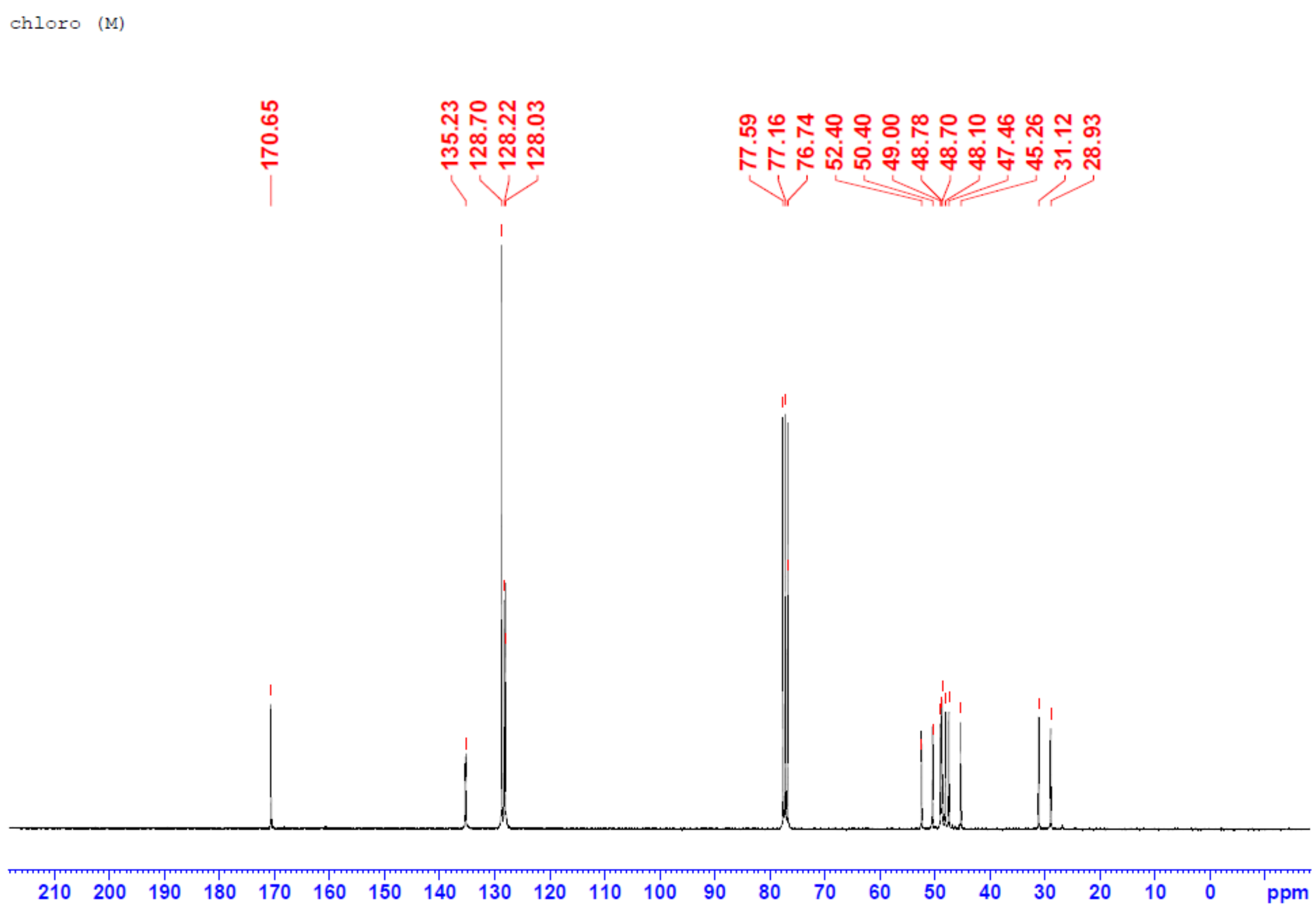

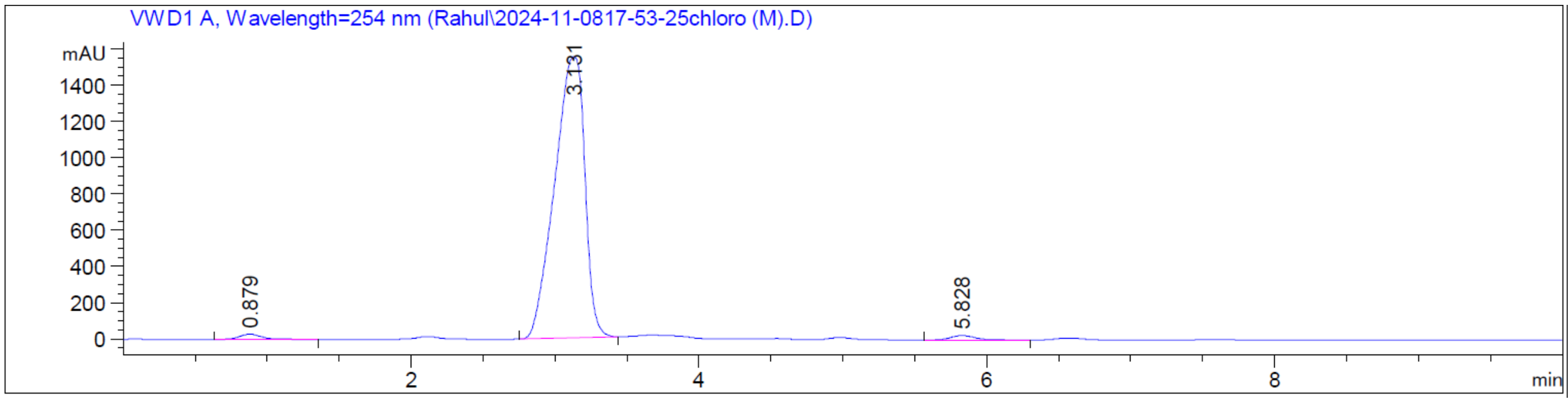

Signal 3: VWD1 A, Wavelength=254 nm

Peak #	RetTime [min]	Type	Width [min]	Area [mAU*s]	Height [mAU]	Area %
1	0.879	BB	0.1551	282.15485	28.10730	1.2172
2	3.131	BB	0.2221	2.26103e4	1551.18701	97.5397
3	5.828	BB	0.1863	288.14740	23.95988	1.2431

Totals : 2.31806e4 1603.25419

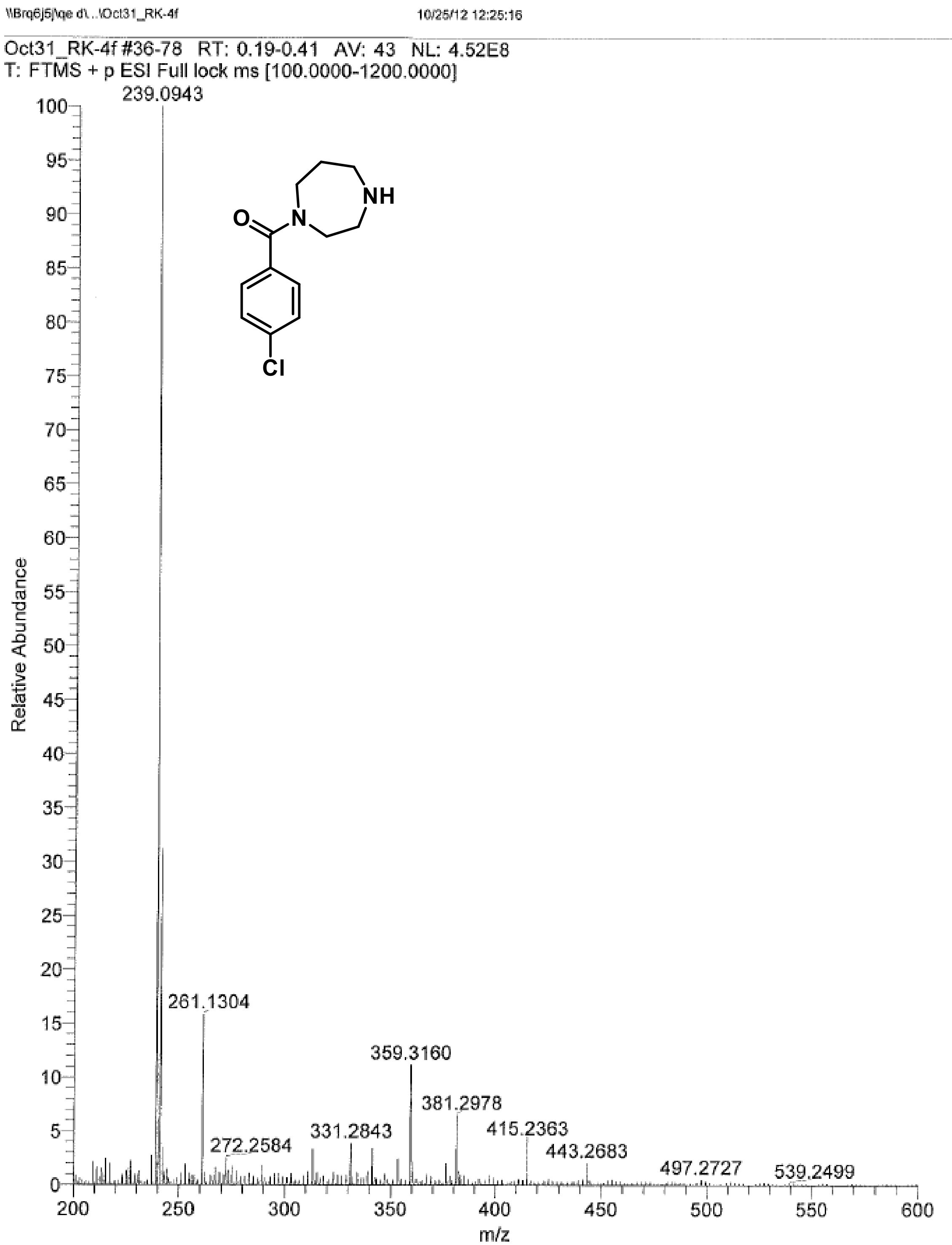
\\Brq6j5j\qe d\...\Oct31_RK-4f
10/25/12 12:25:16
Oct31_RK-4f #36-78 RT: 0.19-0.41 AV: 43 NL: 4.52E8
T: FTMS + p ESI Full lock ms [100.0000-1200.0000]
239.0943
261.1304
359.3160
381.2978
415.2363
331.2843
443.2683
272.2584
497.2727
539.2499
Relative Abundance
m/z
O
N
NH
Cl

(4-Bromophenyl)(1,4-diazepan-1-yl)methanone (4g)

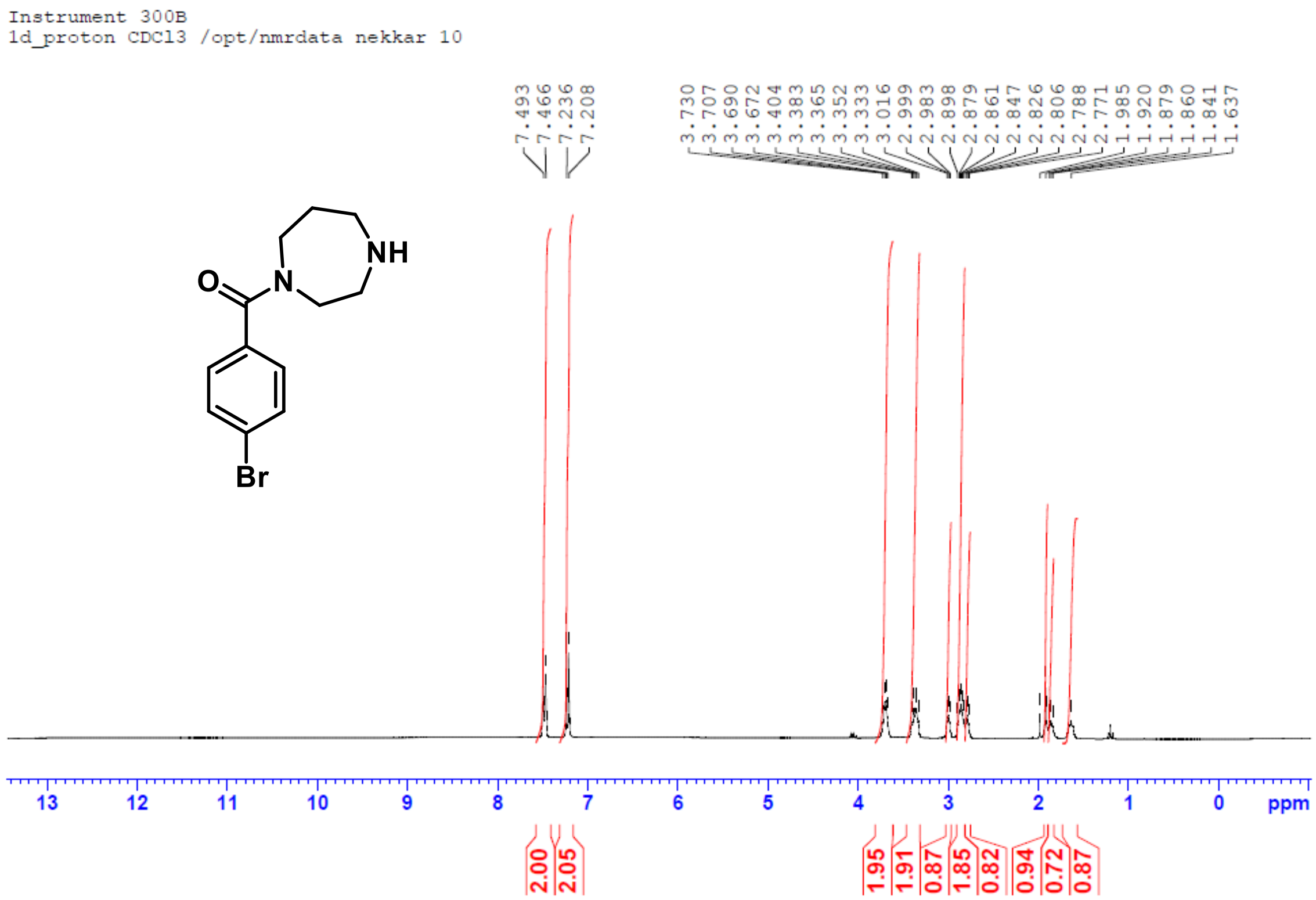

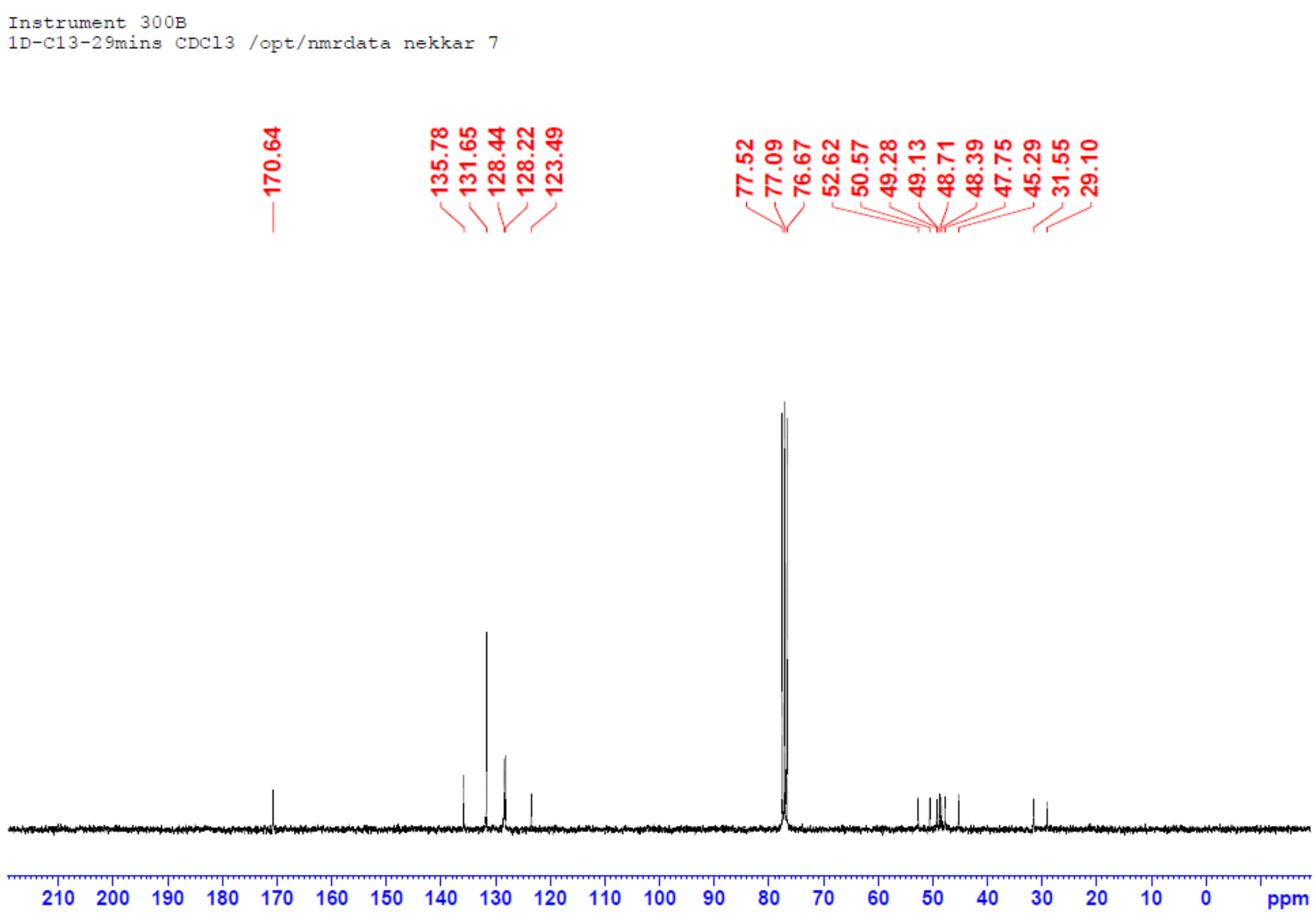

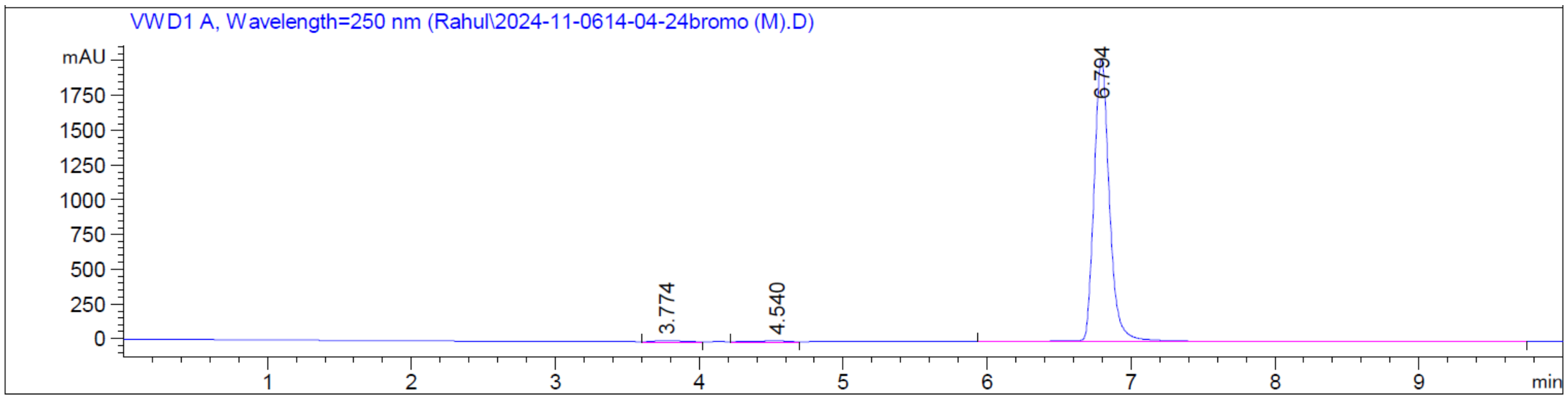

Signal 3: VWD1 A, Wavelength=250 nm

Peak #	RetTime [min]	Type	Width [min]	Area [mAU*s]	Height [mAU]	Area %
1	3.774	BB	0.2221	126.83734	9.07524	0.8214
2	4.540	BB	0.3216	121.13029	6.52443	0.7844
3	6.794	BBA	0.1133	1.51935e4	2030.60034	98.3941

Totals : 1.54414e4 2046.20002

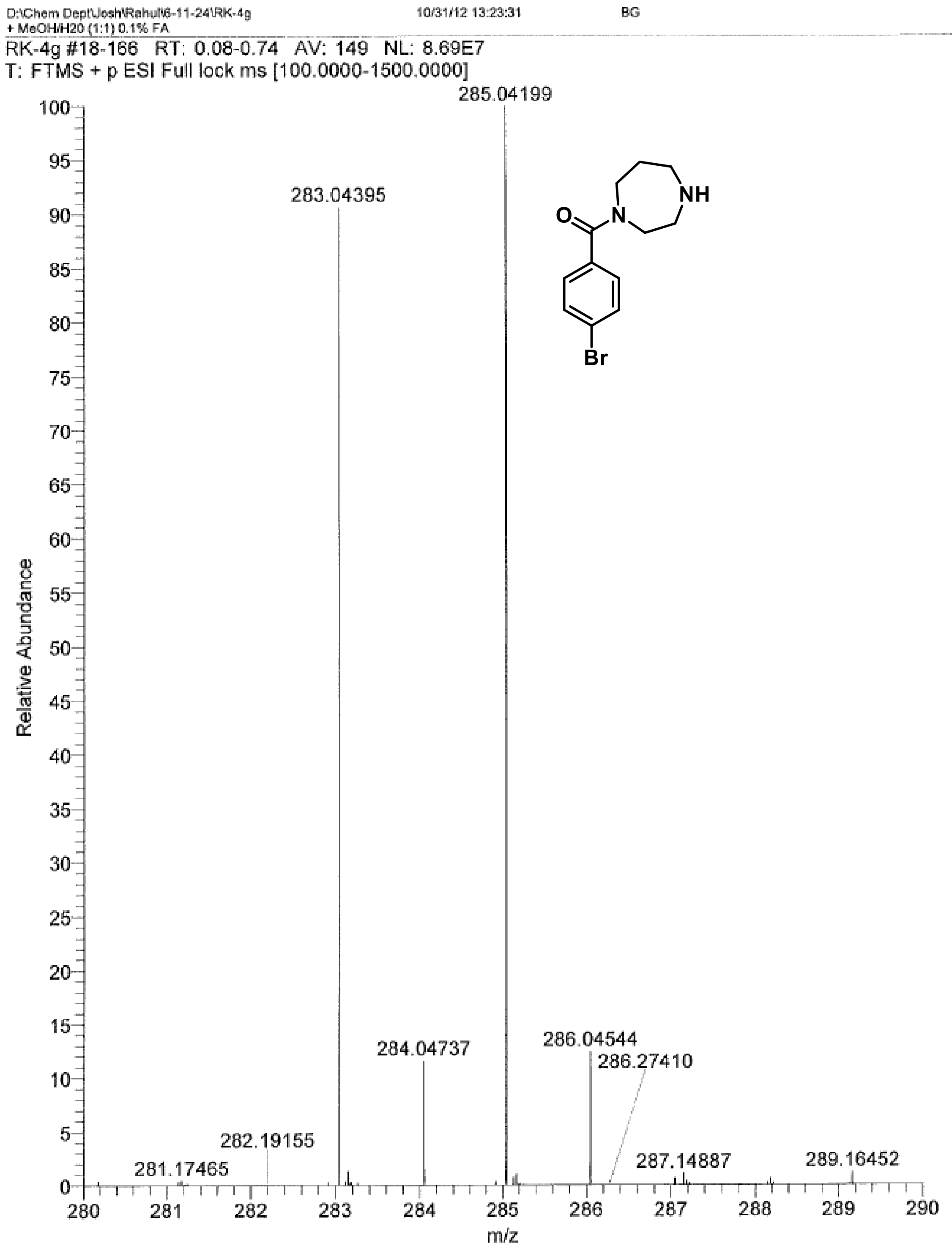
D:\Chem Dept\Josh\Rahul\6-11-24\RK-4g
+ MeOH/H20 (1:1) 0.1% FA
10/31/12 13:23:31
BG
RK-4g #18-166 RT: 0.08-0.74 AV: 149 NL: 8.69E7
T: FTMS + p ESI Full lock ms [100.0000-1500.0000]
285.04199
283.04395
284.04737
286.04544
286.27410
282.19155
281.17465
287.14887
289.16452
Relative Abundance
m/z
O
N
NH
Br

(1,4-Diazepan-1-yl)(4-iodophenyl)methanone (4h)

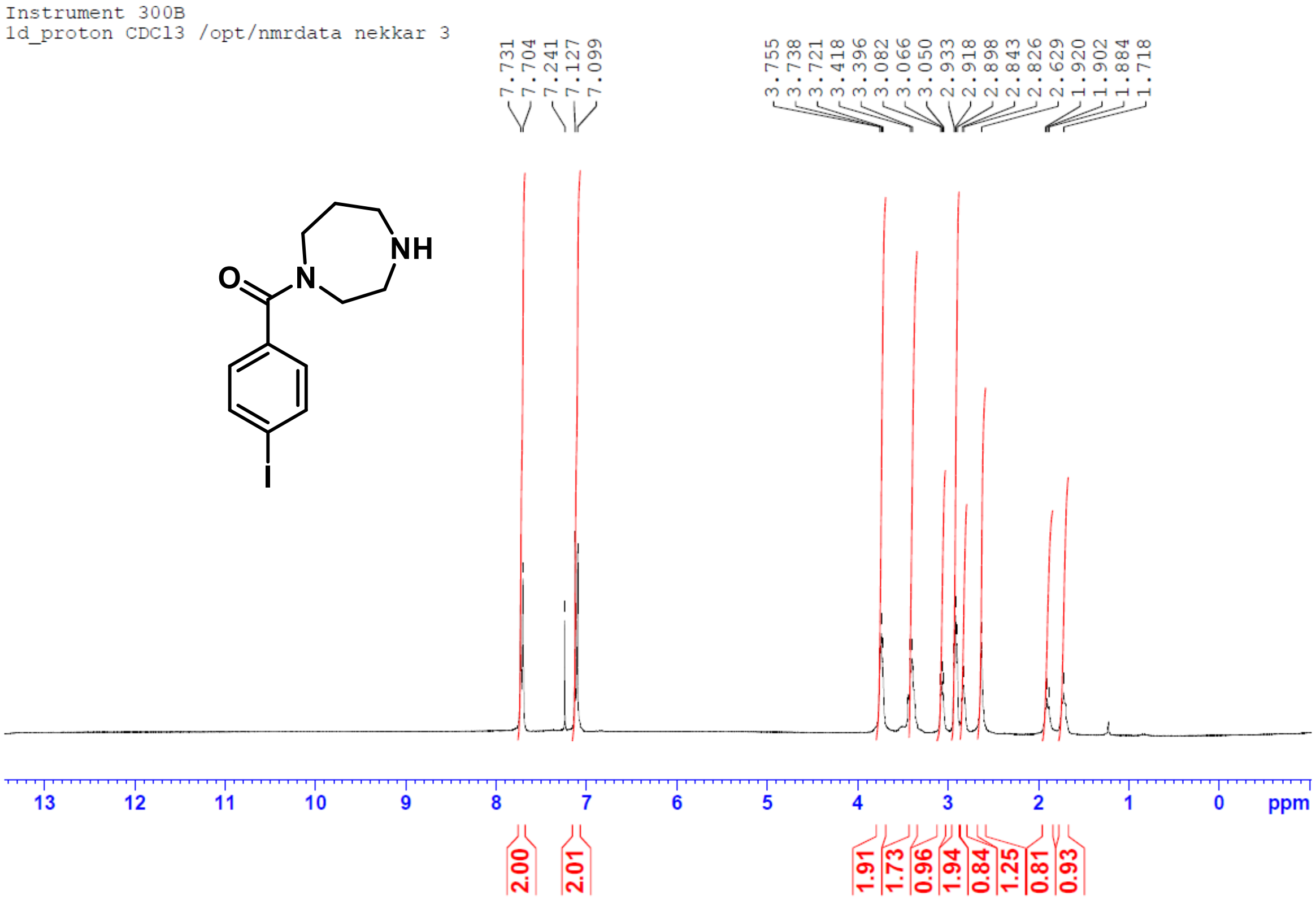

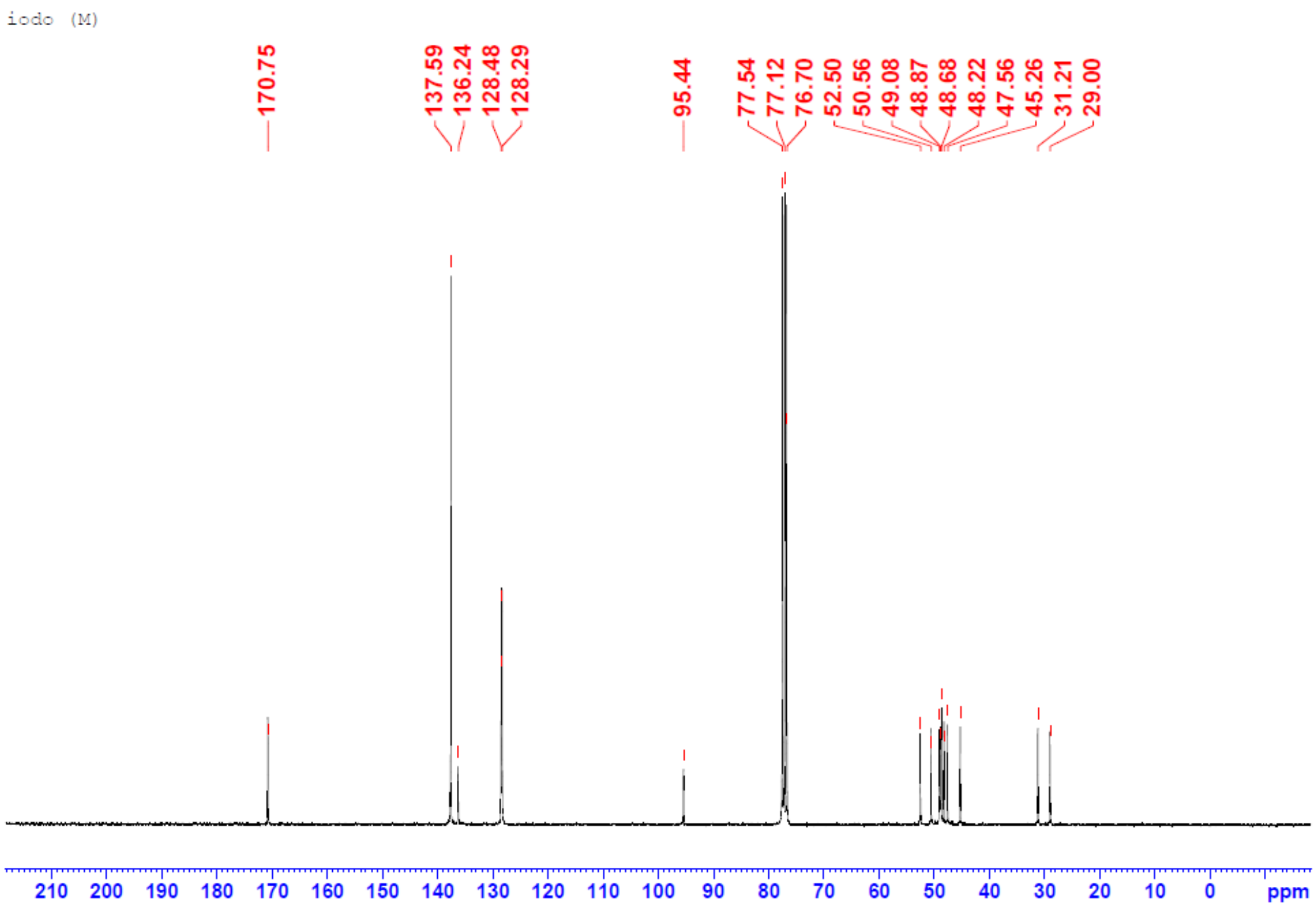

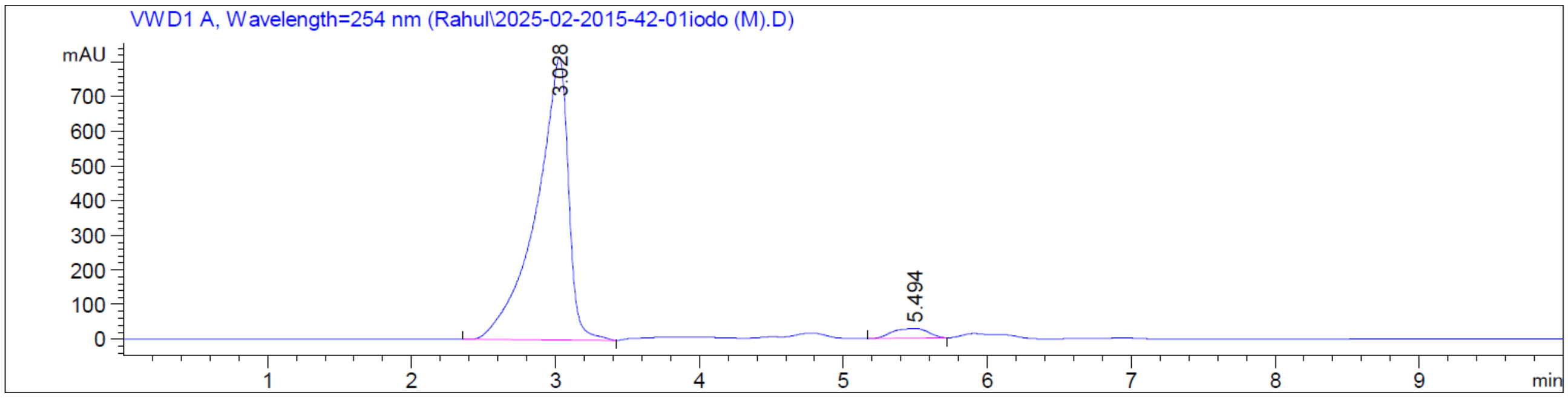

Signal 3: VWD1 A, Wavelength=254 nm

Peak #	RetTime [min]	Type	Width [min]	Area [mAU*s]	Height [mAU]	Area %
1	3.028	BB	0.2158	1.27255e4	816.57562	96.3521
2	5.494	BB	0.2895	481.78824	27.76330	3.6479

Totals : 1.32073e4 844.33893

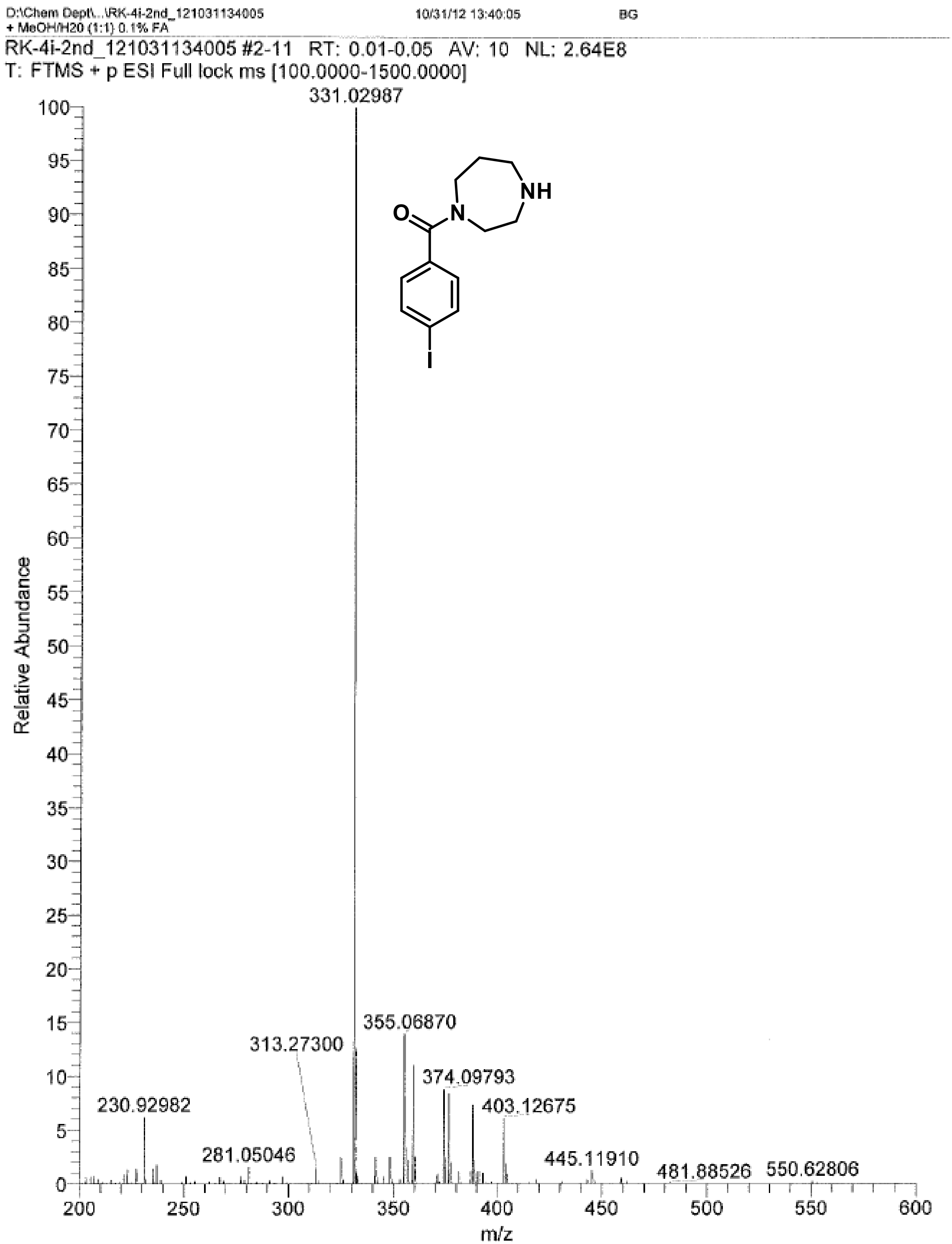
D:\Chem Dept\...\RK-4i-2nd_121031134005
+ MeOH/H20 (1:1) 0.1% FA
10/31/12 13:40:05
BG
RK-4i-2nd_121031134005 #2-11 RT: 0.01-0.05 AV: 10 NL: 2.64E8
T: FTMS + p ESI Full lock ms [100.0000-1500.0000]
331.02987
355.06870
313.27300
374.09793
403.12675
230.92982
281.05046
445.11910
481.88526
550.62806
Relative Abundance
m/z
O
N
NH
I

(1,4-Diazepan-1-yl)(3,4-dimethoxyphenyl)methanone (4i)

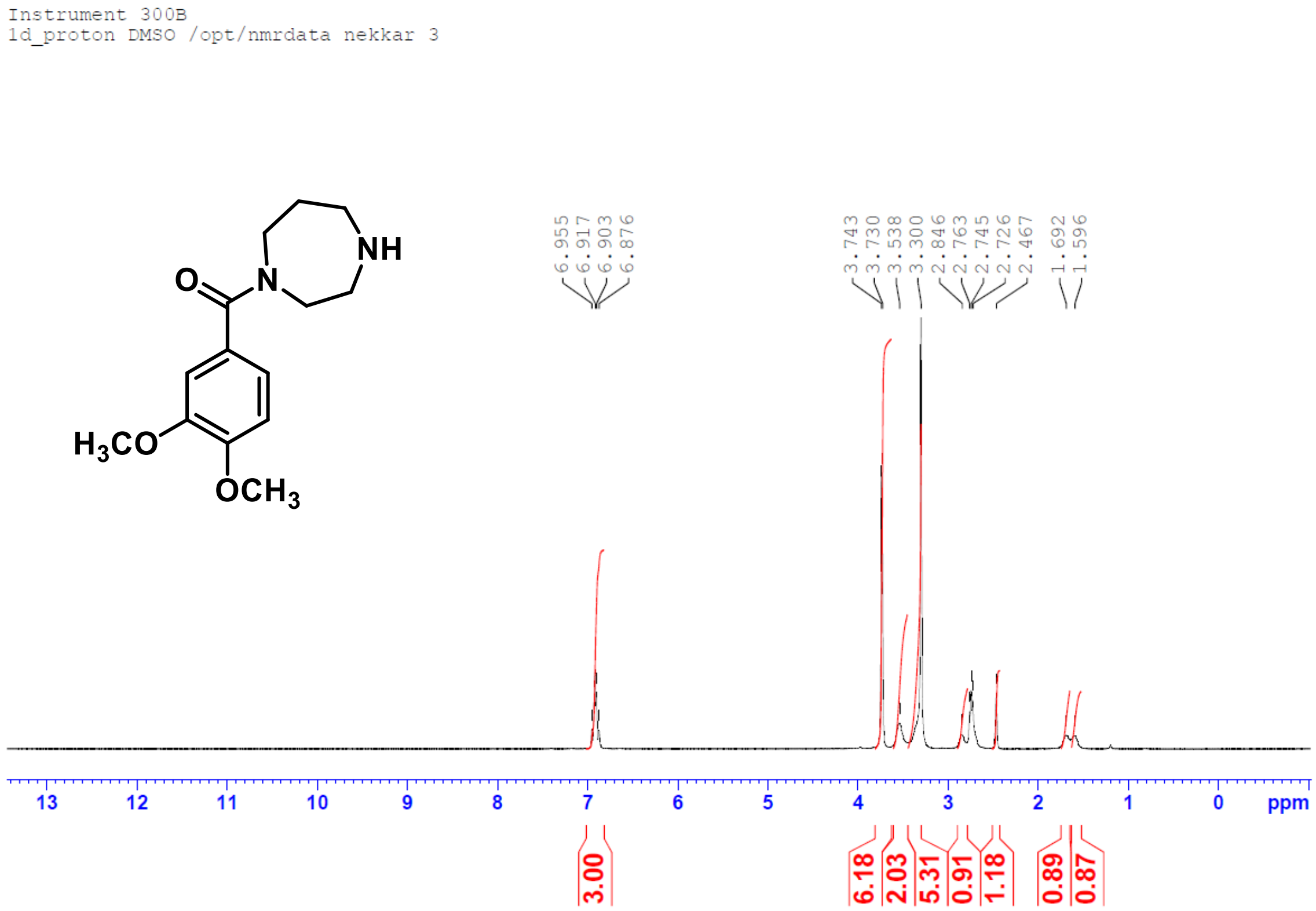

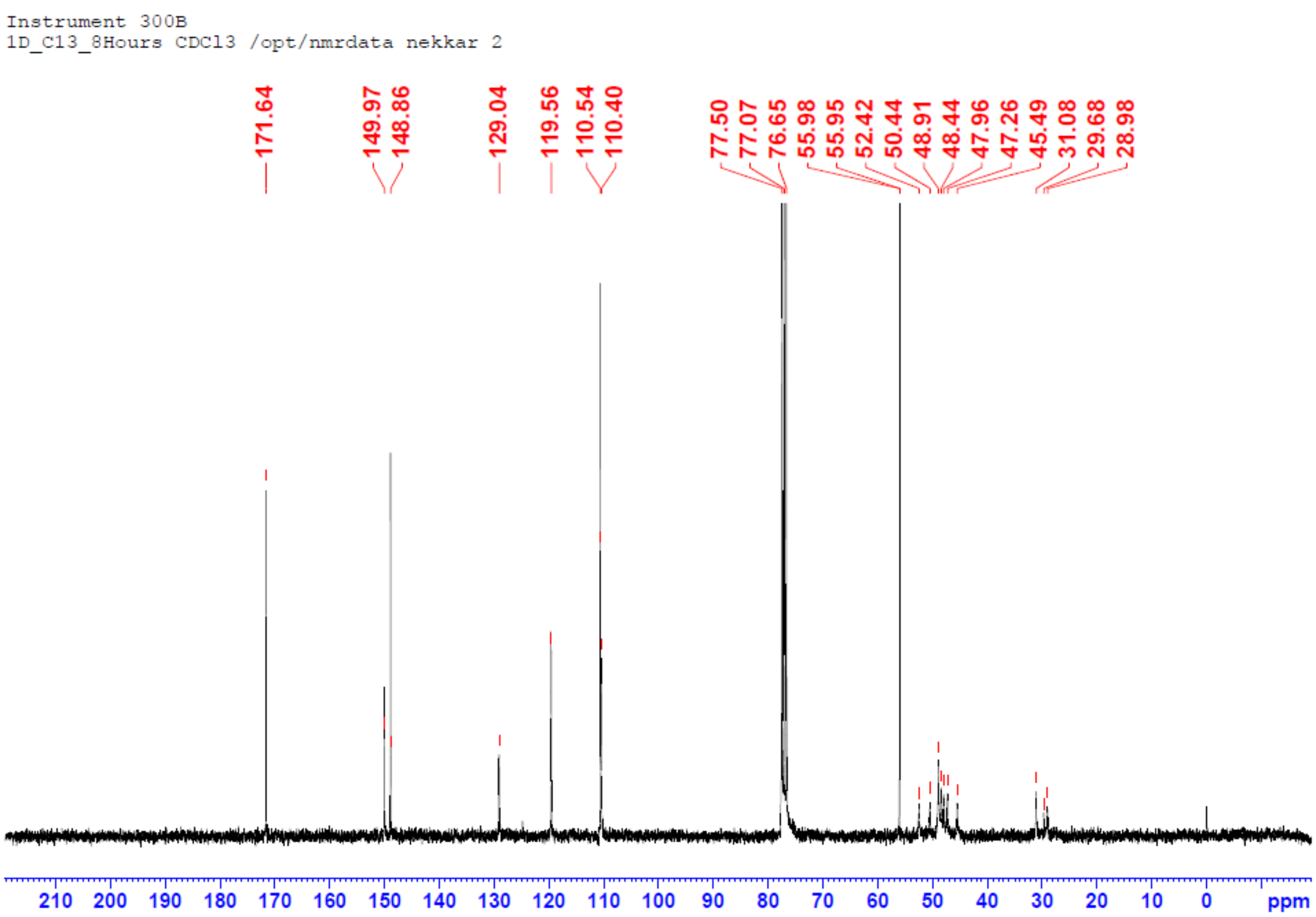

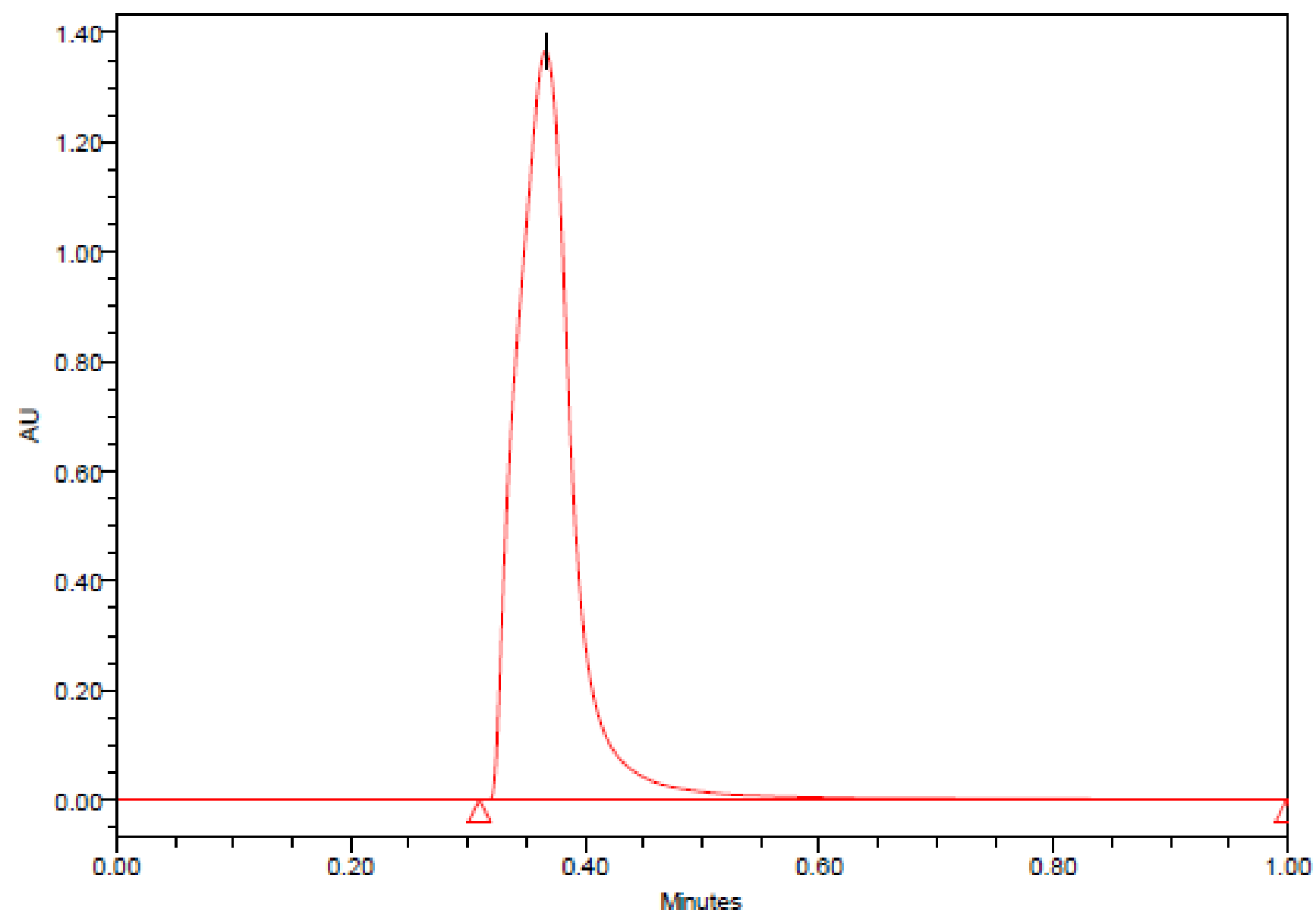

Peak Name:

	Injection	RT	Area	% Area
1	1	0.366	4440337	100.00
Mean		0.366		
Std. Dev.				
% RSD				

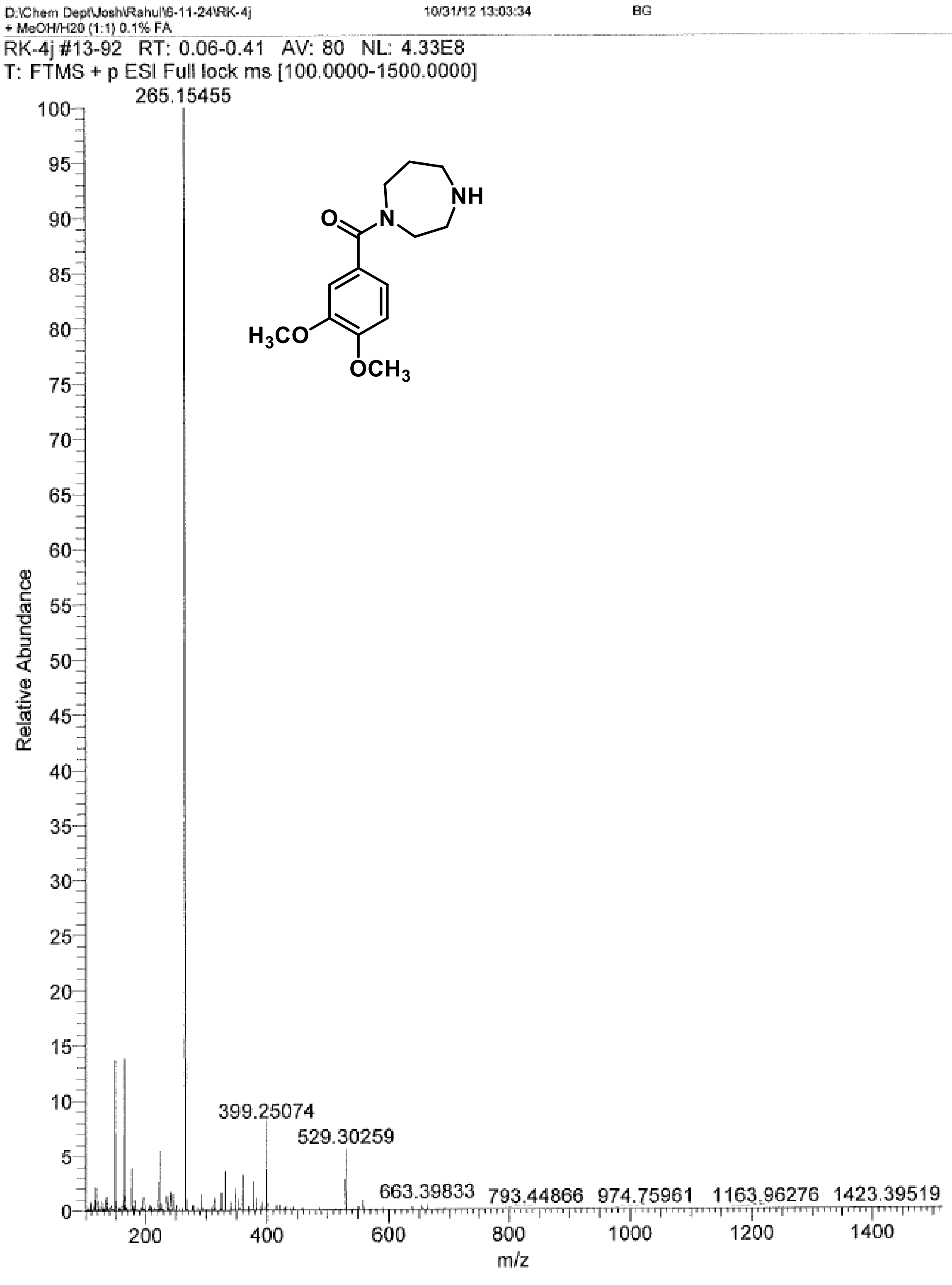
D:\Chem Dept\Josh\Rahul\6-11-24\RK-4j
+ MeOH/H20 (1:1) 0.1% FA
10/31/12 13:03:34
BG
RK-4j #13-92 RT: 0.06-0.41 AV: 80 NL: 4.33E8
T: FTMS + p ESI Full lock ms [100.0000-1500.0000]
265.15455
399.25074
529.30259
663.39833
793.44866
974.75961
1163.96276
1423.39519
Relative Abundance
m/z
NH
O
N
H3CO
OCH3

(1,4-Diazepan-1-yl)(3,4-difluorophenyl)methanone (4j)

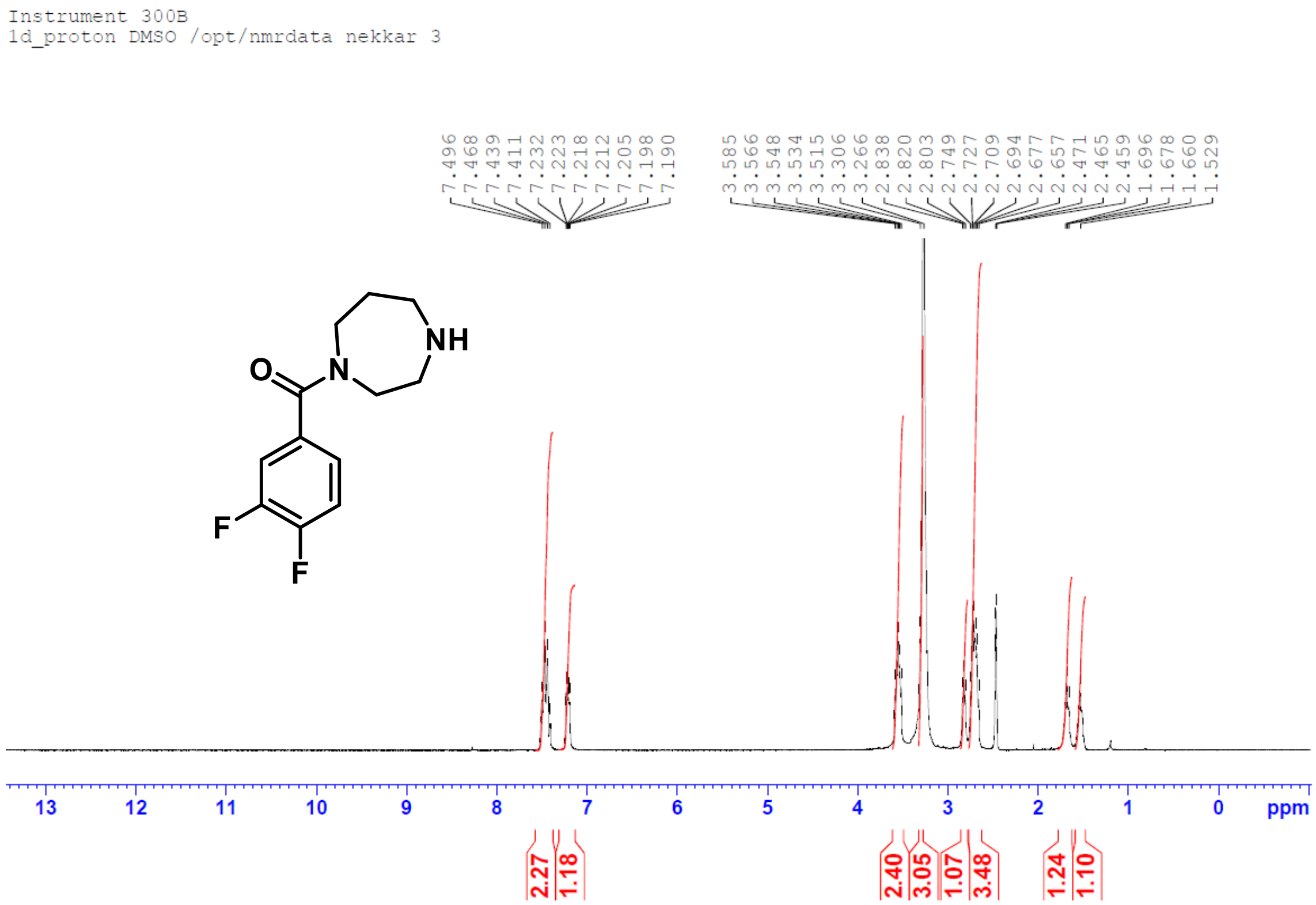

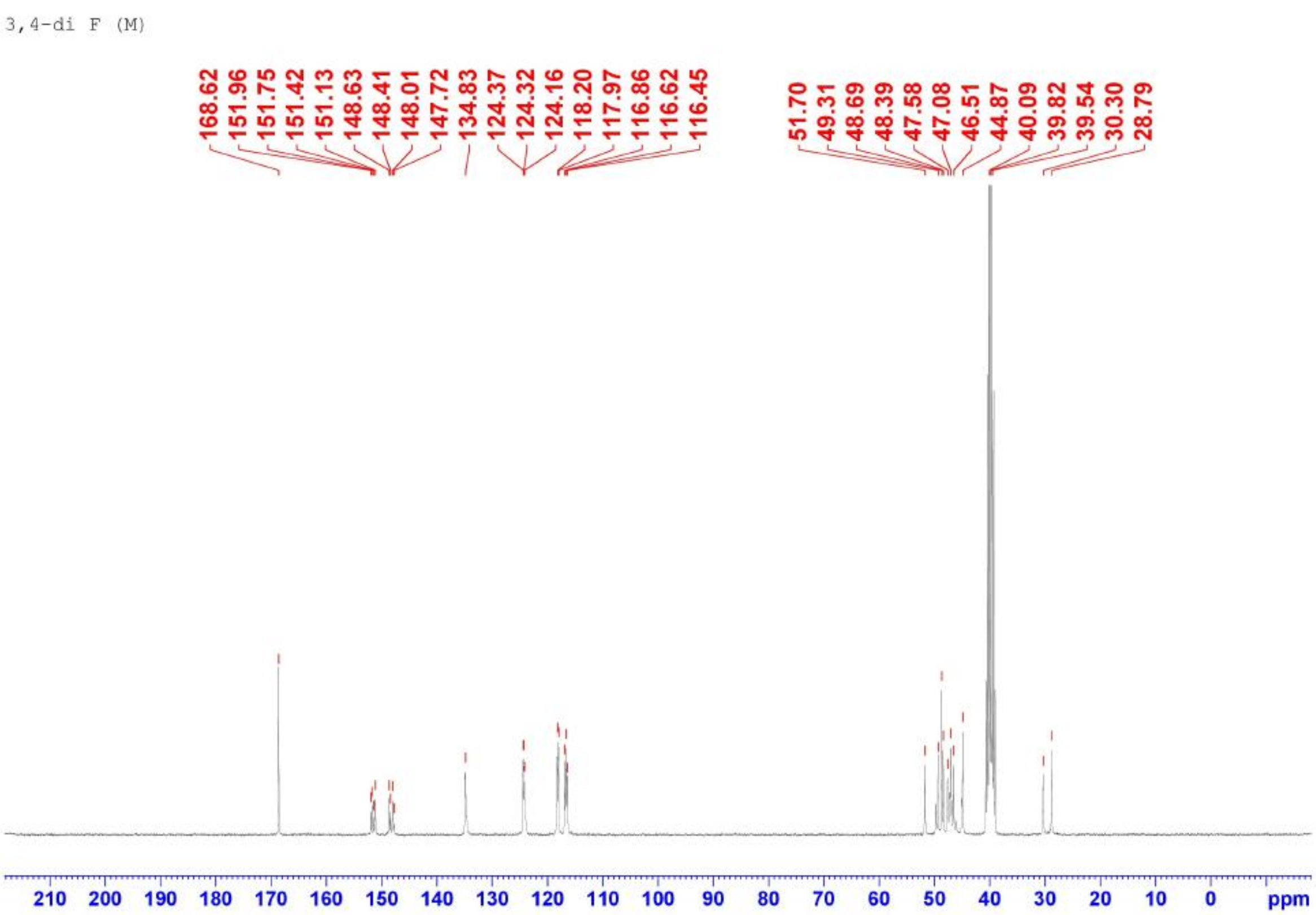

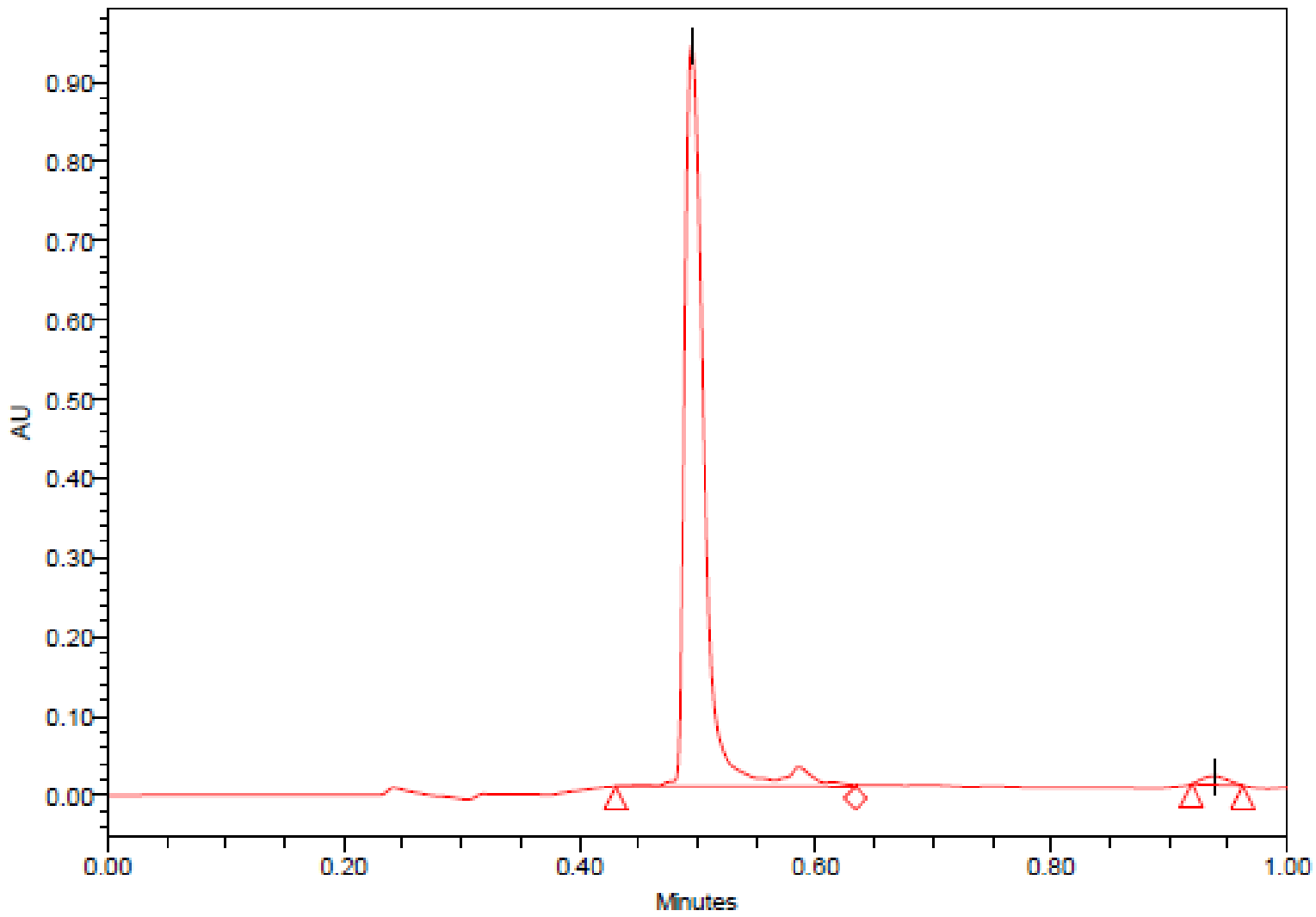

Peak Name:

	Injection	RT	Area	% Area
1	1	0.938	15939	1.46
2	1	0.495	1073739	98.54
Mean		0.716		
Std. Dev.		0.313		
% RSD		43.66		

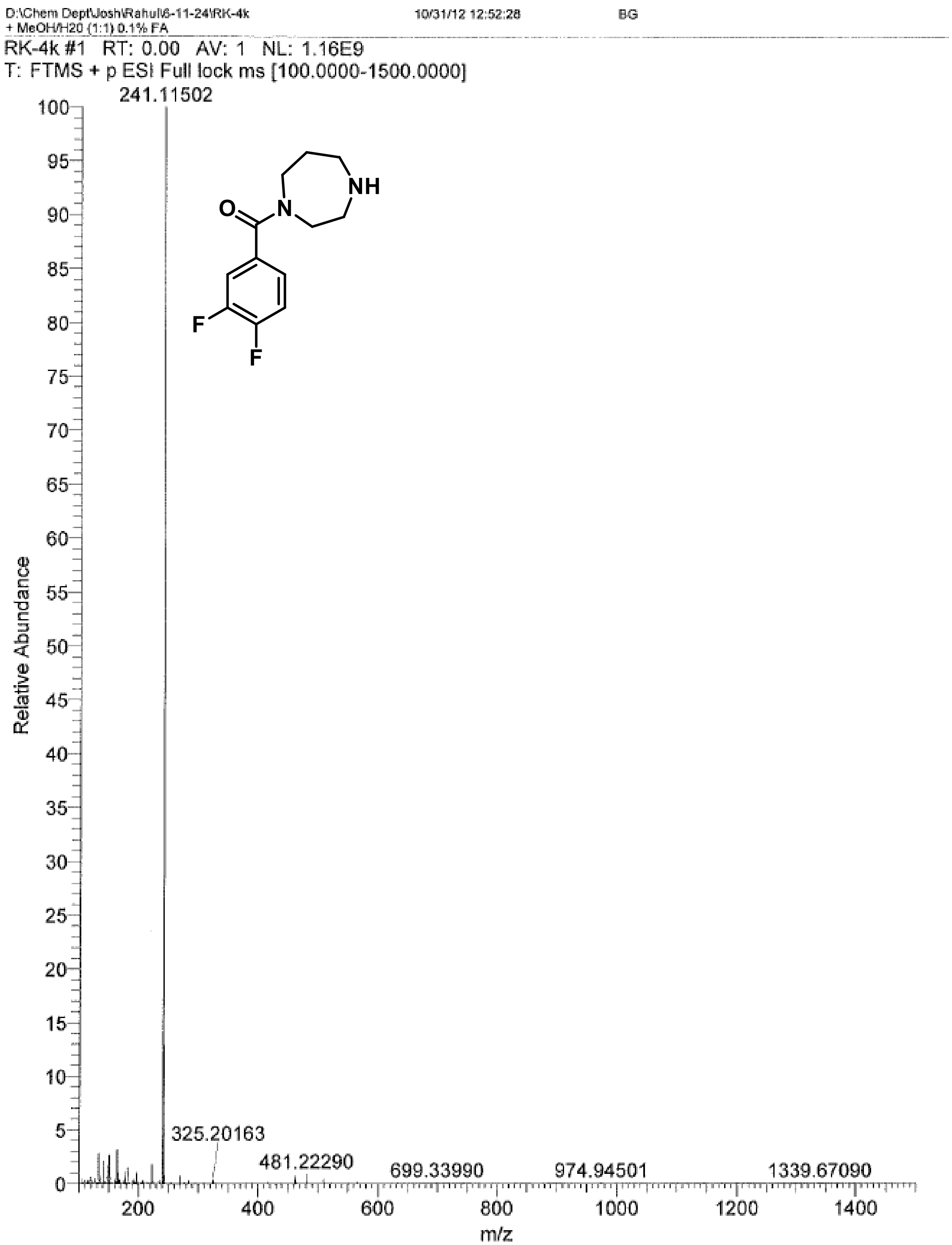
D:\Chem Dept\Josh\Rahul\6-11-24\RK-4k
+ MeOH/H20 (1:1) 0.1% FA
10/31/12 12:52:28
BG
RK-4k #1 RT: 0.00 AV: 1 NL: 1.16E9
T: FTMS + p ESI Full lock ms [100.0000-1500.0000]
241.11502
325.20163
481.22290
699.33990
974.94501
1339.67090
Relative Abundance
m/z
O
N
NH
F
F

(1,4-Diazepan-1-yl)(4-hydroxy-3-methoxyphenyl)methanone (4k)

Instrument 300B
1d_proton DMSO /opt/nmrdata nekkar 5

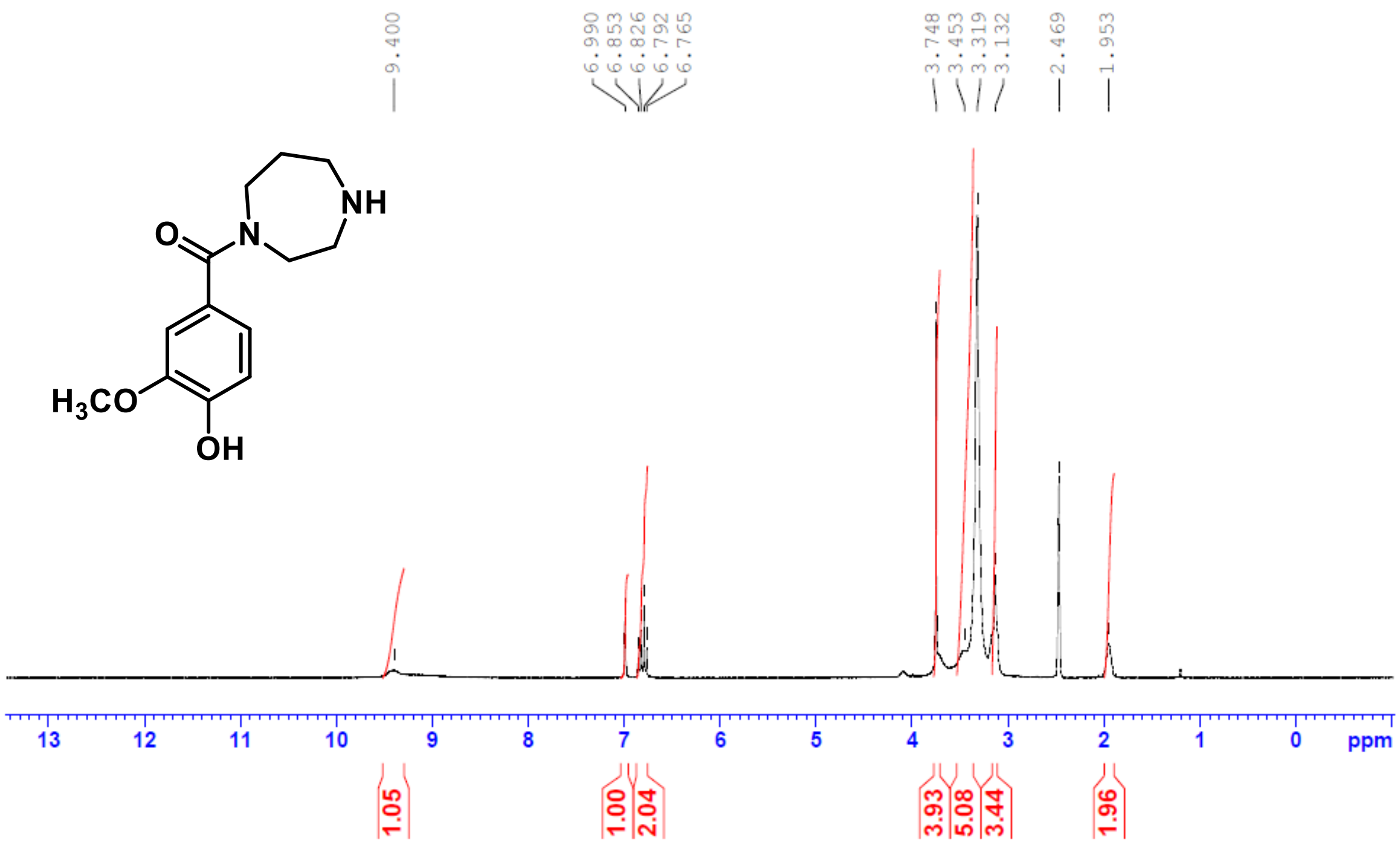

Instrument 300B
1D_C13_8Hours DMSO /opt/nmrdata nekkar 12

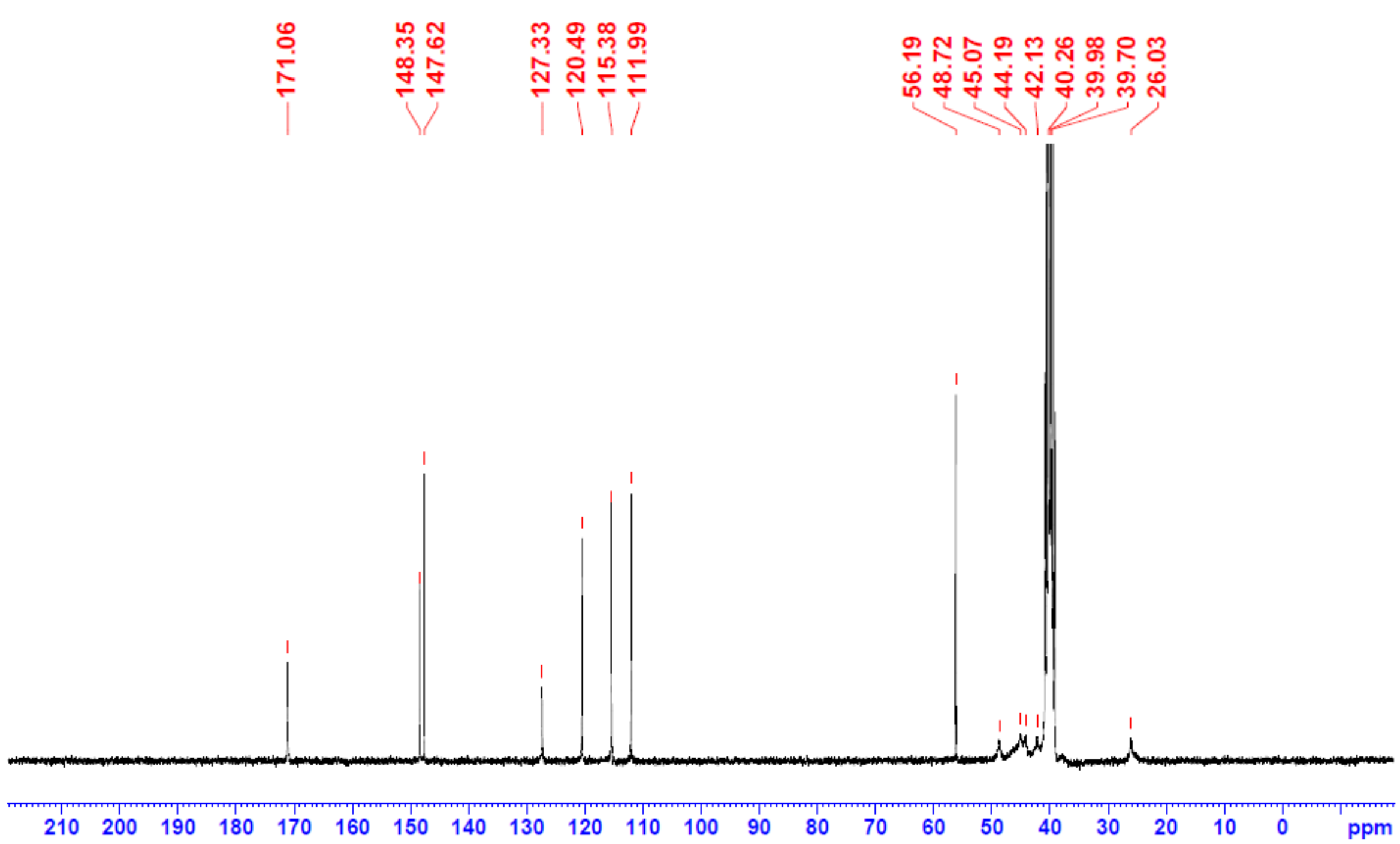

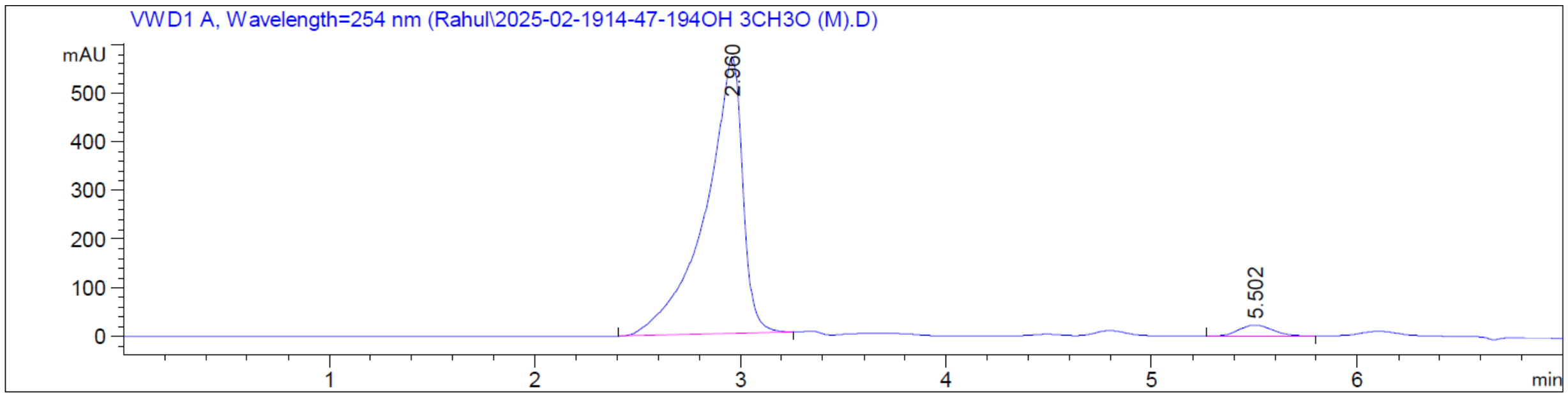

Signal 3: VWD1 A, Wavelength=254 nm

Peak #	RetTime [min]	Type	Width [min]	Area [mAU*s]	Height [mAU]	Area %
1	2.960	BB	0.1730	7160.88623	568.15906	96.5855
2	5.502	BB	0.1776	253.15067	22.44667	3.4145

Totals : 7414.03690 590.60573

D:\Pharmacy\...\Nov12 2024\Oct31_RK-4m 11/12/24 18:32:21
250.3

Oct31_RK-4m #42-64 RT: 0.23-0.34 AV: 23 NL: 1.89E9
T: FTMS + p ESI Full lock ms [100.0000-1200.0000]

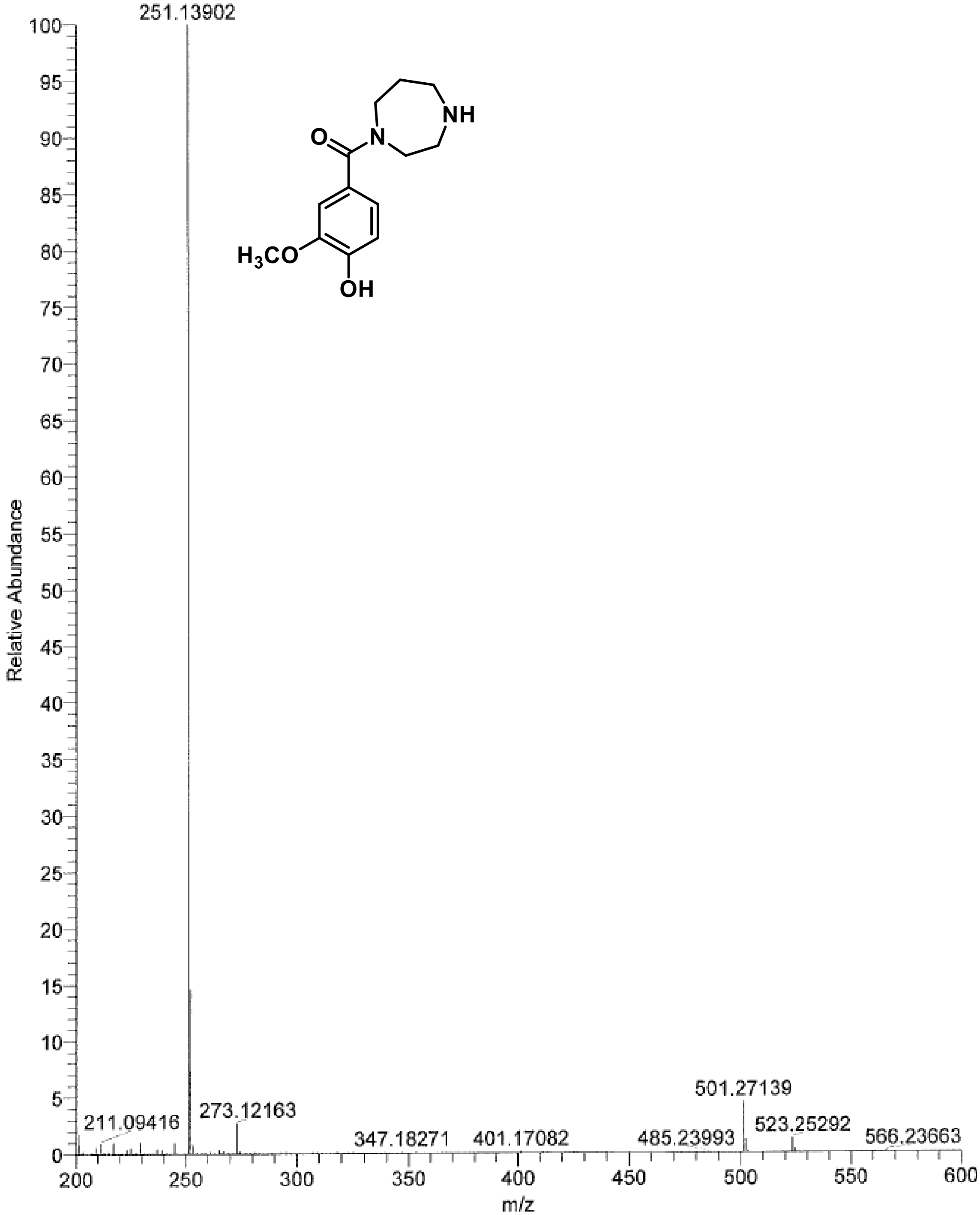

(1,4-Diazepan-1-yl)(3,4-dihydroxyphenyl)methanone (4l)

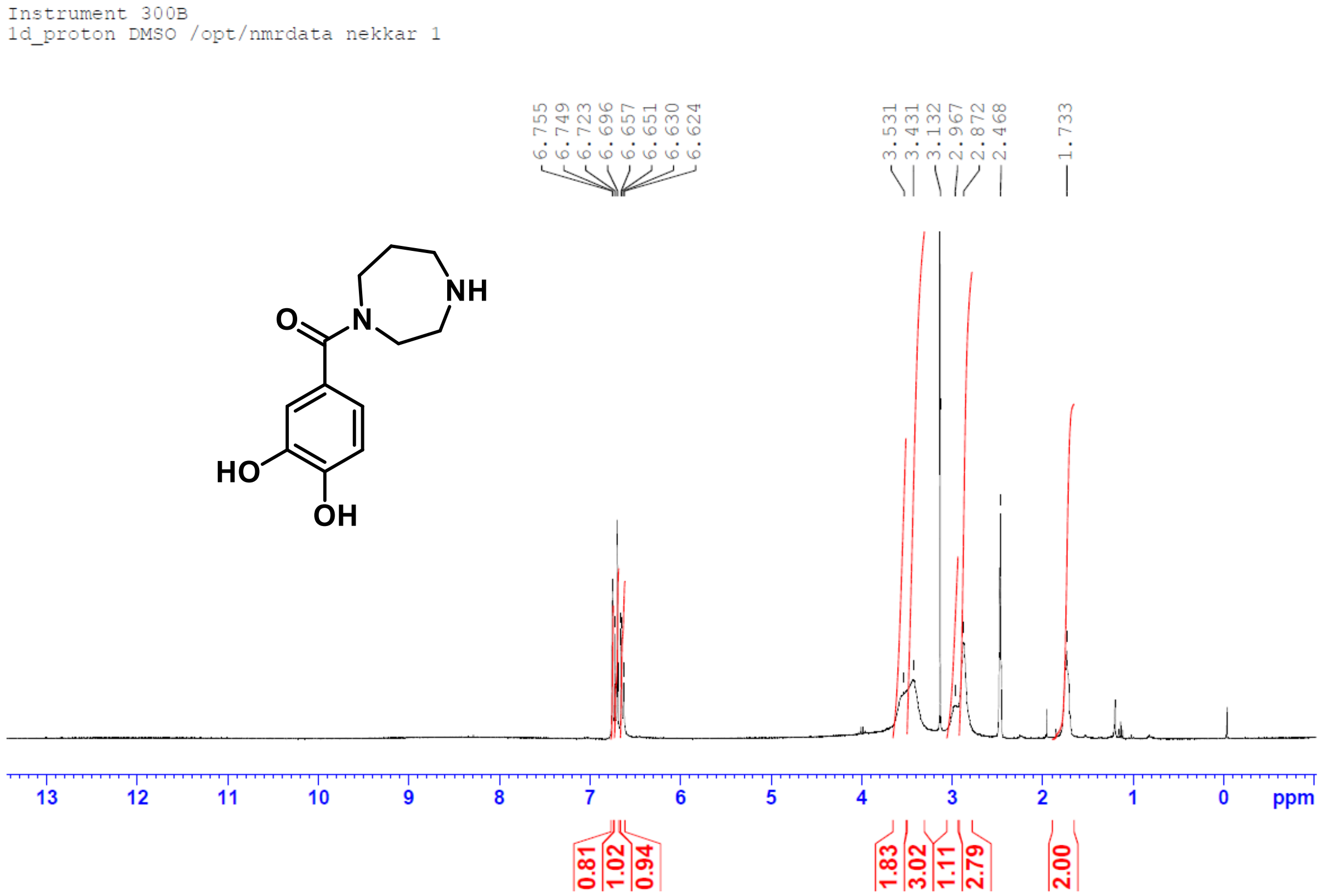

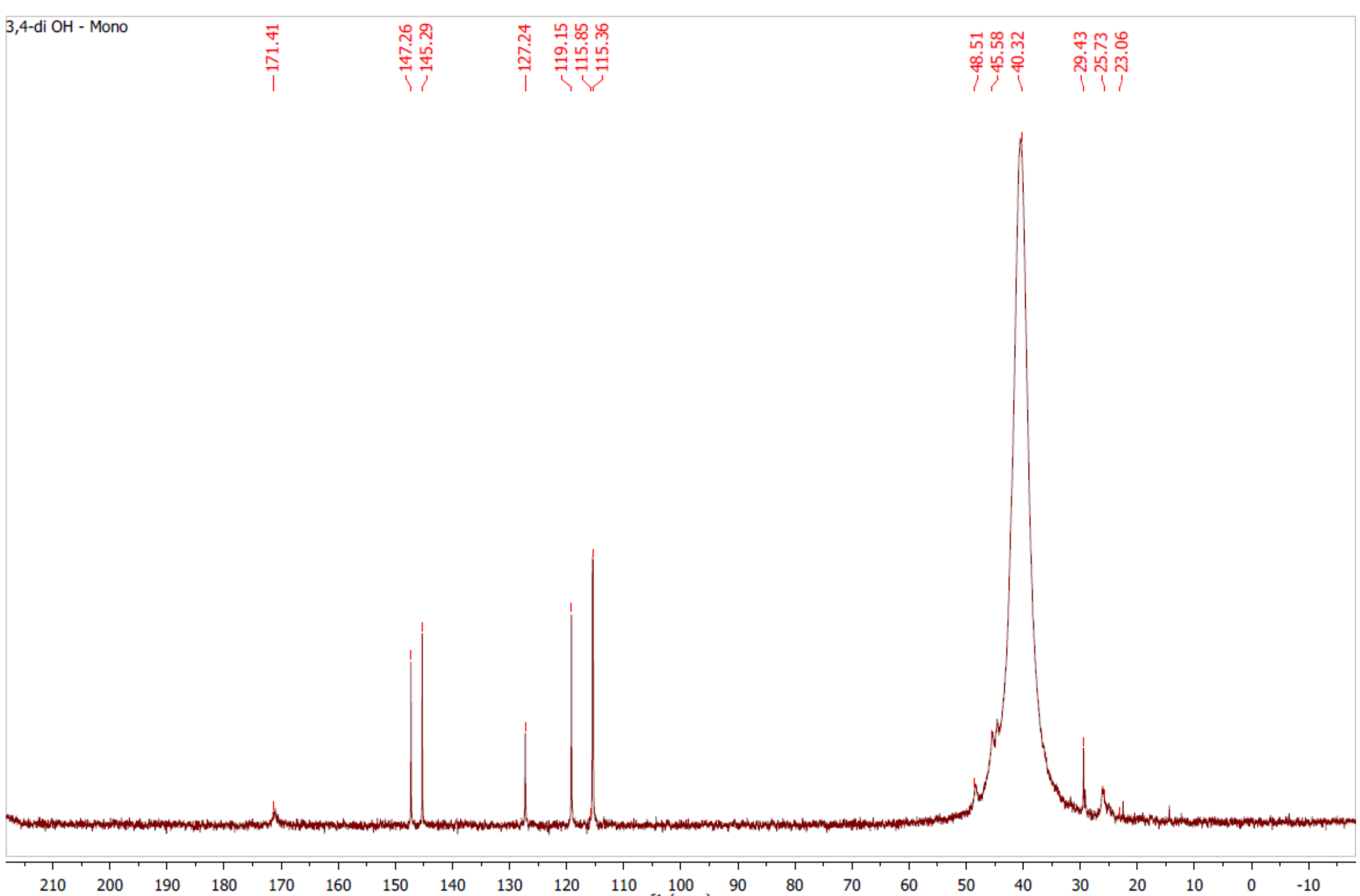

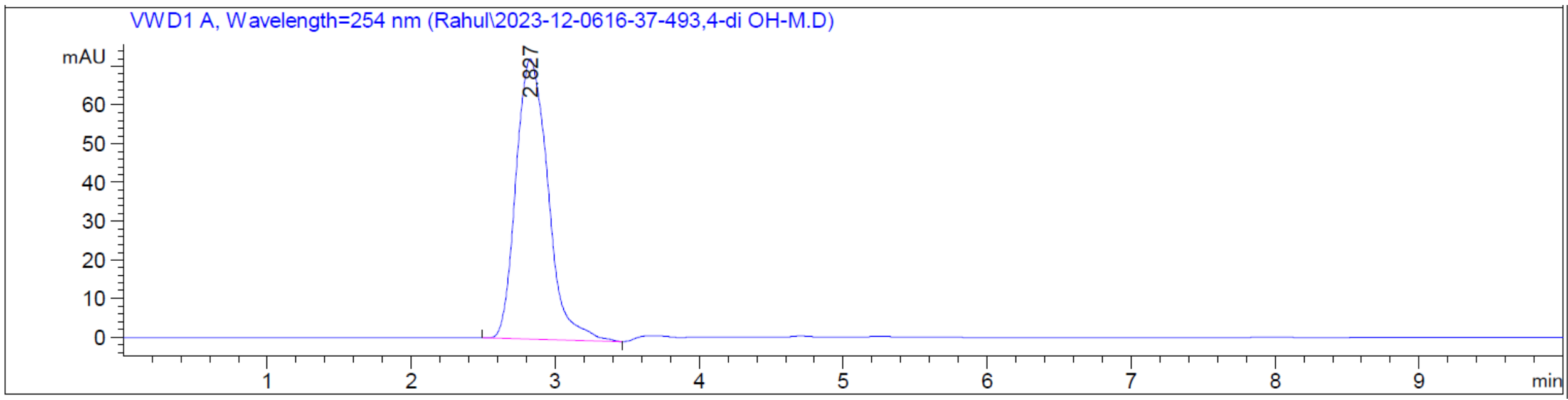

Signal 3: VWD1 A, Wavelength=254 nm

Peak #	RetTime [min]	Type	Width [min]	Area [mAU*s]	Height [mAU]	Area %
1	2.827	BB	0.2521	1117.99048	72.40628	100.0000

Totals : 1117.99048 72.40628

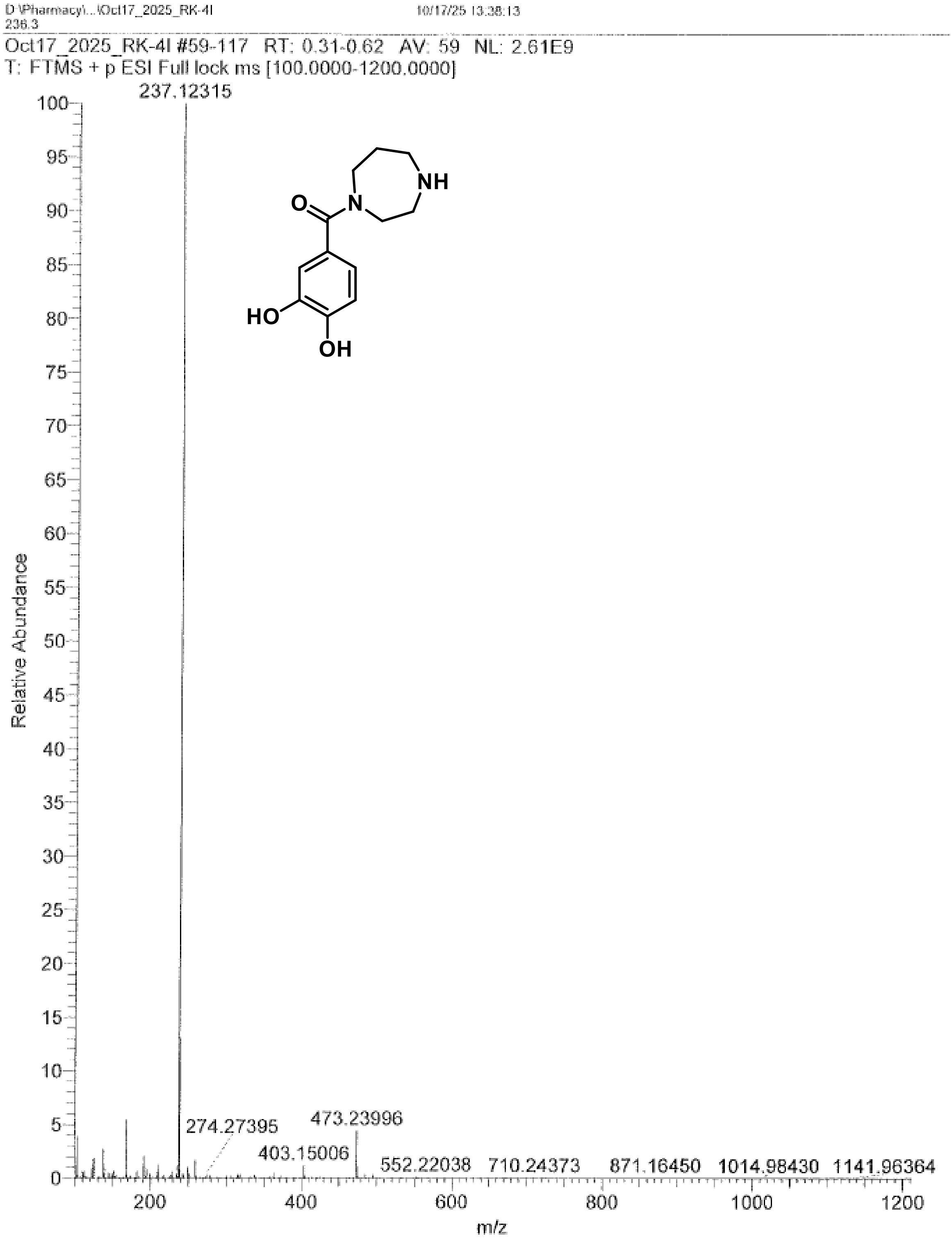

D:\Pharmacy\...\Oct17_2025_RK-4l
236.3
10/17/25 13:38:13
Oct17_2025_RK-4l #59-117 RT: 0.31-0.62 AV: 59 NL: 2.61E9
T: FTMS + p ESI Full lock ms [100.0000-1200.0000]
237.12315
O
N
NH
HO
OH
274.27395
473.23996
403.15006
552.22038
710.24373
871.16450
1014.98430
1141.96364
Relative Abundance
m/z

Benzo[*d*][1,3]dioxole-5-yl-(1,4-Diazepan-1-yl)methanone (4m)

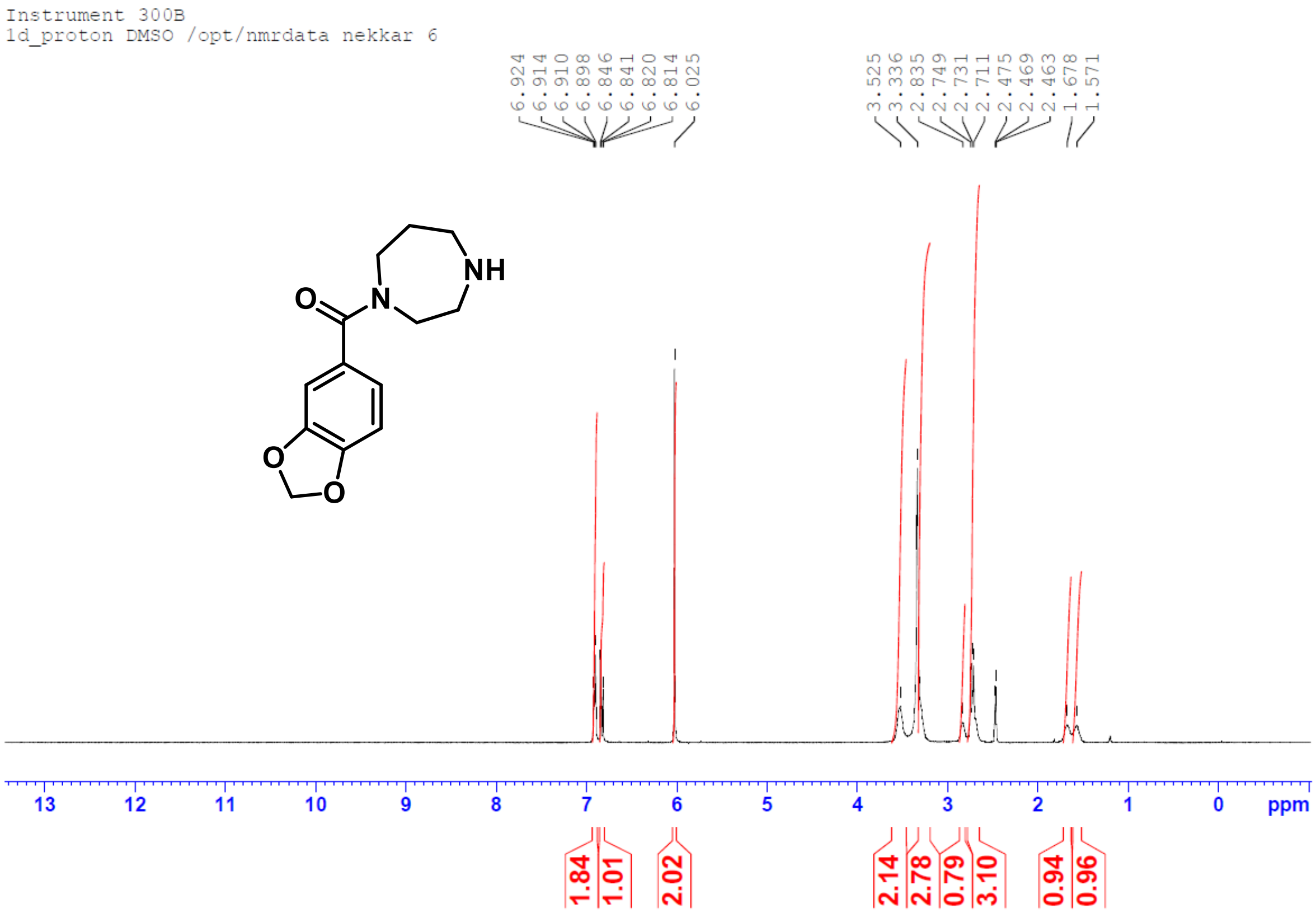

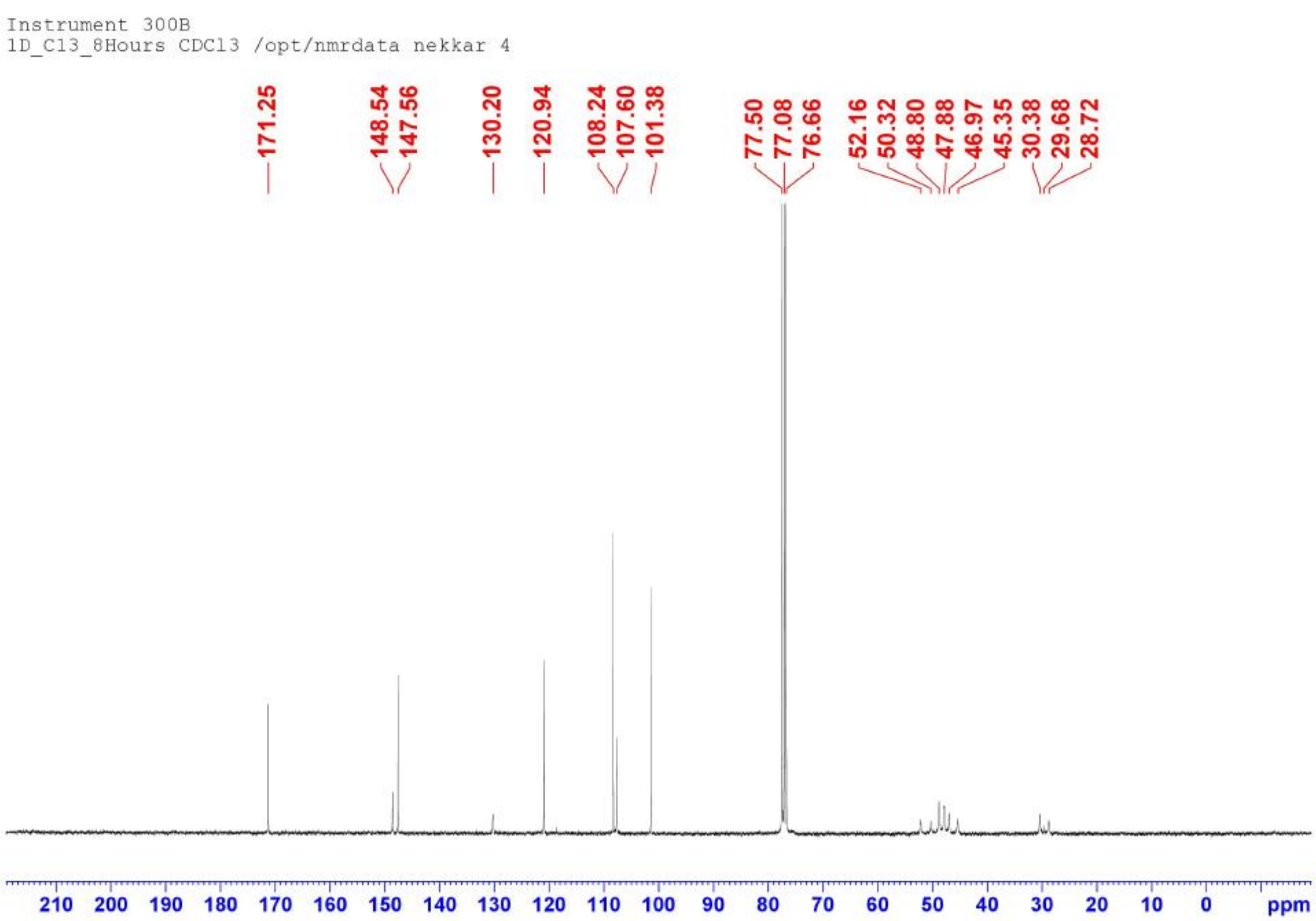

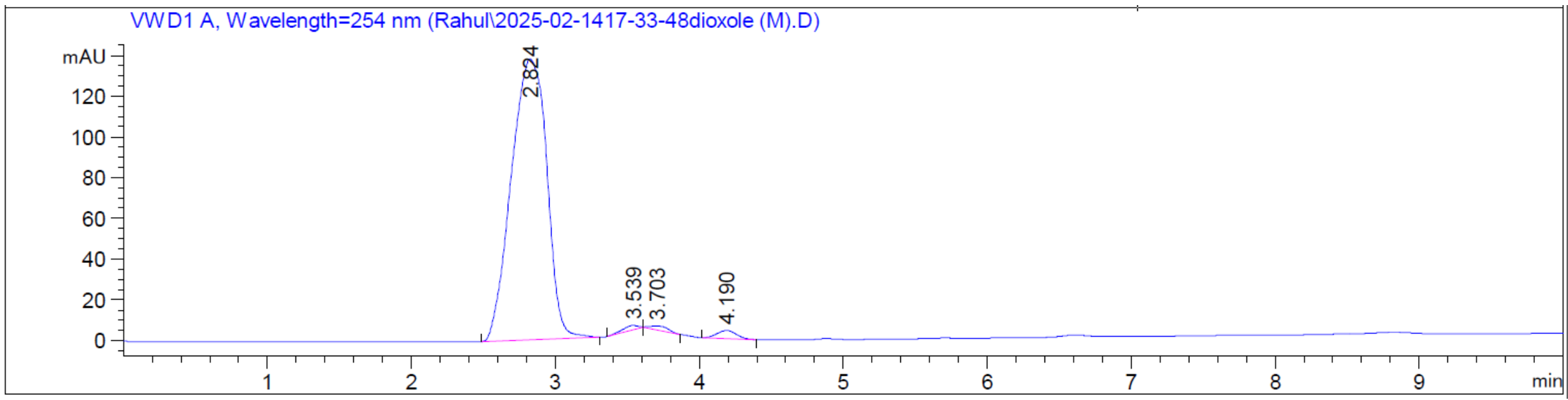

Signal 3: VWD1 A, Wavelength=254 nm

Peak #	RetTime [min]	Type	Width [min]	Area [mAU*s]	Height [mAU]	Area %
1	2.824	BB	0.2832	2367.56128	137.98224	97.1230
2	3.539	BB	0.1149	16.47201	2.13652	0.6757
3	3.703	BB	0.1495	16.82213	1.80810	0.6901
4	4.190	BB	0.1490	36.83927	3.94218	1.5112
Totals :				2437.69470	145.86904	

D:\Pharmacy\...\Nov12 2024\Oct31_RK-4i 11/12/24 18:11:11
248.3

Oct31_RK-4i #45-64 RT: 0.24-0.34 AV: 20 NL: 6.35E9
T: FTMS + p ESI Full lock ms [100.0000-1200.0000]

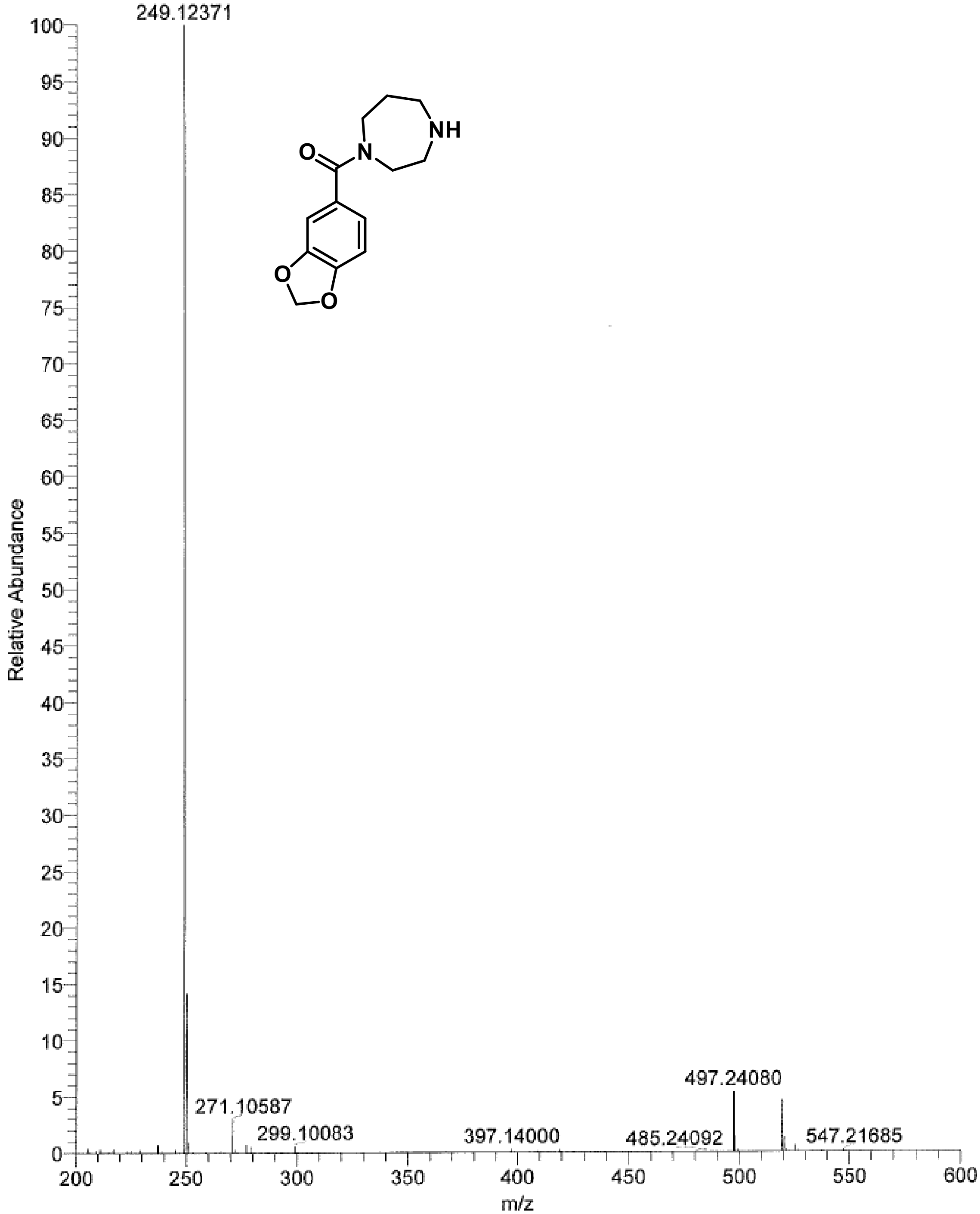

(4-Aminophenyl)(1,4-diazepan-1-yl)methanone (4n)

Instrument 300B
1d_proton DMSO /opt/nmrdata nekkar 2

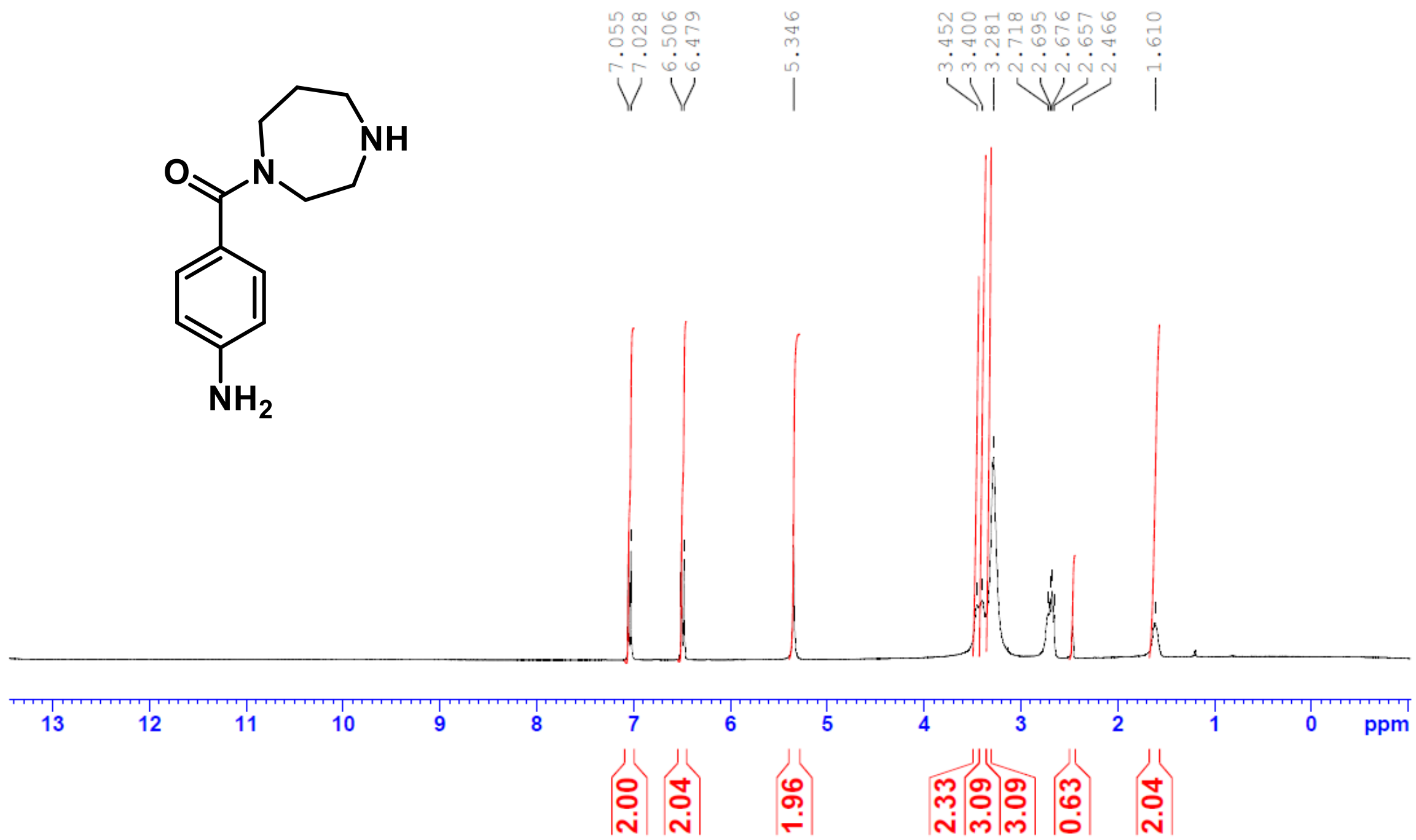

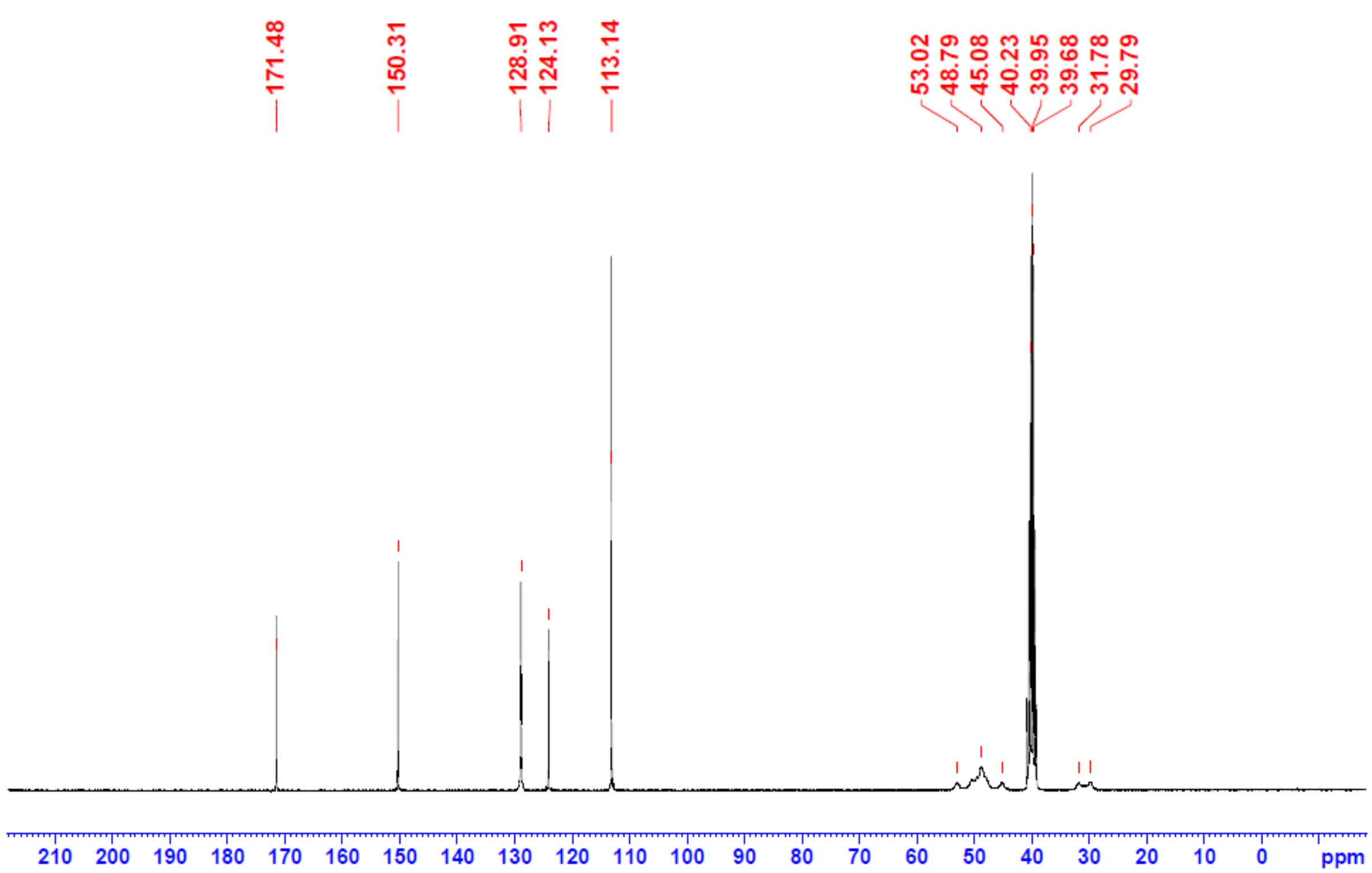

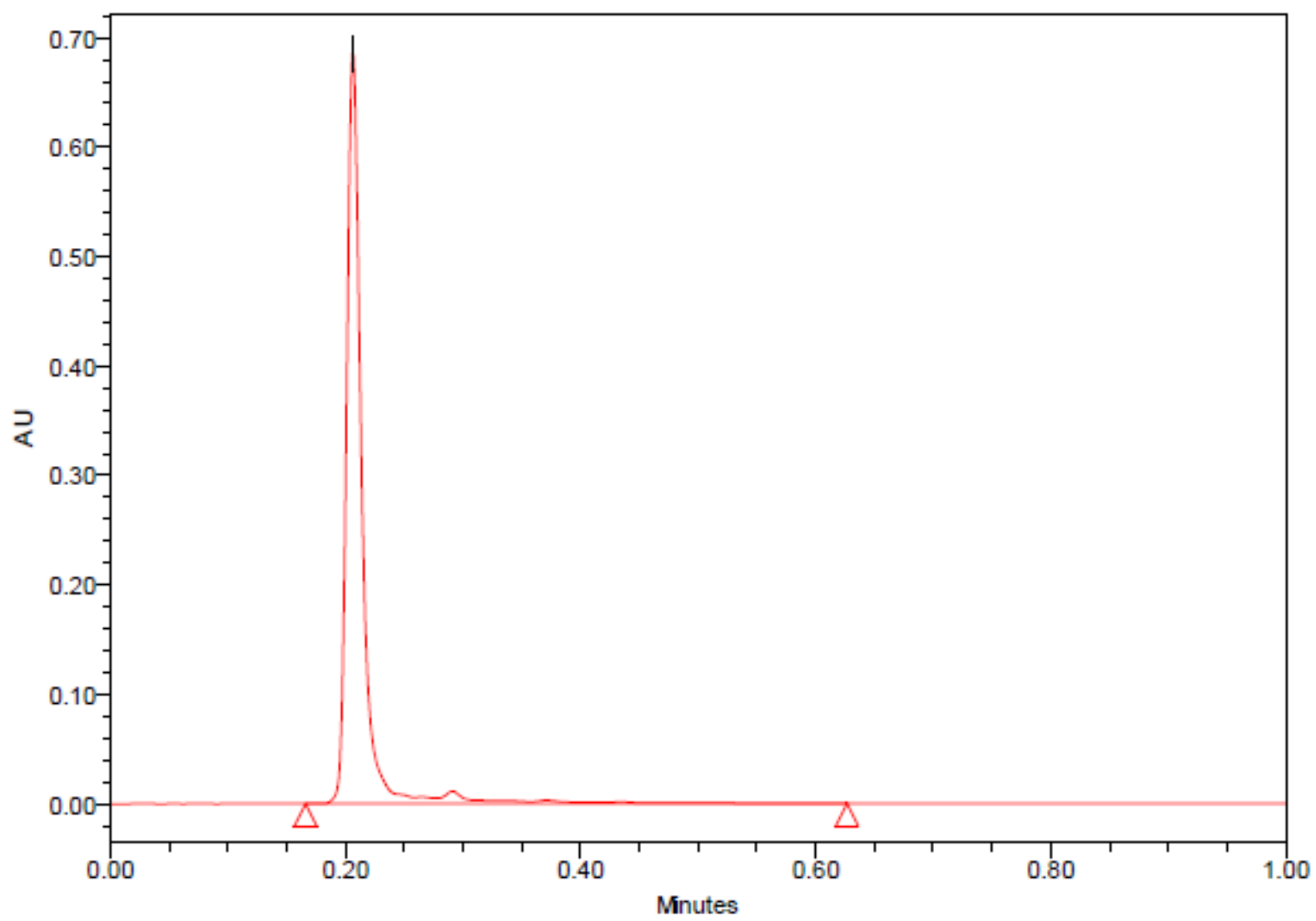

Peak Name:

	Injection	RT	Area	% Area
1	1	0.206	635448	100.00
Mean		0.206		
Std. Dev.				
% RSD				

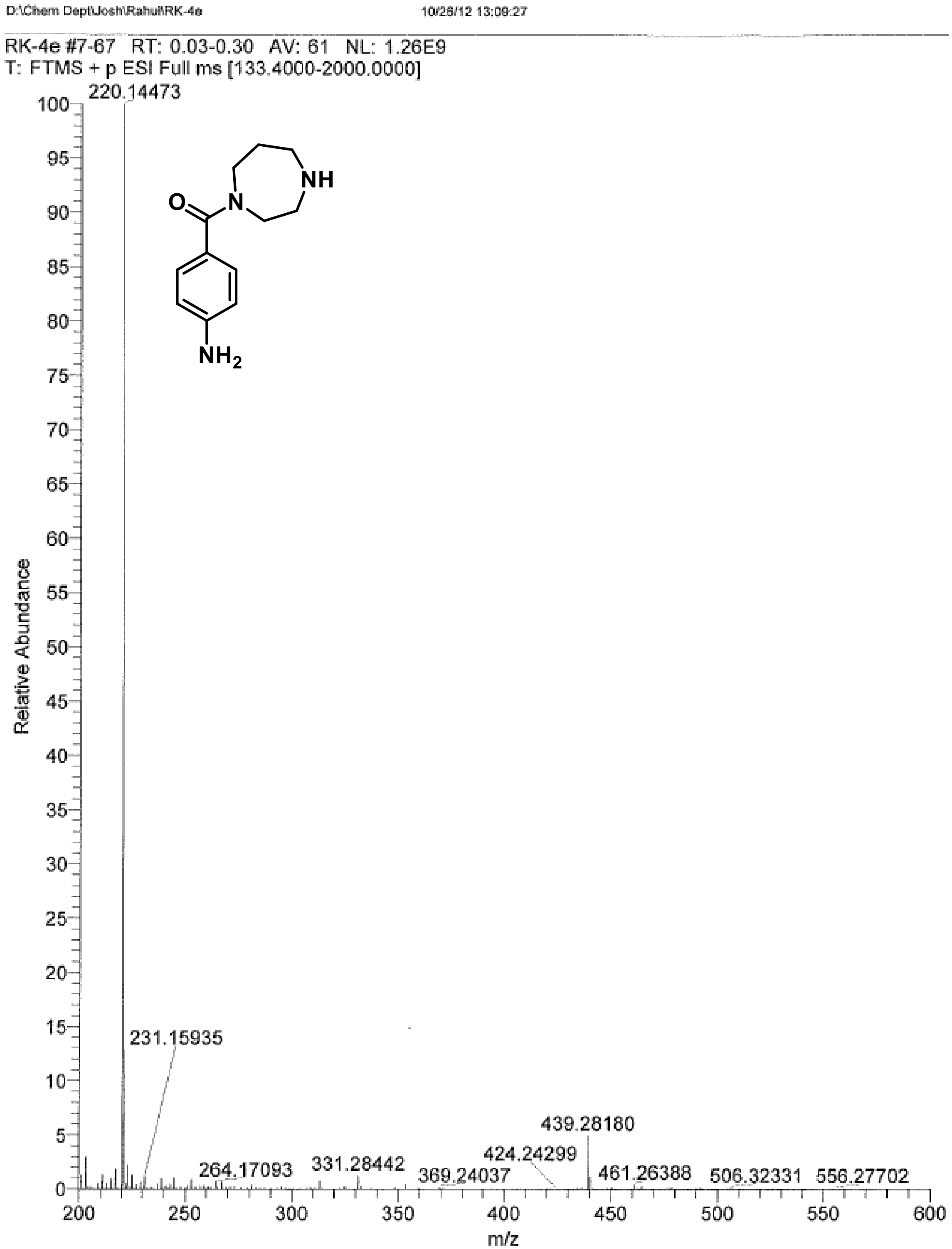

D:\Chem Dept\Josh\Rahul\RK-4e
10/26/12 13:09:27
RK-4e #7-67 RT: 0.03-0.30 AV: 61 NL: 1.26E9
T: FTMS + p ESI Full ms [133.4000-2000.0000]
220.14473
231.15935
264.17093
331.28442
369.24037
424.24299
439.28180
461.26388
506.32331
556.27702
Relative Abundance
m/z
O
N
NH
NH2

3 Evaluation of compounds 4a–n as inhibitors of Aβ40 aggregation at 25 µM

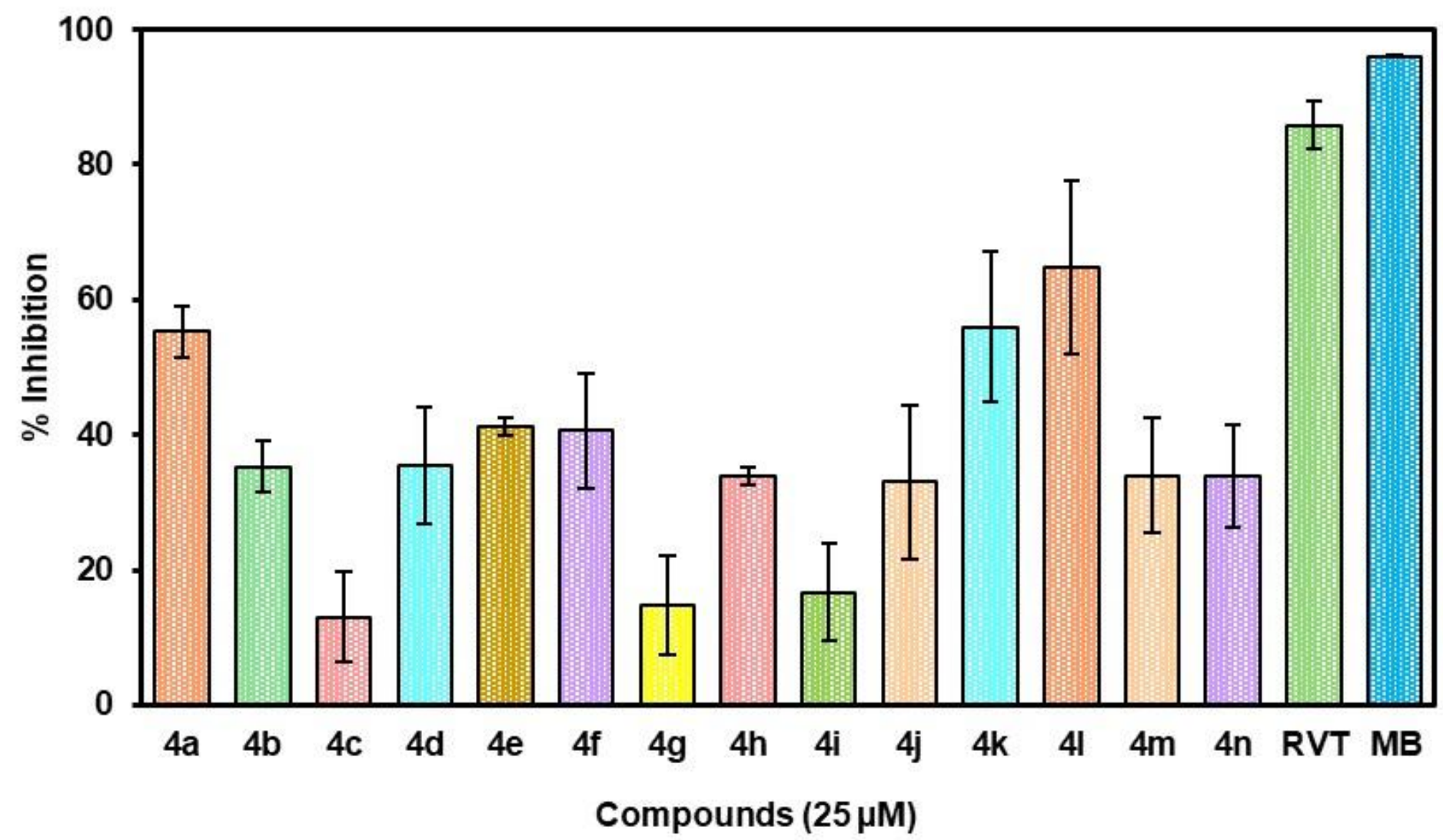

Figure S1. ThT fluorescence-based assessment of Aβ40 aggregation inhibition by (1,4-diazepan-1-yl)(phenyl)methanone derivatives (**4a–n**), RVT, and MB (25 µM each). The ThT assay was conducted with Aβ40 (10 µM) at pH 7.4, 37 °C for 24 h. Results represent the average of duplicate readings from two independent experiments.

4 Evaluation of compound 4k as inhibitor of Aβ40 aggregation

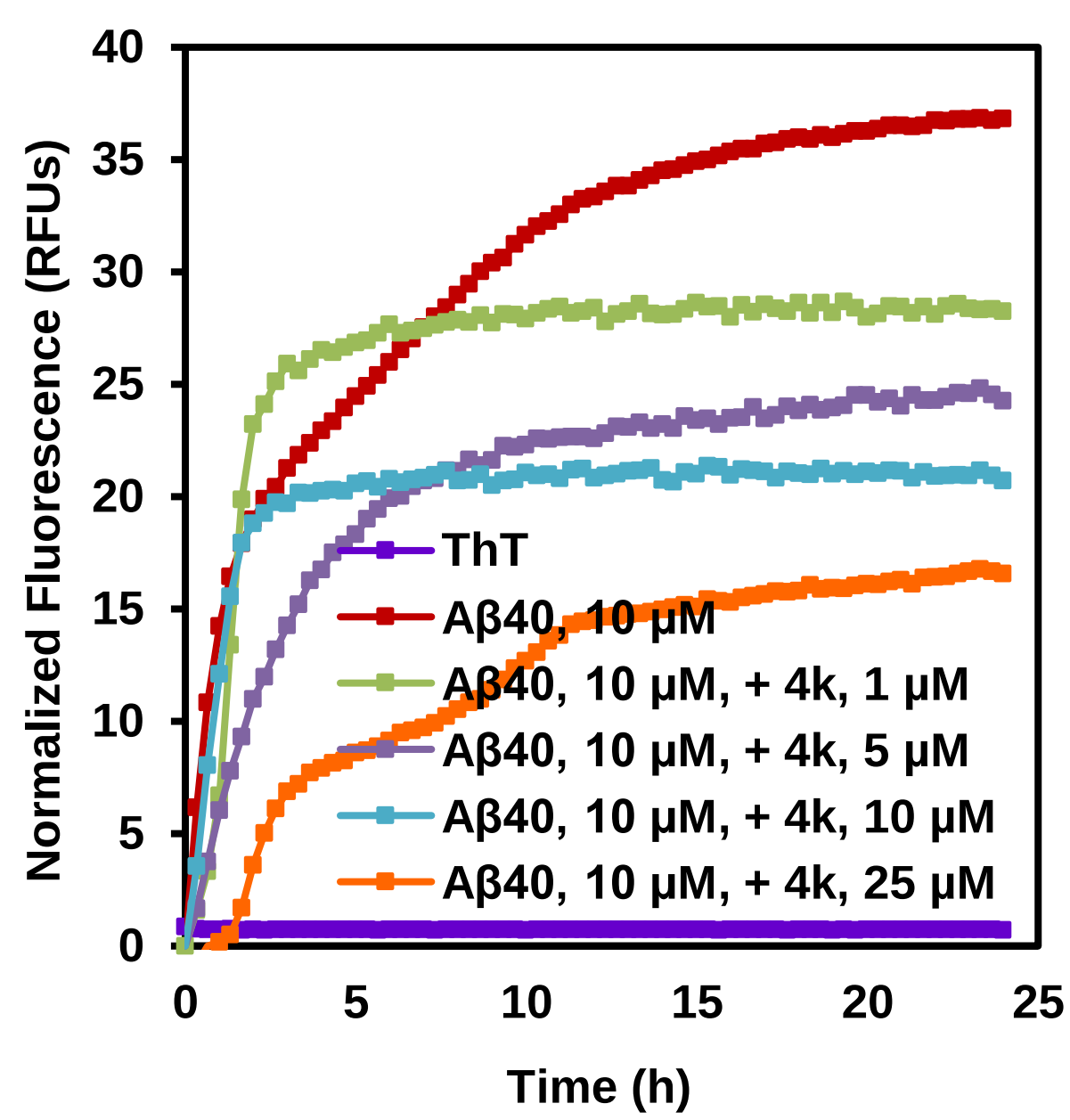

Figure S2. ThT fluorescence-based monitoring of Aβ40 aggregation kinetics in the presence of compound **4k** at 1, 5, 10, and 25 µM. The aggregation assays were performed using Aβ40 (10 µM) at pH 7.4, 37 °C for 24 h. ThT fluorescence was recorded (excitation at 440 nm and emission at 490 nm), and the results are presented as average ± SD from three independent experiments (n = 3).

5 DPPH Scavenging by compounds 4a, 4k, and 4l

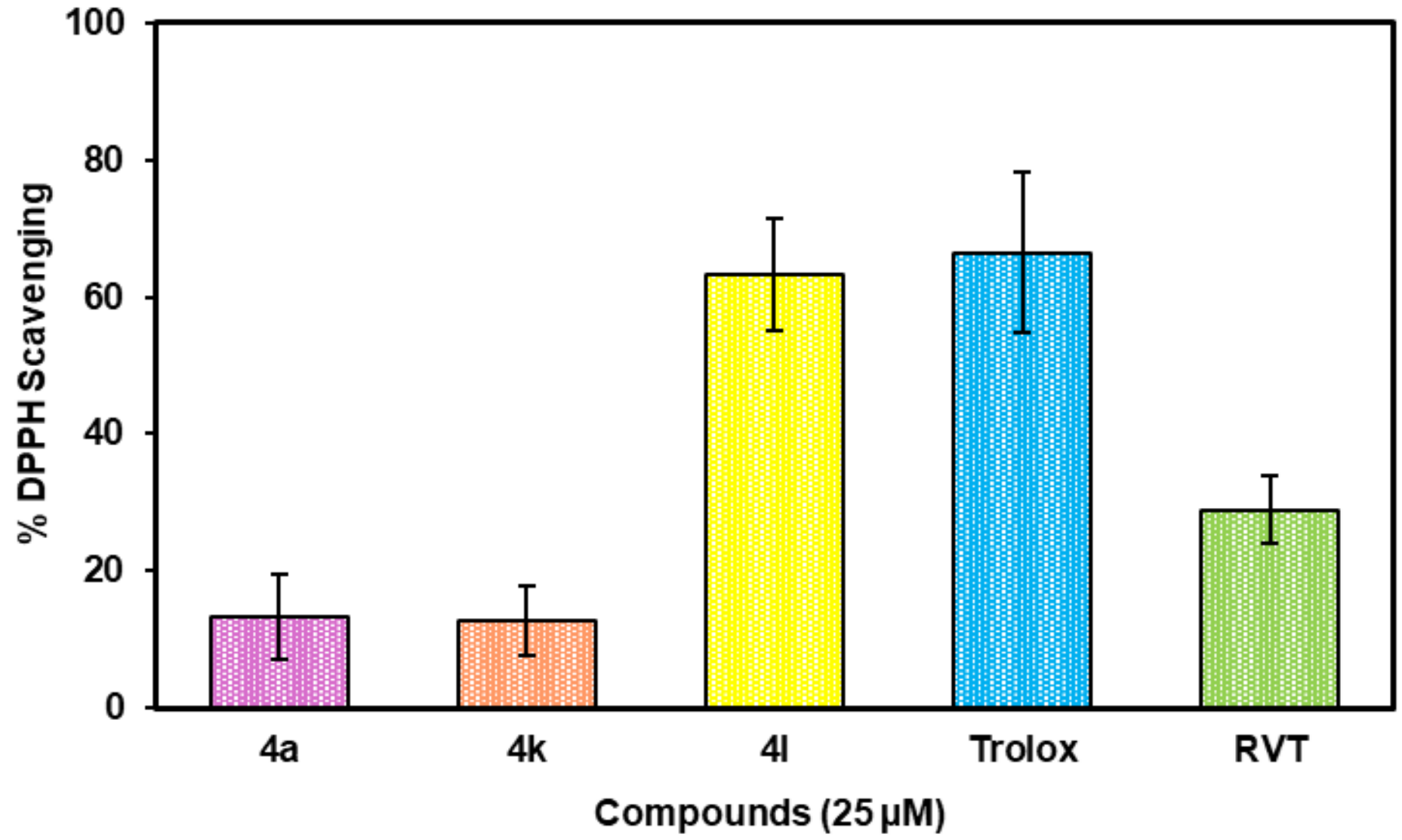

Figure S3. DPPH radical scavenging activity of compounds **4a**, **4k**, **4l**, RVT, and trolox at 25 µM. The results represent the mean ± SD from triplicate measurements (n = 3) from two independent experiments.

6 BBB Permeability Assay Data for Compounds 4a, 4g, 4k, 4l, 4m, and RVT

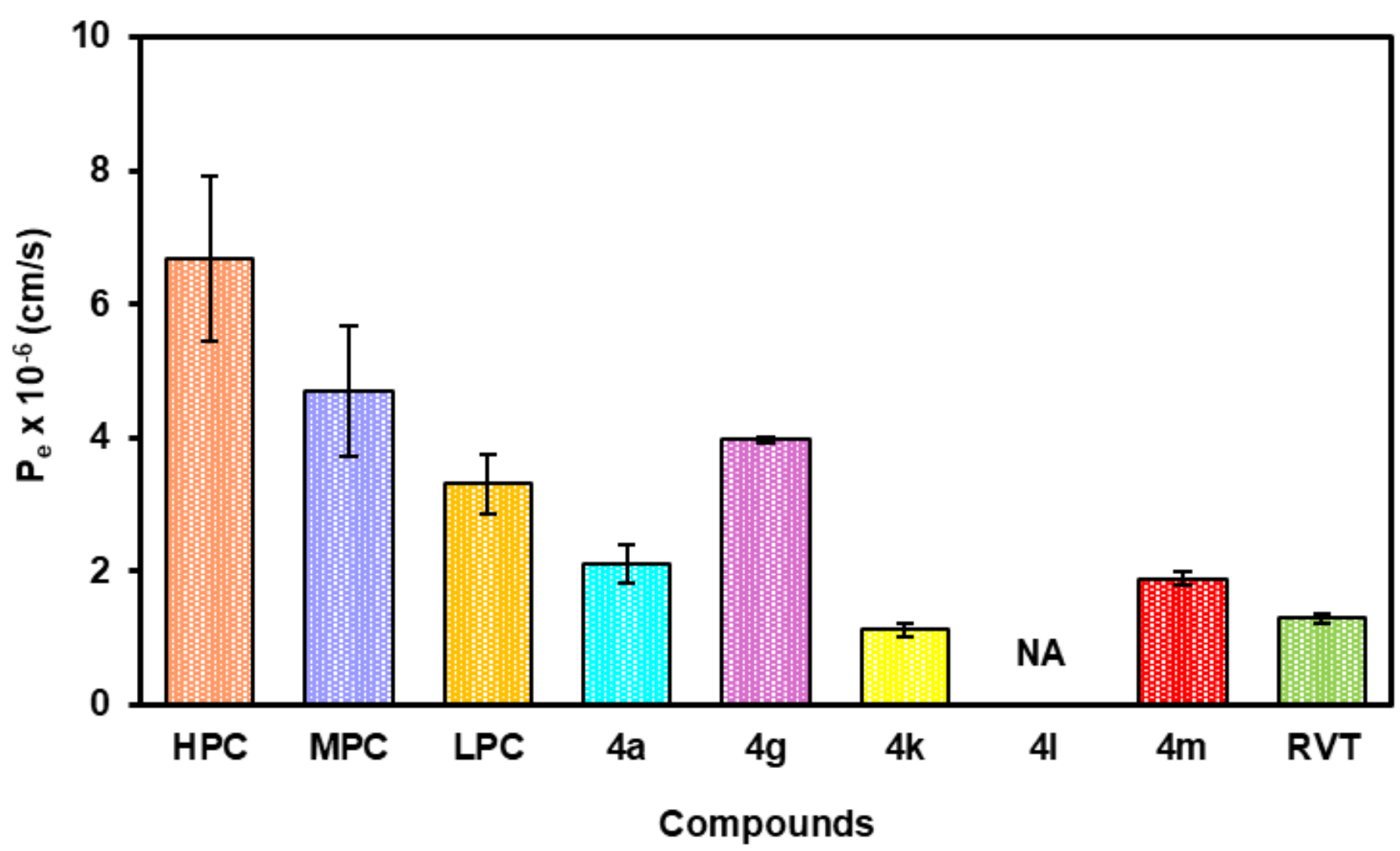

Figure S4. PAMPA-BBB permeability assay of compounds **4a**, **4g**, **4k**, **4l**, **and 4m** after 18 h incubation at 37 °C, including high, medium, and low (HPC, MPC and LPC) permeability controls. Data represents average of duplicate measurements from two independent experiments (n = 2).

Table S1. Permeability coefficient (P_e) of **4a**, **4g**, **4k**, **4l**, and **4m** in the PAMPA-BBB assay.

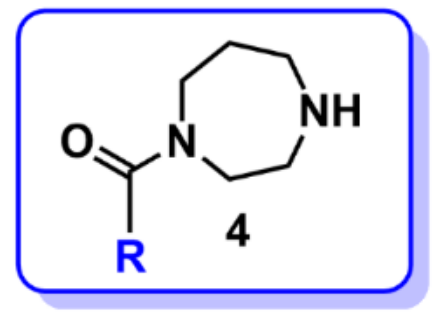

Compound	R	Permeability Coefficient (P_e x 10^{-6} cm/s) [a]	ClogP [b]
**HPC (Promazine)**	–	6.68 ± 1.2	4.59
**MPC (Clonidine)**	–	4.69 ± 1.0	1.72
**LPC (Diclofenac)**	–	3.31 ± 0.5	4.57
**4g**	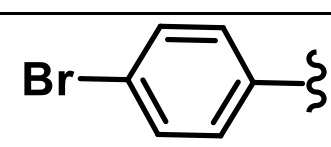	3.97 ± 0.0	1.57
**4k**	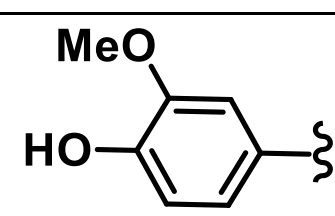	1.13 ± 0.1	0.32
**4l**	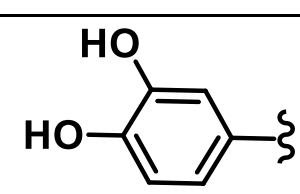	NA	0.07
**4m**	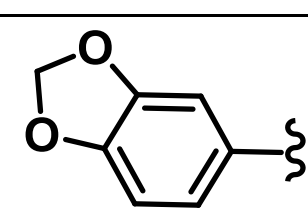	1.89 ± 0.1	0.83
**RVT**		1.30 ± 0.1	2.83

[a] P_e values are presented as average of duplicate readings from two independent experiments. [b] ClogP values were calculated using ChemDraw 23.1.2. HPC - high permeability control, MPC - moderate permeability control, and LPC - low permeability control.

7 **Table S2.** Summary of physicochemical and drug-likeness properties of **4k** and **4m** calculated from Web Tool SwissADME.

	**Parameter Name**	**4k**	**4m**
**Physicochemical property**	**Molecular Weight (g/mol)**	250.29	248.28
	**Rotatable bonds**	3	2
	**H-bond Acceptors**	4	4
	**H-bond Donors**	2	1
	**TPSA (Å^2) [a]**	61.8	50.8
	**BBB permeability**	Yes	Yes
	**ClogP [b]**	0.32	0.83
**Druglikeness**	**Lipinski**	Yes, 0 violation	Yes, 0 violation
	**PAINS [c]**	0 alert	0 alert

[a] TPSA: Topological polar surface area; [b] ClogP: Calculated partition coefficient from ChemDraw software; [c] PAINS: pan-assay interference compounds.
